# Fine-grained descending control of steering in walking *Drosophila*

**DOI:** 10.1016/j.cell.2024.08.033

**Published:** 2024-09-17

**Authors:** Helen H. Yang, Bella E. Brezovec, Laia Serratosa Capdevila, Quinn X. Vanderbeck, Atsuko Adachi, Richard S. Mann, Rachel I. Wilson

**Affiliations:** 1 Department of Neurobiology, Harvard Medical School, Boston, MA 02115 USA; 2 Department of Neurobiology, Stanford University, Stanford, CA 94305 USA; 3 Aelysia LTD, Bristol, BS9 3BY UK; 4 Department of Biochemistry and Molecular Biophysics, Department of Neuroscience, Zuckerman Mind Brain Behavior Institute, Columbia University, New York, NY 10027 USA; 5 Current affiliation: Princeton Neuroscience Institute, Princeton University, Princeton, NJ 08540 USA; 6 Lead contact

## Abstract

Locomotion involves rhythmic limb movement patterns that originate in circuits outside the brain. Purposeful locomotion requires descending commands from the brain, but we do not understand how these commands are structured. Here we investigate this issue, focusing on the control of steering in walking *Drosophila*. First, we describe different limb “gestures” associated with different steering maneuvers. Next, we identify a set of descending neurons whose activity predicts steering. Focusing on two descending cell types downstream from distinct brain networks, we show that they evoke specific limb gestures: one lengthens strides on the outside of a turn, while the other attenuates strides on the inside of a turn. Our results suggest that a single descending neuron can have opposite effects during different locomotor rhythm phases, and we identify networks positioned to implement this phase-specific gating. Together, our results show how purposeful locomotion emerges from specific, coordinated modulations of low-level patterns.

## Introduction

**Figure 1.**
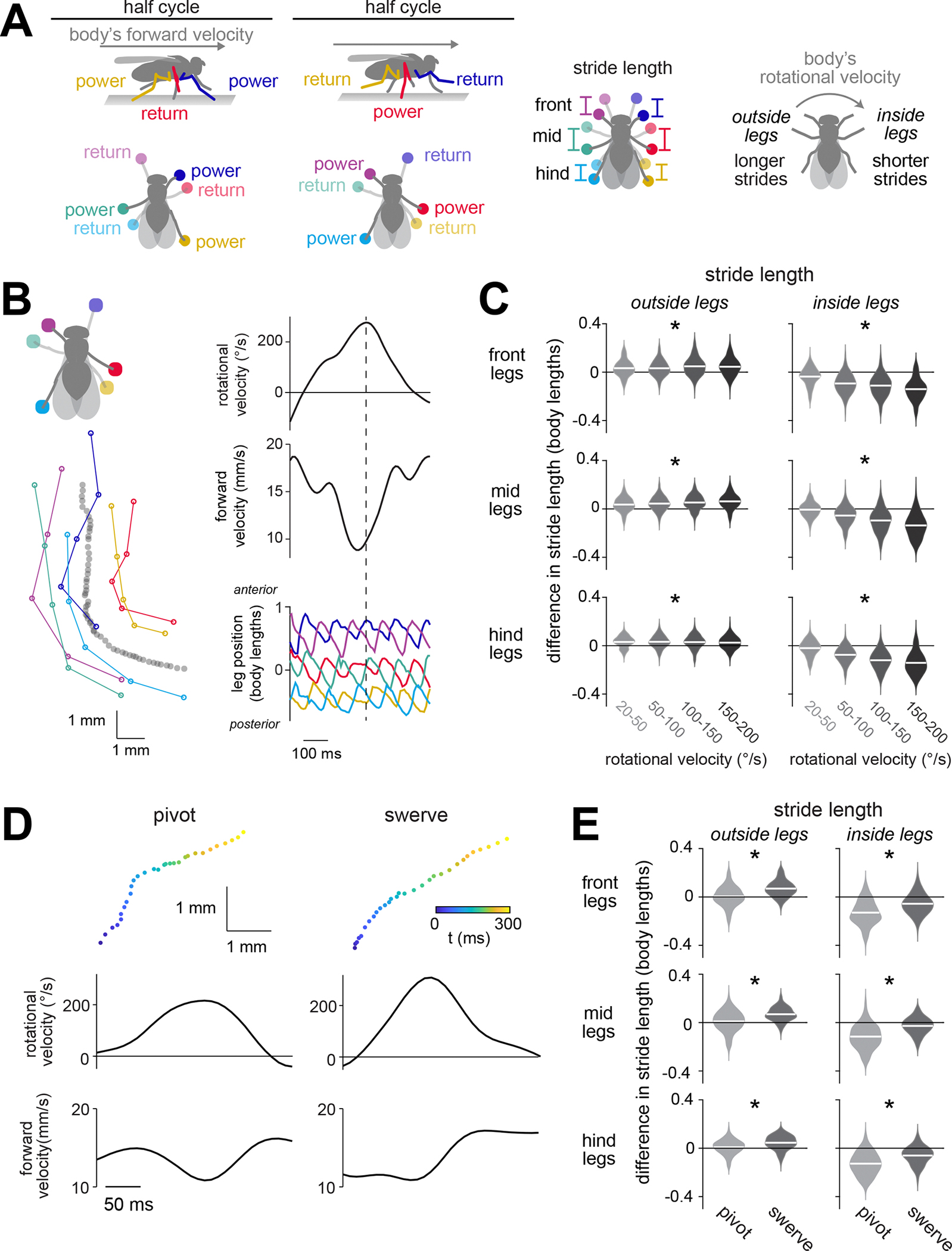
Different steering maneuvers use different leg gestures (A) During the power stroke, the leg pushes backward. During the return stroke, the leg swings forward to a new position. Schematics below show each leg at the endpoint of its power and return strokes. Stride length is the difference between the endpoints in the anterior-posterior axis. (B) *Left*: example steering bout, showing the path of the fly’s body (gray) and the positions of the leg tips at the end of the return stroke. *Right*: the fly’s rotational and forward body velocity and the anterior-posterior position of the leg tips in body-centric coordinates. Dotted line marks the peak of the turn. (C) When flies rotate, stride length decreases for the inside legs, while increasing for the outside legs. For each turning bout, we measured the bout’s peak rotational velocity and the stride length for the step that occurred at that peak. Stride lengths are expressed as the change from the mean when the fly was not turning. Stride length varies significantly with rotational velocity for both outside and inside legs. Violin plots are Gaussian kernel estimates fit to all data points. n = 138 (55), 454 (68), 434 (68), and 151 (50) bouts (flies) for 20–50, 50–100, 100–150, and 150–200 °/s, respectively. Asterisks mark legs with significant changes. (D) *Top:* paths for example steering bouts, corresponding to a pivot and a swerve. *Bottom:* rotational and forward velocity for these examples. Pivots are bouts where forward velocity decreases by >1 mm/s; swerves are bouts where forward velocity increases by >1 mm/s. In swerves, because forward velocity is high, the curvature of the path is more subtle than it is for pivots. (E) Pivots and swerves produce different changes in stride length (n = 389 (64) and 155 (53) bouts (flies), respectively). See also Figure S1. See Table S1 for statistics.

Vertebrates and arthropods are unique among living creatures in their ability to walk. All walking organisms confront the same problem — namely, how to keep the body stably raised above the ground, while also propelling the body at the intended speed and steering along the intended path. The solution is to use different limbs for propulsion at different moments, in a rhythmic cycle (Figure 1A). Each limb alternates between a power stroke (stance) and a return stroke (swing). In the power phase, the limb is in contact with the ground, where it exerts propulsive force. In the return phase, the limb swings to a new position. Forward speed control emerges from symmetric modulations of this limb movement pattern, whereas steering emerges from asymmetric modulations (Figure 1A).

These modulations require cooperation among several regions of the central nervous system. Specifically, the rhythmic limb movement pattern is intrinsic to the vertebrate spinal cord^1,2^ or the arthropod ventral nerve cord^3–7^. Meanwhile, descending signals from the brain to the cord are required to start or stop locomotion, modulate speed, or change direction in both vertebrates and arthropods^8–12^. Yet the brain’s role in locomotor control is not necessarily limited to high-level commands: blocking descending signals from the brain can also alter the details of limb coordination during walking in vertebrates^13–15^ and arthropods^7,16^, and stimulation of specific descending tracts can produce excitation or inhibition of muscle groups in multiple limbs^17–21^. Currently, we do not understand what features of locomotion are actually under the brain’s control.

In considering this problem, it is useful to invoke the concept of elemental components. In a puppet, the elemental components of control are the strings, and there are fewer strings than joints because the puppeteer is concerned with evoking naturalistic gestures, not arbitrarily controlling every joint; similarly, the brain may have a limited number of control “strings”^22^. Circuits in the spinal cord or ventral nerve cord can control sets of co-activated muscles, and so these muscle synergies are often treated as the elemental components of control. However, from the brain’s perspective, the elements of motor control might be more high-level than muscle synergies. Once we can map the brain’s strings onto specific descending cell types, we should be better positioned to understand the logic of control.

In this regard, *Drosophila* offers unique advantages: it is the only limbed animal with a near-complete connectome^23–28^, and many *Drosophila* descending neurons (DNs) involved in walking control are uniquely identifiable as individual right-left cell pairs^29–35^. Moreover, genetic tools^29,36^ allow us to target single DNs for recording and perturbation during locomotion.

Here, we use walking *Drosophila* to focus on the logic of steering control. Steering must arise from right-left asymmetries in limb movements in walking *Drosophila*^37,38^ and other hexapods^39–47^, just as in tetrapods and bipeds^2,48^. Currently, we know relatively little about the descending control of steering in any species, aside from the fact that descending signals are asymmetric during a turning bout^20,31,32,49–57^. A recent study found that multiple DNs in walking flies were correlated with steering direction but not with limb movements^57^; however, it is curious that there would be multiple DNs driving steering if all these cells are merely carrying redundant copies of a command to turn right or left.

Our study has three parts. First, we identify different limb movement gestures associated with different types of steering maneuvers. Second, we identify a set of DNs whose activity is correlated with steering; this provides a potential neural substrate for the elemental control of limb gestures. Third — in the main focus of the study — we show that different steering DNs evoke distinct limb gestures and that they are recruited by essentially non-overlapping pathways in the brain. Moreover, our results provide evidence that a single DN can have opposite effects on leg movement during different phases of the locomotor cycle, and we identify cells in the ventral nerve cord that are anatomically positioned to implement this phase-specific gating. Together, our results show how the brain can exert detailed but also efficient control of steering via selective recruitment of DNs dedicated to specific limb gestures. Our findings help re-cast the abstract problem of adaptive locomotor control as a more concrete problem of generating specific sequences of activity in populations of neurons in the brain.

## Results

### Different steering maneuvers use different leg gestures

To understand the descending control of steering, we first need to understand what variations in steering actually look like. *Drosophila* can perform several types of steering maneuvers, which are associated with distinct changes in the body’s rotational, lateral, and forward velocity^58,59^. Steering can also involve several different leg “gestures”^37,38^, defined here as coordinated changes in the movement of multiple limbs (Figure 1A). We wondered whether different types of steering maneuvers use systematically different leg gestures; if so, then these gestures might be elemental components of descending control.

To address this question, we analyzed the leg movements of freely walking flies^60^ using high-resolution, automated body-part tracking^61^ (Figures 1B and S1A). Specifically, we focused on turning bouts, defined by peaks in the body’s rotational velocity. We found that three asymmetrical, multi-leg gestures were associated with steering: increasing the stride length of the three legs on the outside of the turn (Figure 1C), decreasing the stride length of the three legs on the inside of the turn (Figure 1C), and shifting the step direction of the front legs so as to pull the body into the turn (Figure S1B). It should be emphasized that stride length modulations during steering are small, around 5% of the body length; nonetheless, they can produce fast body rotations because they are repeated at about 10 Hz and are deployed in combination. This overall description of leg movements during steering is largely consistent with previous work^37,38^.

Supporting our hypothesis, we found that different types of steering maneuvers were associated with systematically different leg gesture usage. We divided steering maneuvers into two types, pivots and swerves. We define a “pivot” as an epoch where the body rotates while slowing or reversing forward movement and a “‘swerve” as an epoch where the body rotates while increasing forward movement. Whereas a pivot tends to produce tight kinks in a walking path, a swerve produces a smooth curve (Figure 1D). We found that the first gesture (increasing stride length on the outside of a turn) was more prominent in swerves versus pivots, whereas the second gesture (decreasing stride length on the inside of a turn) was more prominent in pivots versus swerves (Figure 1E). These differences in stride length resulted from changes in the power stroke as well as changes in the return stroke (Figure S1C). We also found that the third gesture (shifting step direction) was more prominent in pivots versus swerves (Figure S1C).

In short, different steering maneuvers arise from different combinations of leg gestures. This suggests that leg gestures are elemental components of neural control — that is, they are among the fundamental building blocks used to generate a particular path through the environment. We wondered whether the brain can independently control the recruitment of different leg gestures. Therefore, we next set out to expand the list of identifiable DNs associated with steering control, with the ultimate goal of determining whether any are responsible for generating specific leg gestures.

### Many descending neuron types correlate with the body’s rotational velocity

We focused on DNs that are genetically accessible, meaning that there exists a specific transgenic driver line that can be used to target that DN for perturbation or recording^29^. We specifically examined those DN types that are anatomically positioned to influence at least two legs, meaning that they project to at least two homolateral (“same-side”) leg neuromeres in the ventral nerve cord. This pointed us toward 16 DN types. Here we use “DN type” to refer to a morphological category of DNs^29^. Most DN types innervating the leg neuromeres consist of a single right-left cell pair^29^, and this was true of all the DN types we examined.

In each DN type, we co-expressed the genetically encoded calcium indicator jGCaMP7s and the red fluorophore CyRFP1 (for motion correction), and we monitored neural activity as the fly walked on a spherical treadmill. We found that all but three of these DN types had increased activity during walking bouts, as compared to non-walking epochs (Figure S2). One of these (DNg34) also showed a graded relationship with forward velocity (Figures S2 and S3).

**Figure 2.**
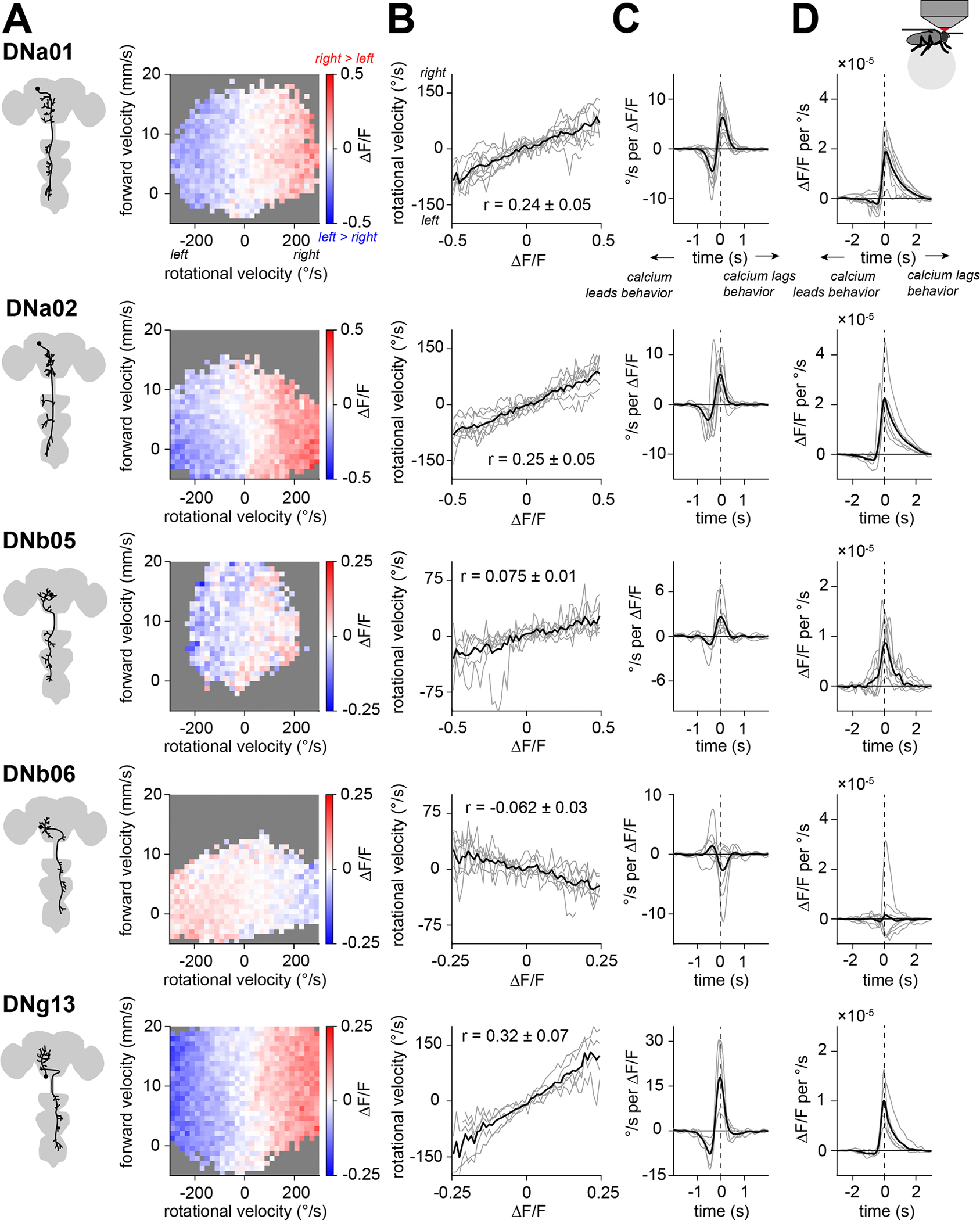
Many descending neuron types correlate with the body’s rotational velocity DNa01, DNa02, DNb05, DNb06, and DNg13 are represented by a single right-left cell pair; calcium imaging shows that all 5 correlate with rotational velocity. (A) *Left:* schematic of the left cell for each DN type. *Right:* in example flies, the mean right-left difference in ΔF/F, as a heatmap over rotational and forward velocity bins. Here and in (B), we excluded times when the fly was not walking. Gray bins had <20 time points. (B) Rotational velocity is related to the right-left difference in ΔF/F. Means of individual flies are in gray, with the mean across flies in black. The mean ± SEM across flies of the correlation between rotational velocity and ΔF/F is shown. For (B-D), n (flies) is 10 (DNa01), 8 (DNa02), 7 (DNb05), 7 (DNb06), 5 (DNg13). (C) Filters describing the mean rotational velocity around an impulse change in the right-left difference in ΔF/F. Here and in (D), we did not exclude times when the fly was not walking. (D) Filters describing the mean right-left difference in ΔF/F around an impulse change in rotational velocity. See also Figures S2 and S3.

We discovered that five DN types showed right-left activity differences that were consistently correlated with the body’s rotational velocity. Four of these five cell types were correlated with ipsiversive steering—i.e. the cell with a soma in the right hemisphere was more active during right turns and *vice versa* (DNa01, DNa02, DNb05, and DNg13; Figures 2A and S3). The fifth cell type was correlated with contraversive steering (DNb06; Figures 2A and S3). For all five DN types, we found that right-left differences in DN activity were linearly correlated with the body’s rotational velocity (Figure 2B).

If these DNs influence steering, their activity should precede changes in rotational velocity. To investigate this, we computed the linear filters relating rotational velocity to neural activity, where “neural activity” means the right-left difference in ΔF/F in a given DN type. We can compute a linear filter in two ways. A neuron→behavior filter describes the average relationship between a transient step of neural activity (a unit impulse) and a change in rotational velocity (Figure 2C); conversely, a behavior→neuron filter describes the average relationship between a unit impulse of behavior and a change in neural activity (Figure 2D). These filters account for the limited speed of calcium signals and the timescales of behavior. For both types of filters, we found that changes in ΔF/F preceded changes in behavior for all of the turning-correlated DNs (Figures 2C and 2D). Thus, these DNs could be causal for steering.

Our data point to five specific DN types whose activity precedes and predicts steering. With calcium imaging, these five cell types appear to carry redundant signals. However, it seems unlikely that all these DNs are merely carrying copies of the same command. Rather, we wondered whether different steering DNs might have specialized roles in fine-grained control of leg movements.

### Ipsiversive steering-correlated descending neurons DNa02 and DNg13 have distinct inputs and outputs

**Figure 3.**
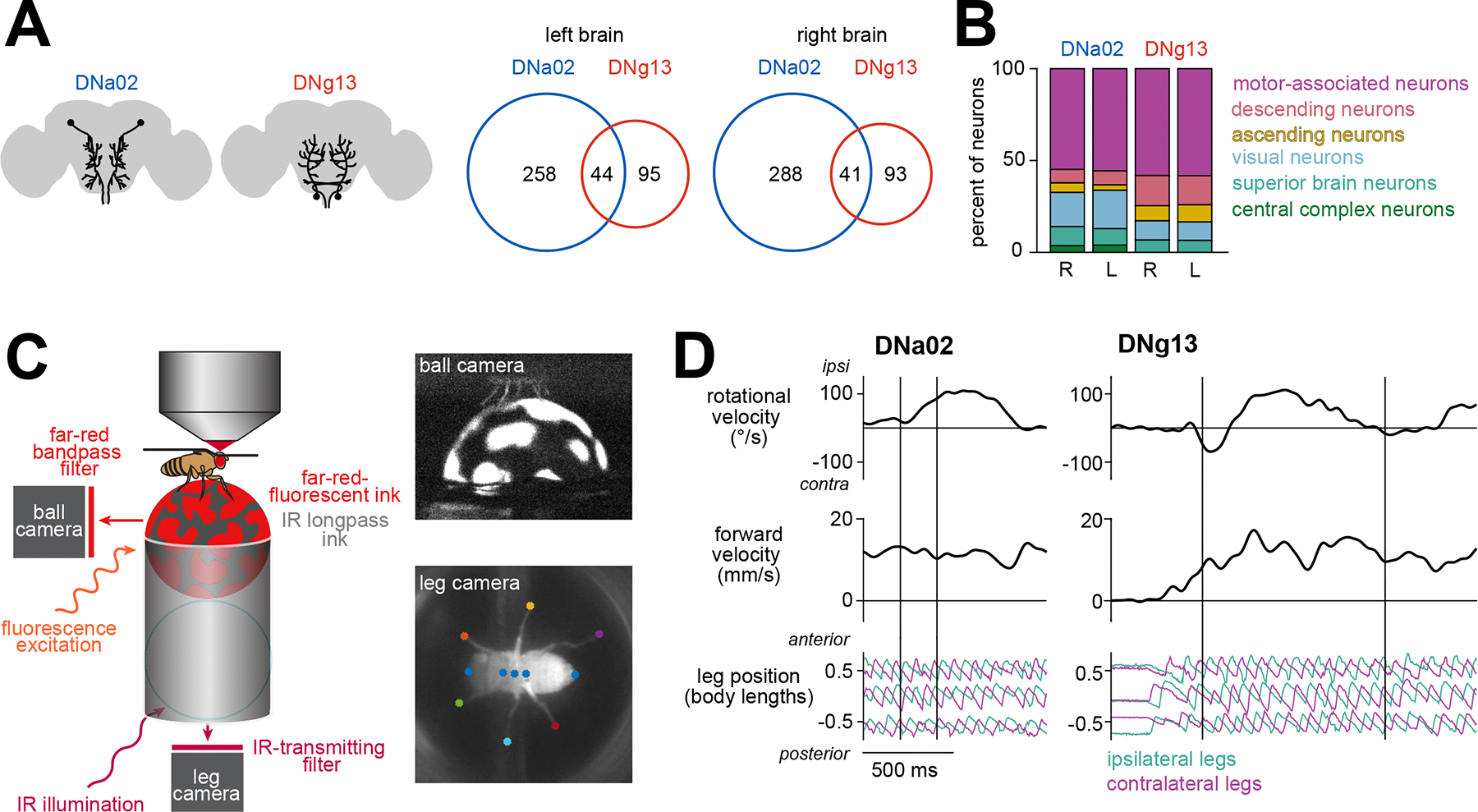
DNa02 and DNg13 have distinct inputs in the brain and distinct effects on leg movement (A) *Left:* Schematic of the left and right DNa02 and DNg13 cells in the brain. *Right*: The full brain connectome shows that DNa02 and DNg13 neurons in the same hemisphere share relatively few presynaptic cells (numbers are cell counts). (B) Summary of the cells presynaptic to each DN in the brain. These DNs also receive axo-axonic inputs in the ventral nerve cord, which are not included here. (C) The fly walks on a spherical treadmill coated with IR long-pass ink and painted with spots of far-red fluorescent ink. The camera used for tracking the sphere (and thus the fictive body velocity) captures only far-red light, making the ball appear black with white spots (*top*). The camera used for tracking the legs captures only IR light; because the sphere is transparent to IR light, it is not visible in this view (*bottom*). Key points tracked by Animal Part Tracker are marked. (D) Example trials for unilateral optogenetic stimulation of DNa02 with CsChrimson (*left*) and unilateral depolarization of DNg13 by current injection (*right*). Vertical lines mark the onset and offset of stimulation. See also Figure S4.

To test this hypothesis, we focused on DNg13 and DNa02, which had the strongest correlation with rotational velocity (Figure 2B). From the perspective of their soma and dendritic arbor, both DN types predicted ipsiversive steering. However, DNg13 axons cross the midline as they descend into the ventral nerve cord, whereas DNa02 axons do not. Furthermore, when we used the whole brain connectome^23,26^ to identify the direct inputs to DNa02 and DNg13, we found that these two cells receive input from largely non-overlapping individual presynaptic neurons (Figures 3A, S4A, and S4B). This finding implies that DNa02 and DNg13 can be recruited independently.

An interesting example of this are the projections from the central complex, the locus of spatial memory and navigation in the insect brain. DNa02 is directly targeted by central complex output neurons responsible for feedback control of heading^32,62–64^. By contrast, DNg13 receives no direct central complex output (Figures 3B and S4C).

Aside from the central complex, we did not find regions upstream from just one of these cells (Figures 3B and S4C). For example, both DNs are downstream of visual regions (the optic lobe and optic glomeruli). Moreover, both DNs are postsynaptic in motor-associated brain regions. Both DNs also receive substantial input from other DNs, an example of the many DN-DN connections in the brain^65^. Finally, both DNs receive input from ascending neurons. Thus, these DNs participate in similar categories of brain networks; they are simply targeted by different sets of presynaptic cells.

### Different descending neurons drive distinctive leg gestures

**Figure 4.**
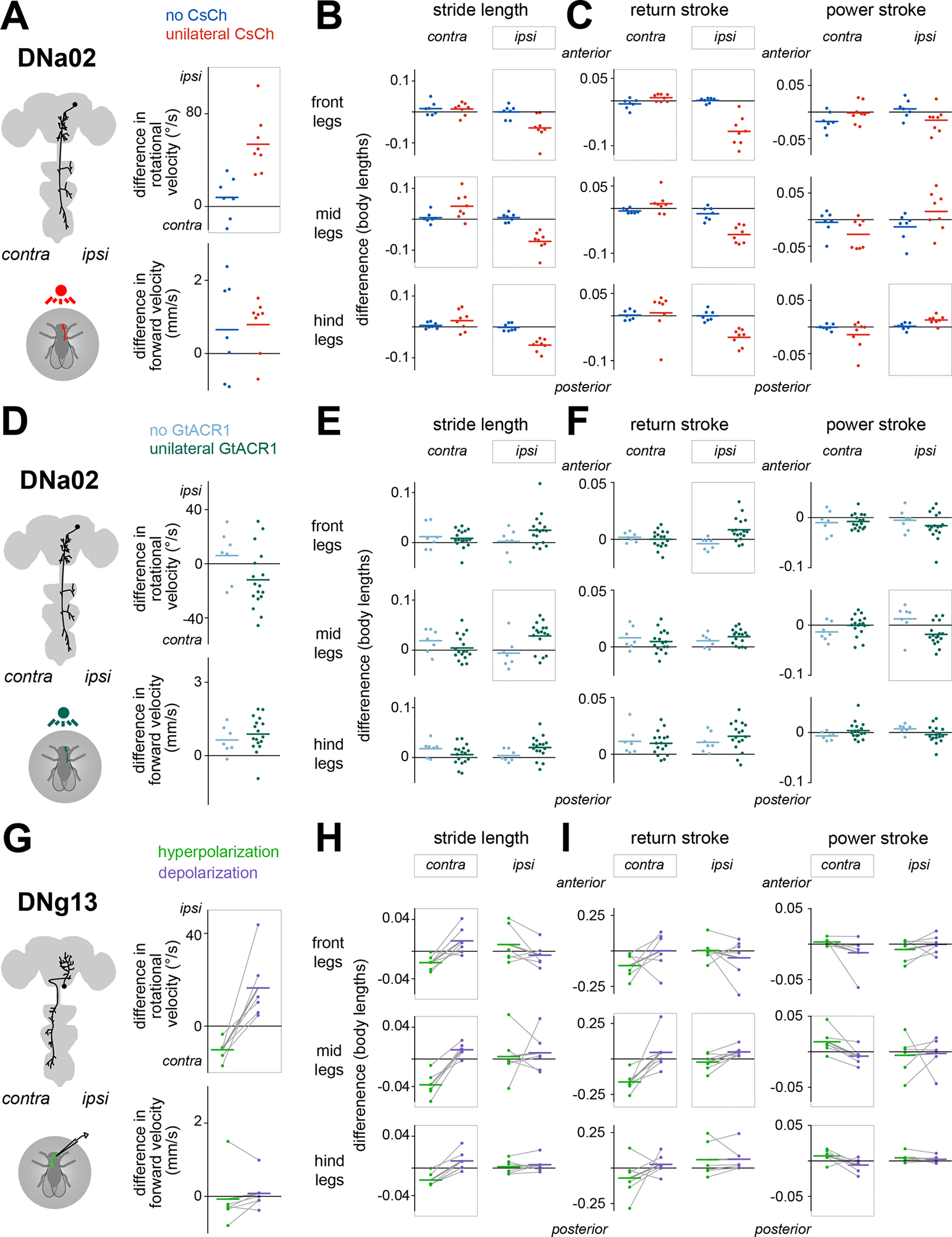
Different descending neurons drive distinctive leg gestures (A) Difference in rotational and forward velocity during unilateral stimulation of DNa02 with CsChrimson (CsCh). Dots are flies, and lines are means. Ipsiversive (positive) and contraversive (negative) are relative to the side of the soma of the CsCh+ cell and randomly assigned for no CsCh controls. Differences are significant for rotational but not forward.n=8 (CsCh+) and 7 (no CsCh) flies. Box marks a significant change. (B) Unilateral stimulation of DNa02 produces leg-specific changes in stride length. Boxes around ipsi/contra mark significant effects ±CsCh in post-hoc tests considering all three legs on one side, while boxes around individual panels mark significant effects for that particular leg. (C) Unilateral stimulation of DNa02 shortens the return stroke. (D) Difference in rotational and forward velocity during unilateral hyperpolarization of DNa02 with GtACR1. n=17 (GtACR1+) and 7 (no GtACR1) flies. (E) Unilateral hyperpolarization of DNa02 increases ipsilateral stride length. (F) Unilateral hyperpolarization of DNa02 lengthens the return and power strokes. (G) The effects of DNg13 current injection are significant for rotational but not forward velocity. n=6 flies. Lines connect data from the same fly. (H) DNg13 current injection produces leg-specific changes in stride length. (I) DNg13 current injection affects stride length during both phases of the stride cycle in the contralateral legs. See also Figures S5 and S6. See Table S1 for statistics.

To determine how each DN influences steering, we perturbed neural activity unilaterally while simultaneously monitoring the positions of all six legs and the body’s fictive velocity (Figures 3C and 3D). To unilaterally stimulate DNa02, we expressed the light-gated cation channel CsChrimson in individual cells using a genetic mosaic strategy^36^, and we confirmed that brief pulses of light (200 ms) reliably drove spiking (Figure S5A). We compared the behavioral responses of experimental flies (having expression in one copy of DNa02) with control flies (having expression in neither copy of DNa02); note that these flies are genetically identical and have the same average pattern of expression in “off target” cells. In experimental flies, we found that a pulse of light triggered an ipsilateral turn, with no change in forward velocity as compared with controls (Figure 4A). Bilateral activation of DNa02 instead triggered a decrease in forward velocity without a change in the heading direction (Figure S6A).

Examining leg movements, we found that unilateral DNa02 stimulation decreased the stride length of all three ipsilateral legs, with less consistent effects on the contralateral legs (Figure 4B). This makes sense, given the anatomy of this DN. The effect of DNa02 on stride length was primarily attributable to changes in the return stroke, while the power stroke was largely unaffected (Figure 4C). Bilateral stimulation produced the same leg movement changes, except bilaterally instead of unilaterally (Figures S6B and S6C).

To unilaterally hyperpolarize DNa02, we used our genetic mosaic strategy to express the light-gated chloride channel GtACR1 (Figure S5A). In response to pulses of light, experimental flies expressing GtACR1 in one copy of DNa02 showed a trend toward contraversive turns, although this fell short of statistical significance (Figure 4D). There was no change in forward velocity as compared with controls (Figure 4D). Examining the underlying leg movements, we found that ipsilateral stride length increased (Figure 4E), and this was primarily attributable to changes in the return stroke (Figure 4F). Thus, unilateral hyperpolarization and depolarization of DNa02 produce opposing effects on leg movements. Bilateral hyperpolarization produced no significant changes in either body velocity or leg movements (Figures S6D–F).

Next, we stimulated DNg13 cells. We could not use our genetic mosaic strategy to produce specific unilateral stimulation of this cell type (see Methods), so we instead targeted DNg13 via whole-cell patch-clamp recordings. We injected pulses (1 s) of depolarizing or hyperpolarizing current, thereby increasing or decreasing the cell’s firing rate (Figure S5B). Depolarizing current triggered ipsilateral turning (relative to the recorded soma), whereas hyperpolarizing current triggered contralateral turning, with no changes in forward velocity (Figure 4G).

Examining leg movements, we found that DNg13 perturbations mainly affected the legs contralateral to the stimulated cell, again consistent with the cell’s anatomy. In particular, contralateral strides were significantly lengthened during depolarization as compared with hyperpolarization; this was not true of ipsilateral strides (Figure 4H). This effect was a consequence of changes in the power stroke as well as the return stroke (Figure 4I). DNg13 drives the leg farther backward during the power stroke and farther forward during the return stroke. These results provide evidence that a single DN can have opposite effects on leg movement during different phases of the locomotor cycle.

To summarize, each of these DNs generates specific leg gestures associated with steering. DNa02 shortens steps on the inside of a turn, while DNg13 lengthens steps on the outside of a turn. The lateralized specializations of these DNs are related to their anatomy: both cells drive ipsiversive rotations, but DNa02 projects ipsilaterally, whereas DNg13 projects contralaterally.

Finally, it is worth remembering that a unilateral projection to the ventral nerve cord can influence both sides of the body, via local connections that coordinate leg movements across the midline. Indeed, we observed that unilateral activation of either DNa02 or DNg13 shifted the step direction of both front and middle legs so as to pull the body into the turn, while unilateral inhibition shifted it in the opposite direction (Figure S6G). In short, each DN produces a coordinated set of leg kinematic changes, comprising homolateral stride length changes and bilateral, leg-specific step-direction changes.

### Descending neuron activity is correlated with specific leg gestures

**Figure 5.**
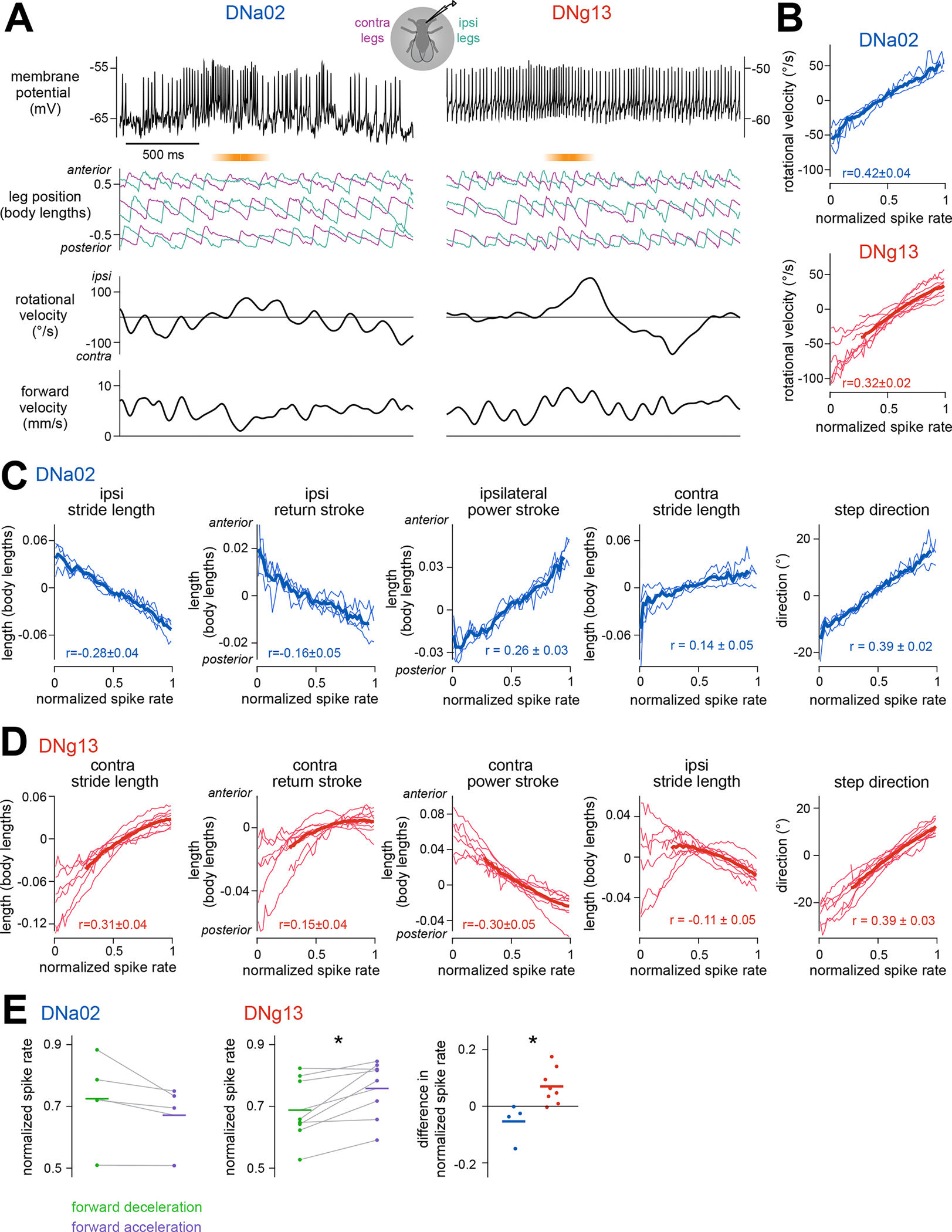
Descending neuron activity is correlated with specific leg gestures (A) Example recordings. DNa02 spiking increases just before a decrease in ipsilateral stride length and ipsiversive turning. DNg13 spiking increases just before an increase in contralateral stride length and ipsiversive turning. Shading highlights the change in behavior. (B) Rotational velocity is related to DNa02 and DNg13 spike rate (normalized per cell). Positive rotational velocity is ipsiversive. Thin lines are mean values for individual flies; thick lines are averages across flies (DNa02: n=4 cells in 4 flies; DNg13: n=9 cells in 9 flies). Mean ± SEM across flies of the correlation between rotational velocity and spike rate is also shown. (C) Same but relating DNa02 spike rate with ipsilateral (i.e. the side affected by optogenetic manipulation) step parameters, contralateral stride length, and step direction. (D) Same but for DNg13 and contralateral (i.e. the side affected by current injection) step parameters, ipsilateral stride length, and step direction. (E) *Left*: DNa02 and DNg13 spike rate when steering ipsilaterally while also decelerating or accelerating in the forward direction. Dots are means for each fly, lines are means across flies, and gray lines connect points from the same fly. *Right*: difference in spike rate for acceleration - deceleration. Asterisks mark significant differences. See also Figure S7. See Table S1 for statistics.

If DNa02 and DNg13 control specific leg gestures, then we would expect that their activity correlates with and precedes those leg gestures. To test this, we performed whole-cell patch-clamp recordings from these DNs in walking flies (Figure 5A). Compared with calcium imaging, this approach is a better match to the fast timescale of leg movements. During each recording, we monitored the positions of all six legs and the body’s fictive velocity (Figure 3C). The firing rate of both DNs was higher when the fly was walking (Figure S7A), and it was positively correlated with the body’s ipsiversive rotational velocity (Figure 5B).

Analyzing leg movements, we found that DNa02 spike rate was inversely related to ipsilateral stride length, including the ipsilateral return stroke (Figure 5C). This aligns with our finding that direct DNa02 activation shortens ipsilateral stride length mainly through shortening the ipsilateral return stroke and that DNa02 hyperpolarization increases ipsilateral stride length (Figures 4B, 4C, 4E). Together, these results indicate DNa02 is normally recruited to produce these modulations in leg movement.

Meanwhile, DNg13 spike rate was positively related to contralateral stride length, including both the power stroke and the return stroke of the contralateral legs (Figure 5D). This aligns with our finding that DNg13 activation increases contralateral stride length and inhibition decreases it, with effects on both the power stroke and the return stroke (Figures 4H and 4I). Together, these results indicate DNg13 is normally recruited to produce these modulations.

Additionally, DNa02 and DNg13 were correlated with leg kinematic changes that were not observed after direct stimulation of these DNs (Figures 5C and 5D). This is not surprising because many leg kinematic features are themselves correlated (Figure S7B). Thus, correlation is not strong evidence of causation in this system. However, a failure to find a correlation would be evidence against causation, which is why it is important to check that DN spiking actually correlates with the expected leg kinematic changes.

Finally, we asked whether the spike rate of these DNs was differentially correlated with steering that increased versus decreased the fly’s forward velocity. Indeed, during steering in the cell’s preferred direction, DNa02 spike rate tended to be higher when the forward velocity was decreasing while DNg13 spike rate tended to be higher when the forward velocity was increasing (Figure 5E). This suggests that these DNs might be differentially recruited during different steering maneuvers.

### DNa02 and DNg13 are not routinely recruited as speed-control DNs

Thus far, we have focused on asymmetric changes in stride length, which rotate the body; however, symmetric changes in stride length change the body’s forward velocity^38,66,67^. Indeed, bilateral activation of DNa02 reduced forward velocity (Figures S7A–C). However, we do not think that DNa02 (or DNg13) is routinely recruited for forward speed control.

First, DNa02 spike rate is not correlated with forward velocity during straight walking, and DNg13 spike rate is only weakly so (Figures S7C and S7D). Moreover, in our calcium imaging experiments, the total bilateral activity in DNa02 and DNg13 was not correlated with forward velocity (Figures S2B and S2E). Furthermore, during straight walking, the spiking of both DNs was only weakly predictive of stride length, as compared with during turning (Figures S7C and S7D). Finally, normal forward speed modulations involve lateral shifts of the legs^66^, but our DNa02 and DNg13 manipulations produced lateral shifts in the wrong direction (Figures S7E and S7F). These observations together imply that neither DNa02 nor DNg13 are routinely recruited as forward speed-control DNs, although they may participate in speed control in specific contexts.

### Different descending neurons are recruited at different moments within a steering maneuver

**Figure 6.**
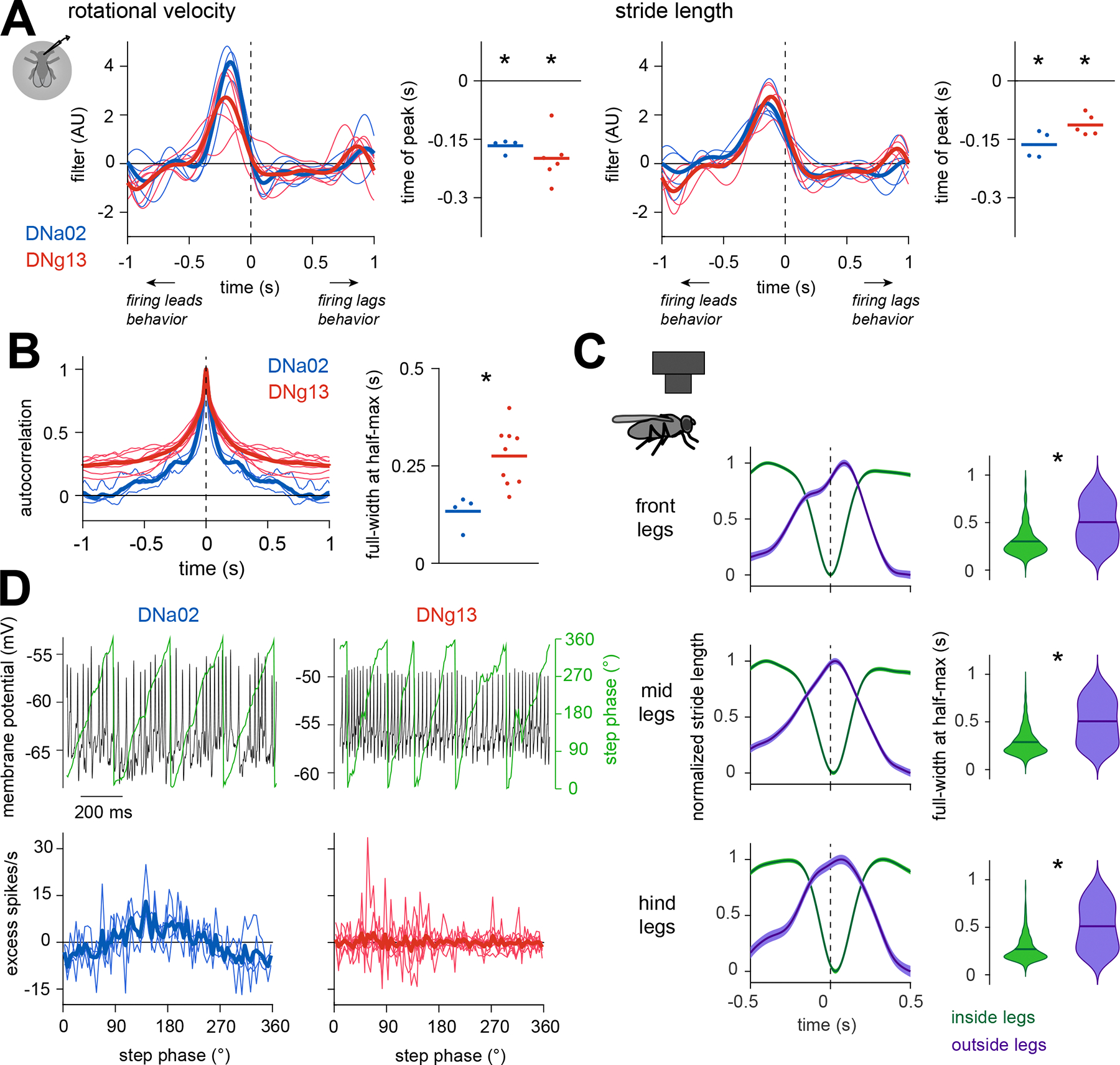
Different descending neurons are recruited at different moments within a steering maneuver (A) *Left*: Filters show mean change in rotational velocity around an impulse change in DN firing rate. Thin lines are individual flies, thick lines are means across flies (DNa02: n=4 cells in 4 flies; DNg13: 5 cells in 5 flies). Stripchart shows time of the filter peak; dots are individual flies, horizontal lines are means across flies. Asterisks mark significant differences. *Right*: Filters show mean change in stride length around an impulse change in DN firing rate (in ipsilateral legs for DNa02 and contralateral legs for DNg13). For ease of comparison, the filter for DNa02 was multiplied by −1. Stripchart shows time of the filter peak. (B) Autocorrelation of DNa02 and DNg13 spike rate during walking (DNa02: n=4 cells in 4 flies; DNg13: 9 cells in 9 flies). Stripchart shows the full-width at half-maximum of the autocorrelation function. (C) *Left:* Changes in stride length over time for each pair of legs during turning bouts in freely walking flies. Bouts are aligned to the peak in rotational velocity (n=818 bouts in 73 flies). *Right*: The full width at half-maximum for the change in stride length over time.. Violin plots are Gaussian kernel estimates fit to all data points. (D) *Top*: example recordings of DNa02 and DNg13, overlaid with the stride cycle phase. The stride cycle is expressed relative to the phase of the ipsilateral (DNa02)/contralateral (DNg13) middle leg. *Bottom:* Timing of DNa02 and DNg13 spikes relative to the stride cycle 100 ms prior. DNa02: n=4 cells in 4 flies; DNg13: 9 cells in 9 flies. See Table S1 for statistics.

Next, we compared firing rate dynamics with behavioral dynamics. On average, firing rate changes in these DNs preceded changes in rotational velocity and stride length by about 150 ms (Figure 6A), consistent with the hypothesis that these DNs actually influence these behaviors. The latency of the behavioral response may be partly because of neural and muscular delays and partly because of the inertia of the spherical treadmill.

Interestingly, DNg13 firing rate modulations were slower than DNa02 firing rate modulations, as evidenced by the different widths of each cell’s autocorrelation function (Figure 6B). This difference is notable because it mirrors the way that ipsilateral and contralateral legs are coordinated during steering maneuvers. Specifically, during a turning bout, stride length on the outside of the turn tends to slowly increase and decrease, while stride length on the inside of the turn changes more abruptly and transiently (Figure 6C). Our data suggest that the timing of this pattern originates in the brain, at the level of DN dynamics.

We wondered whether some of the fine structure in DNa02 and DNg13 spiking was locked to the stride cycle. We therefore extracted the phase of the stride cycle at every time point, and we binned all DN spikes according to this phase. DNa02 spikes occurred preferentially at a specific phase of the stride cycle, whereas DNg13 spikes did not (Figure 6D). Although this stride-locked firing rate modulation was only about 15 spikes/sec (approximately 10% of the cell’s dynamic range), it was highly consistent across DNa02 recordings. Stride-locked modulation in DNa02 could arise from the ascending projections that this cell receives from the ventral nerve cord^68,69^, since the locomotor rhythm originates in the cord^3–7^.

### Descending neuron connectivity implies dynamic gating in the ventral nerve cord

**Figure 7.**
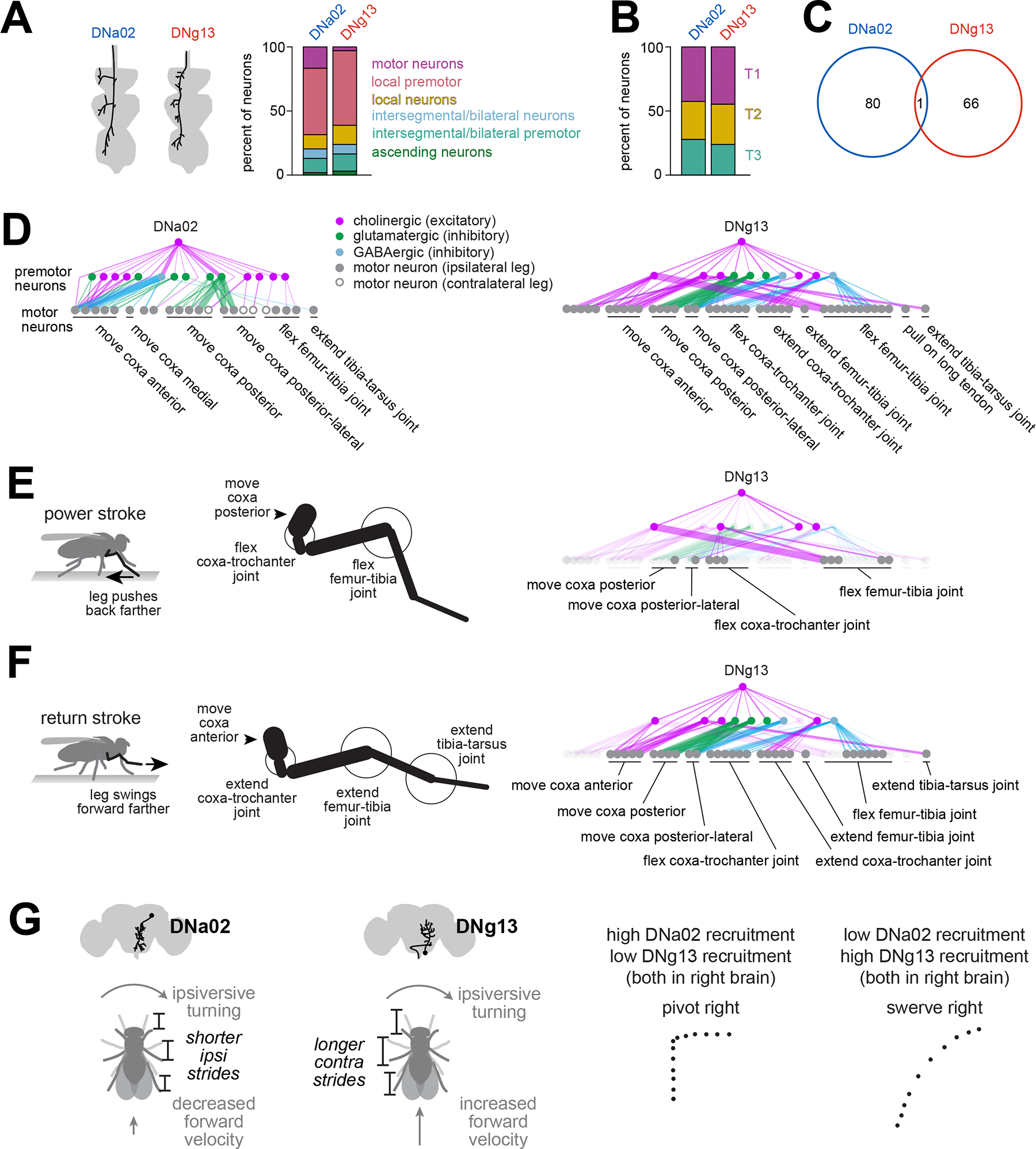
Steering descending neurons have specialized outputs (A) Schematic: morphology of the left DNa02 and the right DNg13 axons in the ventral nerve cord. Here, left and right refer to soma locations in the brain; both of these cells send axons to the left side of the cord. Stacked bar charts summarize the cells postsynaptic to each DN in the leg neuromeres of the cord. Note that DNa02 also targets neurons in the neuropils associated with the wings, halteres, and neck, and those are excluded from this plot. (B) The distribution across the three leg neuromeres (T1, T2, T3) of the postsynaptic neurons. Intersegmental neurons are assigned based on their cell body locations. (C) DNa02 and DNg13 axons projecting to the left side of the cord share almost no postsynaptic cells (numbers are cell counts). (D) Monosynaptic and disynaptic connections between DNa02/DNg13 axons projecting to the left side of the cord and motor neurons controlling the front legs. DNa02 and DNg13 graphs are plotted separately, and thus some motor neurons are repeated. Motor neurons are labeled according to their inferred effect based on published anatomical work^27^, with unlabeled motor neurons having unknown effects. Line thickness is proportional to the number of synapses. Connections between premotor neurons are not shown. (E) *Left*: limb kinematics during the power stroke. *Right*: cells downstream from DNg13 that can potentially explain its ability to increase stride length during the power stroke. (F) Same but for return stroke. (G) Schematic summarizing the effects of each DN type on stride length, body velocity, and path shape. See also Figure S4.

Finally, we used the ventral nerve cord connectome^25,27^ to ask whether the inferred function of each DN is compatible with its synaptic targets in the cord (Figure S4D). Consistent with a recent analysis^70^, we found that both DNa02 and DNg13 synapse mainly onto premotor neurons–i.e. neurons presynaptic to motor neurons (Figures 7A and S4E); the DNs also make a few direct connections onto motor neurons. Each DN distributes its outputs to all three pairs of legs (Figures 7B and S4F) but with essentially no overlap between the two DNs (Figures 7C and S4G).

To better understand the logic of these connections, we focused on the region of the ventral nerve cord that controls the front legs — the T1 neuromeres — because T1 motor neurons have been mapped to their muscle targets and actions on the leg joints^27^. We identified all the monosynaptic and disynaptic outputs of DNa02 and DNg13 and their likely neurotransmitters^71,72^. DNa02 and DNg13 are upstream from many motor neurons that collectively control muscles throughout the leg (Figure 7D). Specifically, DNg13 is positioned to increase the leg’s displacement during the power stroke and the return stroke, consistent with our functional data. During the power stroke, DNg13 likely excites motor neurons that move the coxa posterior, motor neurons that flex the femur-tibia joint, and motor neurons that flex the coxa-trochanter joint (Figures 7D and 7E). These should all lengthen the power stroke. Then, during the return stroke, DNg13 is positioned to excite motor neurons that move the coxa anterior, motor neurons that extend the coxa-trochanter joint, motor neurons that extend the femur-tibia joint, and motor neurons that extend the tibia-tarsus joint, while also inhibiting motor neurons that move the coxa posterior, motor neurons that flex the coxa-trochanter joint, and motor neurons that flex the femur-tibia joint (Figures 7D and 7F). These should all lengthen the return stroke. In short, connectome data are consistent with our finding that DNg13 increases stride length by modulating both phases of the step cycle. Importantly, however, this anatomy is only compatible with our results if we assume that incoming descending signals are dynamically gated by the locomotor cycle in the ventral nerve cord.

The situation for DNa02 is more complicated. During the return stroke, DNa02 is positioned to strongly inhibit motor neurons that move the coxa anterior (Figure 7D), which could shorten the return stroke, consistent with our functional data. That said, DNa02 is also positioned to excite these same motor neurons; it is also positioned to inhibit the motor neurons that move the coxa posterior (Figure 7D). Knowing how these signals are gated by the locomotor rhythm should help clarify the logic of this connection pattern.

As an aside, we were surprised to discover that some cells postsynaptic to DNa02 were wing, haltere, or neck motor neurons or premotor neurons (Figures S6H and S6I; also reported in^70^). This was not the case for DNg13, whose direct postsynaptic neurons were all related to leg control (Figure S6H). This connectivity suggests that DNa02 contributes to steering during flight as well as walking, whereas DNg13 is specifically involved in steering during walking.

## Discussion

### Elemental components of locomotor control

The components of motor control at the level of the spinal cord or ventral nerve cord are not necessarily the same as the components of motor control from the brain’s perspective. In particular, during locomotion, the cord is responsible for generating the cyclical patterns of muscle activation that produce alternating power and return strokes, via recruitment of muscle synergies^22^. Meanwhile, the brain is thought to control higher-level features of locomotion, such as heading direction. But there are also more granular components of locomotion that the brain might have access to, such as coordinated changes in specific combinations of limbs (gestures). To understand the descending control of locomotion, we need to identify what sorts of components are relevant to the brain.

Here we focused on the elements of steering control in walking *Drosophila*. We found that different steering maneuvers involve different combinations of leg gestures. Specifically, one gesture (increasing stride length on the outside of a turn) is more prominent in swerves versus pivots, whereas another gesture (decreasing stride length on the inside of a turn) is more prominent in pivots versus swerves. DNg13 can evoke the first gesture, while DNa02 can evoke the second gesture. Moreover, DNg13 and DNa02 are downstream from largely non-overlapping networks in the brain, implying that these DNs can be recruited independently. We propose that, by varying the ratio of activity in these two DNs, the brain can control the generation of pivots versus swerves (Figure 7G). Here we have focused on the simplest possible taxonomy of steering maneuvers, involving just two types of turns; in reality, there is a graded spectrum of steering maneuvers during walking^58^, which could arise from graded variations in the recruitment of DNg13 and DNa02 and almost certainly other steering DNs as well^31,32,35,49,58^.

Strikingly, the function of DNa02 resembles that of a DN type in the gigantocellular (Gi) reticular nucleus of the mammalian brainstem that expresses Chx10. *Chx10* Gi cells are active during ipsiversive turns^73^, and unilateral activation reduces stride length on the ipsilateral side of the body, resulting in an ipsiversive turn with a transient decrease in forward velocity^20,74^. Bilateral activation of *Chx10* Gi cells can arrest locomotion^75^, and bilateral activation of DNa02 slows forward walking. In *Drosophila*, DNa02 plays a role analogous to that of *Chx10* Gi cells, but DNa02 also exists in parallel with another descending cell type that lengthens strides contralaterally (DNg13). Together, these two descending pathways can explain why it is possible for forward velocity to either increase or decrease during a turn (Figure 7G).

Our results support a model where the speed and direction of locomotion are each influenced by the activity of multiple DNs, with some DNs contributing to both^30–33,35,49,57^. Both parameters are also under feedback control, as sensory reafference continuously informs the brain about the body’s instantaneous forward velocity^76–78^ and heading^79^. Our results help illustrate how these control loops can recruit specific DNs. For example, DNa02 is a major target of the central complex^32,62^, the brain region that compares the animal’s current heading to an internal goal direction^63,64,79,80^. When the central complex commands a large change in heading, it also commands a decrease in forward velocity^63,80^ — i.e., pivoting — which is associated with the leg gestures that DNa02 controls. Together, these results suggest that the brain’s system for monitoring and controlling head direction is tightly coupled to the DNs that control pivoting.

Conversely, our data imply that DNg13 changes heading without decreasing forward velocity. Interestingly, this DN is not a direct target of the central complex; rather, it is downstream from other networks, including visuomotor circuits. This cell may be recruited by brain networks that stabilize straight walking, correcting for small, involuntary body rotations while continuing the body’s forward progression.

In the future, we expect that the *Drosophila* connectome will help us understand the functions of other DN types involved in steering^31,32,53,57^. A recent connectome study revealed that there are just over 650 pairs of DNs^70^. Approximately 20% of these project unilaterally and synapse in all three homolateral leg neuromeres, like the five steering DNs in this study; some of these may represent additional steering DNs. These other DNs may control different steering-related leg movements^32,37,38,81^ and body movements^58,82,83^. Additionally, some DNs may only be recruited under specific internal states: for example, DNp09 has been suggested to control male steering during courtship^35^; this cell type was not active in our experiments, which focused on females, consistent with previous work^35^. A full connectome of the central nervous system will help us understand patterns in DN organization and make predictions about DN function.

### Rhythmic activity in descending neurons

Locomotor rhythms are prominent in the vertebrate brainstem^84–87^, cortex^88^, and cerebellum^89^. These rhythms are generally attributed to ascending projections from the spinal cord^9^, but vestibular signals may also contribute^90^. Interestingly, locomotor rhythms are prominent in DNs that drive steering^87,91,92^, and it has been suggested that these signals produce laterally asymmetric modulation of muscle contractions at appropriate times in the locomotor cycle^93^. However, we do not yet have a detailed understanding of how these DNs orchestrate steering, and so the function of their rhythmicity is still uncertain.

Here we show that DNa02 is also phase-locked to the locomotor cycle, likely because of ascending signals^68,69^. Indeed, DNa02 receives substantial monosynaptic input from ascending neurons. Notably, although DNg13 also receives input from ascending neurons, these are not shared with DNa02, potentially explaining its lack of phase-locked activity. A similar walking rhythm has also been observed in the visual system, and future work will be needed to determine whether it has a common origin with the rhythm in DNa02^94^.

Our results suggest that DNa02 drives different motor neurons at different phases of the step cycle. Because a given step-cycle phase occurs at a different time in the front/back legs versus the middle legs^37,38,95^, we can infer that DNa02 probably has asynchronous effects on analogous motor neurons in different legs. It seems that, rather than sending a differently phase-shifted signal to different legs, the brain sends a single rhythmic signal to all the homolateral legs, and then relies on local mechanisms, such as proprioceptive feedback or the dynamics of the premotor circuits, to time-shift the effect of this signal.

### Opposing effects of single descending neurons

In vertebrates, stimulation of specific brainstem loci can evoke opposing effects on limb kinematics, depending on the phase of the locomotor rhythm when the stimulus arrives^17,96^. There are two possible explanations for this phenomenon. First, the same DN could have opposing effects on spinal targets, depending on the phase of the locomotor rhythm. Alternatively, the stimulus might recruit DNs with opposing effects, which are then gated at the level of the spinal cord.

Here we show that, in *Drosophila*, a single DN can in fact have opposing effects, depending on the phase of the locomotor cycle. Specifically, our perturbation experiments imply that DNg13 drives the legs to a more posterior position during the power stroke and to a more anterior position during the return stroke. Thus, it drives increased flexion in one phase, and increased extension in another phase. (Whether DNa02 activity can also have phase-specific effects is less clear, given that this DN’s effects were primarily but not strictly limited to the return stroke, and different methods were used to manipulate these cells.)

DNg13 connectivity in the ventral nerve cord provides a mechanism to explain this phenomenon. DNg13 is positioned to flex and extend multiple leg joints via distinct interposed interneurons. It seems likely that these interposed interneurons are rhythmically locked to the step cycle, so that descending drive is re-routed to these effector pathways in an alternating manner, increasing the stride length in both phases of the step cycle.

### Beyond arthropods

There is good reason to think that locomotor control in arthropods is relevant to vertebrates. Arthropods and vertebrates share the same basic walking rhythm and can control the length, duration, and phase of their steps^2,97^. In both taxa, motor neuron recruitment follows the same basic relationship between force production and recruitment order^98^. Moreover, arthropods and vertebrates confront shared physical problems, such as the need to balance stability and maneuverability^99,100^. They also confront common cognitive problems in complex locomotor control: navigation toward faraway destinations, memorized locations, or remembered objects^101,102^.

Our results provide further support for the idea of deep homology between locomotor control systems in arthropods and vertebrates. The functional similarity of DNa02 cells (in *Drosophila*) and *Chx10* Gi cells (in mammals^20,74^) is perhaps the most notable example of this. If we can understand the logic of descending locomotor control in any species, it will allow us to re-frame the problems of navigation and locomotor planning as concrete problems of generating specific dynamic activity patterns in particular brain cells^103,104^.

### Limitations of the study

While we presented several indirect lines of evidence that DNa02 and DNg13 are recruited differentially during turning, we were unable to record from the two simultaneously to examine this directly. Furthermore, we studied steering in one behavioral context with limited sensory stimuli; in other contexts, other steering gestures may predominate. Future studies will be required to map the full range of leg gestures that flies use to turn and to understand how the DN population controls these gestures across contexts.

## STAR Methods

### Resource availability

#### Lead contact

Further information and requests for resources and reagents should be directed to and will be fulfilled by the lead contact, Rachel Wilson (rachel_wilson@hms.harvard.edu).

#### Materials availability

All reagents generated in this study are freely available upon request.

#### Data and code availability

- All data reported in this paper will be shared by the lead contact upon request.
- All original code has been deposited at Zenodo and is publicly available as of the date of publication. DOIs are listed in the key resources table.
- Any additional information required to reanalyze the data reported in this paper is available from the lead contact upon request.

### Experimental model and study participant details

#### Drosophila melanogaster

The isoD1 strain of *D. melanogaster* was used as wild-type^105^. All transgenes, strains, and mutant alleles used in this study are listed in the key resources table. Split-GAL4 line expression patterns are available at https://splitgal4.janelia.org/. GMR line expression patterns are available at https://flweb.janelia.org/. VT line expression patterns are available at virtualflybrain.org. All experimental flies were female. All flies except those used for optogenetic stimulation experiments were raised on standard cornmeal-molasses food (recipe B2, Archon Scientific) at 25 °C, 50–70% humidity, and a 12:12 light:dark cycle. Flies used for optogenetic experiments were raised at 25 °C and 50–70% humidity on semi-defined food^116^, following the BDSC protocol, supplemented with all-trans retinal (ATR, Sigma-Aldrich) at a final concentration of 0.6 mM. Because ATR is light-sensitive, vials were individually wrapped in foil, and the flies were raised in constant darkness.

For freely walking fly behavior experiments, female flies were collected on CO_2_ and housed in groups of 10–20 on cornmeal-molasses food until they were used for the experiments at 2–5 days post-eclosion. On the day of the experiment, individual vials were loaded into the automated dispenser. Experiments were performed during the flies’ subjective morning (within ~3 hours of the dark to light transition, Zeitgeber time 0).

For tethered fly experiments, female flies were anesthetized on ice for collection within 24 hours of eclosion. For calcium imaging experiments, most genotypes were used 1–3 days post-eclosion, but DNp05 flies were used 10–16 hours post-eclosion as the expression of jGCaMP7s was higher in younger flies. Flies were wet-starved by housing them in a vial with a water-dampened kimwipe but no food for 20–30 hours prior to the experiment to encourage walking. For flies used at 1 day or less post-eclosion, this meant that they were starved from collection until experiment. For flies used at older ages, they were housed on cornmeal-molasses food upon collection until the starvation period. Experiments were performed during the flies’ subjective evening (± 3 hours from the light to dark transition, Zeitgeber time 12). For optogenetic experiments, flies were collected onto semi-defined food with 0.6 mM ATR and housed in constant darkness. They were used for experiments at 7 days post-eclosion to allow time for the SPARC reaction to occur and CsChrimson or GtACR1 to be expressed. For the 20–30 hours prior to the experiment, these flies were wet-starved by housing them in a vial with a kimwipe dampened with water and 1 mM ATR. For electrophysiology experiments, flies were used 1 day post-eclosion. Like the calcium imaging flies, they were wet-starved for 20–30 hours post-eclosion, and experiments were performed in the flies’ subjective evening.

### Method details

#### Generating transgenic flies

The *UAS-SPARC2-D-GtACR1::eYFP* plasmid was generated by *de novo* synthesis (performed by Genscript) of *Drosophila* codon-optimized *GtACR1::eYFP* (sequence obtained from Genbank: KY385291.1^117^) and *SV40* (sequence obtained from *pJFRC7*, Addgene #26220^108^) into *pHD-SPARC2-D-LexA::p65* (Addgene #133562^36^), swapping out the existing effector and terminator. The transgenic flies were then generated by standard construct injection of the *UAS-SPARC2-D-GtACR1::eYFP* plasmid and the *pCFD5-U6-3-t-attP40* guide RNA plasmid (Addgene #133561^36^) and CRISPR-HDR (carried out by BestGene). Transformants were identified by expression of *3XP3-DsRed*. Before using the transgenic line in experiments, the *3XP3-DsRed* was excised by crossing with *CyO, P{w[*^+^*mC]=Crew DH1* flies (BDSC 1092), as it was flanked by *loxP* sites.

#### Fly genotypes by figure

Figures 1, S1, and 6C:

+; +; + isoD1 strain

Figure 2:

**DNa01**: *w[1118]/+; R22C05AD/UAS-CyRFP1; R56G08DBD/UAS-jGCaMP7s*

**DNa02:** *w[1118]/+; R75C10AD/UAS-CyRFP1; R87D07DBD/UAS-jGCaMP7s*

**DNb05:** *w[1118]/+; VT019391AD/UAS-CyRFP1; VT028198DBD/UAS-jGCaMP7s*

**DNb06:** *w[1118]/+; R20C04AD/UAS-CyRFP1; VT025999DBD/UAS-jGCaMP7s*

**DNg13:** *w[1118]/+; VT027166AD/UAS-CyRFP1; VT009857DBD/UAS-jGCaMP7s*

Figure S2:

**DNa01**: *w[1118]/+; R22C05AD/UAS-CyRFP1; R56G08DBD/UAS-jGCaMP7s*

**DNa02:** *w[1118]/+; R75C10AD/UAS-CyRFP1; R87D07DBD/UAS-jGCaMP7s*

**DNb05:** *w[1118]/+; VT019391AD/UAS-CyRFP1; VT028198DBD/UAS-jGCaMP7s*

**DNb06:** *w[1118]/+; R20C04AD/UAS-CyRFP1; VT025999DBD/UAS-jGCaMP7s*

**DNg13:** *w[1118]/+; VT027166AD/UAS-CyRFP1; VT009857DBD/UAS-jGCaMP7s*

**DNg14:** *w[1118]/+; R38H06AD/UAS-CyRFP1; VT018689DBD/UAS-jGCaMP7s*

**DNg15:** *w[1118]/+; VT043400AD/UAS-CyRFP1; VT043662DBD/UAS-jGCaMP7s*

**DNg16:** *w[1118]/+; UAS-CyRFP1/+; VT028153-GAL4/UAS-jGCaMP7s*

**DNg31:** *w[1118]/+; UAS-CyRFP1/+; GMR55D12-GAL4/UAS-jGCaMP7s*

**DNg34:** *w[1118]/+; UAS-CyRFP1/+; GMR83A12-GAL4/UAS-jGCaMP7s*

**DNp05:** *w[1118]/+; VT019060AD/UAS-CyRFP1; VT003280DBD/UAS-jGCaMP7s*

**DNp09:** *w[1118]/+; VT023490AD/UAS-CyRFP1; R38F04DBD/UAS-jGCaMP7s*

**DNp11:** *w[1118]/+; VT025392AD/UAS-CyRFP1; VT057247DBD/UAS-jGCaMP7s*

**DNp12:** *w[1118]/+; UAS-CyRFP1/+; GMR47E06-GAL4/UAS-jGCaMP7s*

**DNp18:** *w[1118]/+; VT064490AD/UAS-CyRFP1; R69C11DBD/UAS-jGCaMP7s*

**DNp32:** *w[1118]/+; R61H01AD/UAS-CyRFP1; R82C10DBD/UAS-jGCaMP7s*

Figure S3:

**DNg34:** *w[1118]/+; UAS-CyRFP1/+; GMR83A12-GAL4/UAS-jGCaMP7s*

**DNa01**: *w[1118]/+; R22C05AD/UAS-CyRFP1; R56G08DBD/UAS-jGCaMP7s*

**DNa02:** *w[1118]/+; R75C10AD/UAS-CyRFP1; R87D07DBD/UAS-jGCaMP7s*

**DNb05:** *w[1118]/+; VT019391AD/UAS-CyRFP1; VT028198DBD/UAS-jGCaMP7s*

**DNb06:** *w[1118]/+; R20C04AD/UAS-CyRFP1; VT025999DBD/UAS-jGCaMP7s*

**DNg13:** *w[1118]/+; VT027166AD/UAS-CyRFP1; VT009857DBD/UAS-jGCaMP7s*

Figures 3D left, 4A–C, S5A top, S6A–C, S6G left, S7E, and S7F (DNa02 optogenetic activation):

norpA[P24], nSyb-PhiC31/norpA[P24]; UAS-SPARC2-D-CsChrimson::tdTomato/R75C10AD; R87D07DBD/+

Figures 3D right, 4G–I, S5B, S6G right, S7E, and S7F (DNg13 current injection) and Figures 5A, 5B, 5D, 5E, S7A, S7B, S7D, 6A, 6B, and 6D (DNg13 electrophysiology recordings):

+; *UAS-mCD8::GFP/VT027166AD; VT009857DBD*/+ (backcrossed 5 generations into the isoD1 genetic background)

Figures 4D–F, S5A bottom, S6D–F, S6G middle, S7E, and S7F (DNa02 optogenetic inhibition)

nSyb-PhiC31/+; UAS-SPARC2-D-GtACR1::eYFP/R75C10AD; R87D07DBD/+

Figure 5A–C, 5E, S7A–C 6A, 6B, and 6D (DNa02 electrophysiology recordings):

+; *UAS-mCD8::GFP/R75C10AD; R87D07DBD*/+ (backcrossed 5 generations into the isoD1 genetic background)

#### Freely walking fly behavior

The behavior of freely walking flies was collected using the Coliseum^60^. Briefly, the Coliseum consisted of a 1 m × 1 m arena with a large monitor forming each of the 4 walls. Flies were automatically and individually dispensed into the arena through a hole in the floor using an automated dispenser. The flies were illuminated from below the floor using IR LEDs and tracked from above using a high-speed camera mounted on a 2-axis CNC (grbl with gShield + Arduino UNO; stepper motors SM42HT47-1684B), which maintained the fly in the camera’s field of view in closed-loop using the FlyVR software (github.com/ClandininLab/FlyVR). The video of the fly as well as the fly’s body position and angle in the frame and the position of the CNC were output by FlyVR at the end of each trial. Trials ended when tracking was lost for several seconds (because the fly left the arena by flying away). The frame rate of position tracking and the video was ~125 Hz. Video frames were 658 px × 494 px, and the resolution was 37 px/mm. Flies walked with a constant background (dark, gray, or bright) or a static pattern (vertical or horizontal sine gratings or a checkerboard) on the monitor walls around them; for subsequent analyses, flies across all of these wall backgrounds/patterns were pooled.

#### Tethered fly preparation and dissection

The fly preparation holder consisted of a 302 stainless steel foil with a bend in it secured to an acrylic platform. The bottom side of the holder was spray-painted a solid matte black (4WGC1, Tough Guy) to reduce reflections during behavioral monitoring (see Tethered fly behavioral setup). The foil had a teardrop-shaped hole photochemically etched into it (Etchit), into which the fly’s head and thorax were gently pushed. The fly was then secured to the foil with UV-cured glue (Loctite AA 3972) that was cured with brief (less than 1 s) pulses of UV light from a UV gun (LED-200, Electro-Lite Co.). For calcium imaging and electrophysiology experiments, the fly’s head was angled to allow access to the desired arbors or cell bodies. For calcium imaging, this meant that for most genotypes, the fly’s head was tilted forward such that the posterior cuticle was exposed; however, for DNg14, DNg15, DNg16, and DNg31, the head was gently rotated 180° around the neck such that the ventral side of the head was facing upwards. For optogenetic experiments, the fly’s head was secured at approximately its natural angle. For DNa02 electrophysiology experiments, the fly’s head was tilted backwards until it hit the thorax to expose more of the anterior surface, though the antenna were kept below the foil. For DNg13 electrophysiology experiments, the head was rotated 180° around the neck to expose the ventral side. The bend in the foil allowed the angle between the thorax and the abdomen to be better matched to that of freely walking flies, which was important for achieving good walking in these tethered flies. To prevent excessive grooming of the wings and for easier automated tracking of the legs, the blades of the wings were fully removed, but the wing hinges were left attached.

For calcium imaging and electrophysiology experiments, the dorsal side of the fly was covered in saline, and to expose the brain area of interest, forceps were used to make a hole in the head capsule and to remove overlying trachea and fat. To reduce movement of the brain, muscle 16 was severed with a hook fashioned out of tungsten wire. Additionally, muscles of the proboscis were severed using forceps, or in the case of flies with their head rotated 180°, the proboscis was entirely removed to provide access to the brain. For electrophysiology experiments, a hole was made in the perineural sheath in the area around the cell bodies of interest with forceps. For experiments investigating the effect of optogenetic stimuli on behavior (without calcium imaging or electrophysiology), the head capsule was not opened and the proboscis was left intact. However, as described below, saline was still perfused over the dorsal side of the fly to control the temperature and to buffer the thermal effects of the optogenetic stimulation.

The external saline solution contained (in mM): 103 NaCl, 3 KCl, 5 *N*-tris(hydroxymethyl) methyl-2-aminoethane-sulfonic acid, 8 trehalose, 10 glucose, 26 NaHCO_3_, 1 NaH_2_PO_4_, 1.5 CaCl_2_, and 4 MgCl_2_, with the osmolarity adjusted to 270–273 mOsm. The saline was bubbled with 95% O_2_ and 5% CO_2_, and it equilibrated at a pH of 7.3. The temperature was maintained at 28–30°C with an in-line heater (TC-324, Warner Instrument Corporation). The external saline was continuously superfused at a rate of approximately 2 mL/min.

#### Tethered fly behavioral setup

For all calcium imaging, optogenetic, and electrophysiology experiments, the fly’s walking behavior was monitored using a custom-built setup that enabled simultaneous extraction of the fly’s intended walking trajectory as well as its leg movements. This setup was inspired by previous work^118^. The fly walked on a ¼”-diameter acrylic sphere (1383K52, McMaster-Carr). This ball was first uniformly coated with 900-nm long-pass ink (Spectre 120, Epolin, diluted ~2:3 in cyclohexanone), and then spots of far-red fluorescent ink (excitation 793 nm, emission 840 nm; IR Ink 1, LDP LLC/maxmax.com, mixed 1:1 with clear acrylic paint (Slow-Dri Blending Medium, Liquitex)) were painted on by blending into a still-wet coat of clear acrylic paint. For the calcium imaging experiments, the ball floated above a plenum made of a 0.6 mL microcentrifuge tube into which another ¼”-diameter acrylic sphere was inserted such that the distance between the surface of the two balls was ~1.7 mm, which was the distance necessary for light passing through the two balls to be collimated. This 0.6 mL microcentrifuge tube was then inserted in a 1.5 mL microcentrifuge tube. Air flowed into the 1.5 mL tube from two sides, and small holes were made in the wall of the 0.6 mL tube above the fixed ball to allow the air to flow to support the ball on which the fly walked. For the optogenetic and electrophysiology experiments, this microcentrifuge tube-based plenum was replaced with a 3D printed one of an equivalent overall design. The ball was illuminated with a 780 nm LED (M780L3, Thorlabs) that had a 769/41-nm bandpass filter (FF01-769/41, Semrock) in front of it. The fly was illuminated with two 940 nm LEDs (M940L3, Thorlabs) that each had a 950 nm longpass filter (FELH0950, Thorlabs) in front. The movement of the ball was tracked at ~120 Hz in real time using FicTrac^110^, a program that computes the rotation of a sphere from video captured directly from a camera (here: GS3-U3-51S5M, FLIR; with lens M7528-MP, Computar; with a 832/37 nm bandpass filter, FF01-832/37, Semrock). FicTrac was modified to send real-time analog measurements of the position of the ball in all three rotational axes to a USB DAQ (USB-3101, Measurement Computing). These signals were digitized at 20 kHz by a 16-bit A/D converter (NI PXIe-6361, National Instruments) and acquired using the MATLAB Data Acquisition Toolbox (R2018a, MathWorks). A video of the fly’s leg movements was captured from below at a resolution 69 px/mm (image size: 416 px × 416 px) at 223 Hz with a 4 ms exposure time (calcium imaging experiments) or at 250 Hz with a 1.5 ms exposure time (optogenetic and electrophysiology experiments) using a GS3-U3-41C6NIR (FLIR) camera with a macro zoom lens (MLM3X-MP, Computar) through the SpinView software (FLIR). The image was reflected off of a silver mirror (CCM1-P01, Thorlabs), and one or two 950 nm longpass filters (FELH0950, Thorlabs) were positioned between the bottom of the ball plenum and the camera. Two filters were required to exclude the light from the two-photon laser during calcium imaging experiments; one filter was sufficient to exclude stray light for optogenetic and electrophysiology experiments.

#### Two-photon calcium imaging

Calcium imaging experiments were performed on a custom-built two-photon microscope controlled with ScanImage software^119^. jGCaMP7s and CyRFP1 were excited at 920 nm using a Mai-Tai HP Ti:sapphire laser with a DeepSee precompensation unit (Spectra-Physics). Power at the sample was kept to under ~20 mW. The objective used was either the 20×/NA1.0 XLUMPLFLN objective (Olympus) or the 25×/NA1.1 N25X-APO-MP objective (Nikon). jGCaMP7s fluorescence was collected using the H10770PA-40 SEL PMT (Hamamatsu) with a 525/50-nm bandpass filter (FF01-525/50-50, Semrock), and CyRFP1 fluorescence was collected using the R9110 PMT (Hamamatsu) with a 629/53-nm bandpass filter (FF01-629/56-50, Semrock). For all but DNp12, the imaging region for each cell type was a single plane selected to contain a portion of the dendritic arbor for both the left and the right cells. The imaging region for DNp12 instead contained the left and right cell bodies. The imaging region was 200 × 80 pixels, and it was scanned bidirectionally using a galvo-galvo system (6210HSM40, Cambridge Technology) with a pixel dwell time of 2400 ns. The final frame rate was 22.7 Hz.

#### Optogenetic activation experiments

Illumination for stimulating CsChrimson was provided by light from a mercury lamp (U-LH100HG, Olympus) passed through a 620/60 nm bandpass filter (49019-ET-Cy5 Longpass, Chroma) and focused onto the dorsal surface of the fly’s head and thorax using a 5×/NA0.15 MPlan FL N (Olympus) objective. The spot of illumination was 5.5 mm in diameter, and therefore covered the entire dorsal area of the fly exposed above the holder. ND filters were used to control the illumination intensity. For the data in Figures 3, 4, S6, and S7, the illumination power delivered to the fly was 0.09 mW/mm^2^. For each fly, two 250-s trials were performed, and each trial consisted of randomly interleaved 0.2 s and 2 s light pulses separated by 5 s without stimulation. The short and long pulses had similar overall effects: in both cases, the behavioral response was transient. Therefore, the shorter pulse duration was chosen as the focus (as described in *Data analysis - Figures 4,* S5, S6, and S7E–F).

To ensure that light pulses modulated DNa02 spike rate, patch-clamp recordings were performed (as described below in *Patch-clamp electrophysiology*) in flies of the same age and genotype as those used in the behavior experiments (Figure S5). Note that the fly’s cuticle was intact during the behavior experiments, but an aperture was opened in the cuticle to perform patch-clamp recordings; therefore, to approximate the light-absorbing effects of the fly’s cuticle^120^, additional ND filters were used in these patch-clamp recordings, such that the illumination power delivered to the fly was 0.02 mW/mm^2^ (rather than 0.09 mW/mm^2^). For each fly, two 250-s trials were performed, and each trial consisted of randomly interleaved 0.2-s and 2-s light pulses separated by 5 s without stimulation.

Using SPARC, the experimental and control flies were of the same genotype, so the behavioral measurements were performed blinded to which DNa02 cells, if any, expressed CsChrimson. Afterwards, the brain and ventral nerve cord were dissected out of the fly and placed on a microscope slide. The expression of CsChrimson in each DNa02 cell was determined by the fluorescence of the tdTomato tagged to the CsChrimson under epifluorescence imaging (excitation filter: 540/25 nm, emission filter: 575 nm longpass, 19004-AT-TRITC/Cy3 Longpass, Chroma). Expression of CsChrimson in non-DNa02 cells was also noted. In the brain, it was restricted to a single type of visual neuron with arbors in the lobula; on average, 2 cells per hemisphere expressed CsChrimson (range 0–4 cells). In the ventral nerve cord, a few cells in the abdominal ganglion expressed CsChrimson in a subset of flies.

SPARC-CsChrimson could not be used to produce specific unilateral stimulation of DNg13 because the transgenic line for this DN also drives GAL4 expression in an ascending neuron. In flies where this ascending neuron expressed CsChrimson, regardless of whether DNg13 expressed CsChrimson, light stopped locomotion. Very few flies had expression in one copy of DNg13 but zero copies of this ascending neuron, and so it was challenging to collect enough data to assess the effect of unilateral DNg13 stimulation alone. For this reason, direct current injection via patch-clamp electrophysiology was used to perturb DNg13 unilaterally.

#### Optogenetic inhibition experiments

Illumination for stimulating GtACR1 was provided by light from a mercury lamp (U-LH100HG, Olympus) passed through a 520/44 nm bandpass filter (FF01-520/44-25, Semrock) and focused onto the dorsal surface of the fly’s head and thorax using a 5×/NA0.15 MPlan FL N (Olympus) objective. The spot of illumination was 5.5 mm in diameter, and therefore covered the entire dorsal area of the fly exposed above the holder. ND filters were used to control the illumination intensity. For the data in Figures 4, and S6, and S7, the illumination power delivered to the fly was 2.1 mW/mm^2^. For each fly, three 360-s trials were performed. Each trial consisted of 2 s light pulses separated by 1.5 s without light. Simultaneously, visual stimuli were presented to the fly using a circular arena composed of modular square panels that each had an 8×8 array of blue LEDs (460–475 nm peak, KEM-12088-AB, Dongguan Houjie Keming Electronic Factory)^121^. The circuit boards for the arena and its controller were made by Bittele, the PCBs by PCBCart, and the final setup was assembled by the Research Instrumentation Core at Harvard Medical School. A single pixel subtended approximately 2.8° of the fly’s visual field, and the entire arena subtended 270°, with the 90° gap located directly behind the fly. The visual stimulus refreshed at a rate ≥372 Hz. The visual stimulus presented was repeated trials of 2 s gray, 1 s static grating, and 0.5 s moving grating, where the grating was a full-contrast, square-wave grating where each stripe was 6 LEDs (~17°) wide. The mean luminance was preserved across the trial. On each trial, the grating randomly moved either clockwise or counterclockwise at 72°/s. The optogenetic light pulse was coordinated with the visual stimulus such that the light turned on when the static grating appeared and turned off 0.5 s after the grating stopped moving and the arena was gray. In 25% of the trials, the optogenetic light pulse was omitted. This visual stimulus was designed to elicit an optomotor response (a turn in the same direction as the moving grating), as we thought that might accentuate the effect of DNa02 hyperpolarization. However, a more robust effect of DNa02 hyperpolarization was elicited at the onset of the optogenetic light pulse, where the visual stimulus transitioned from gray to a static grating, likely because there is some adaptation over time to the optogenetic manipulation. Thus, analyses (described in *Data analysis - Figures 4,* S5, S6, and S7E–F) focused on the time period at the onset of the optogenetic light pulse.

To ensure that light pulses modulated DNa02 spike rate, patch-clamp recordings were performed (as described below in *Patch-clamp electrophysiology*) in flies of the same age and genotype as the behavior experiments. Note that the fly’s cuticle was intact during the behavior experiments, but an aperture was opened in the cuticle to perform patch-clamp recordings; therefore, to approximate the light-absorbing effects of the fly’s cuticle^120^, additional ND filters were used in these patch-clamp recordings, such that the illumination power delivered to the fly was 0.11 mW/mm^2^ (rather than 2.1 mW/mm^2^). For each fly, two 300-s trials were performed, and each trial consisted of randomly interleaved 0.2 s and 2 s light pulses separated by 3 s without stimulation.

Using SPARC, the experimental and control flies were of the same genotype, so the behavioral measurements were performed blinded to which DNa02 cells, if any, expressed GtACR1. Afterwards, the brain and ventral nerve cord were dissected out of the fly and placed on a microscope slide. The expression of GtACR1 in each DNa02 cell was determined by the fluorescence of the eYFP tagged to the GtACR1 under epifluorescence imaging (excitation filter: 520/44 nm, emission filter: 561 nm longpass, Semrock). Expression of GtACR1 in non-DNa02 cells was also noted. In the brain, it was restricted to a single type of visual neuron with arbors in the lobula; on average, 2–3 cells per hemisphere expressed GtACR1 (range 0–6 cells). In the ventral nerve cord, a few cells in T1 and T2 expressed GtACR1 in a subset of flies.

#### Patch-clamp electrophysiology

Patch pipettes of borosilicate glass (O.D. 1.5 mm, I.D. 0.86 mm, Sutter) were made the day of the recording using a Flaming/Brown micropipette puller (Model P-97, Sutter). Pipette resistances were 4.5–6.5 MΩ. The internal solution contained (in mM): 140 potassium aspartate, 10 4-(2-hydroxyethyl)-1-piperazineethanesulfonic acid, 4 MgATP, 0.5 Na_3_GTP, 1 ethylene glycol tetraacetic acid, 1 KCl, and 15 neurobiotin citrate. The pH was 7.2 and the osmolarity was adjusted to 265 mOsm.

Patch-clamp recordings were achieved under visual control using a custom-built microscope made from a DIY Cerna body (Thorlabs) and the trinoculars and epifluorescence illumination optical path of an Olympus BX51W1. The camera was a GS3-U3-41C6NIR (FLIR). The objective was the 40x/NA0.8 LUMPlanFL N water dipping objective from Olympus. The brain was illuminated with the same light source as the ball (described above in Tethered fly behavioral setup); this was a 780 nm LED (M780L3, Thorlabs) that had a 769/41-nm bandpass filter (FF01-769/41, Semrock) in front of it. The desired cell was identified based on GFP expression and the location of the cell body, except for recordings from SPARC-CsChrimson and SPARC-GtACR1 flies, where the desired cell was instead identified based on expression of the tagged fluorophore–tdTomato for CsChrimson and eYFP for GtACR1–and the location of the cell body. GFP was excited using illumination from either a 470 nm LED (M470L2-C1, Thorlabs) or a mercury lamp (U-LH100HG, Olympus) passed through a 480/40-nm filter (49012-ET-FITC/EGFP Longpass, Chroma). The emission filter was a 510 nm longpass filter. TdTomato was excited using illumination from a mercury lamp passed through a 540/25-nm filter (19004-AT-TRITC/Cy3 Longpass, Chroma); the emission filter was a 575 nm longpass filter. eYFP was excited using illumination from a mercury lamp passed through a 520/44-nm filter (FF01-520/44-25, Semrock); the emission filter was a 561 nm longpass filter (LP02-561RU-25, Semrock).

To expose the targeted cell body to access by the patch pipette, neighboring cell bodies and glia were first cleared away by applying positive and negative pressure through large-bore cleaning pipettes (pulled from O.D. 1.5 mm, I.D. 1.1 mm borosilicate glass, Sutter) filled with external saline. Ensheathing glia around DNg13’s cell body were removed by brief application of dispase (1 mg/mL in external saline, Worthington Biochemical) delivered through a pipette.

Recordings were obtained using an Axopatch 200B amplifier and a CV-203BU headstage (Molecular Devices). Analog voltage signals were lowpass filtered at 5 kHz (Bessel filter, 80 dB/decade), digitized at 20 kHz by a 16-bit A/D converter (NI PXIe-6361, National Instruments), and acquired using the MATLAB Data Acquisition Toolbox (R2018a, MathWorks). Liquid junction potential correction was performed post-hoc by subtracting 13 mV from the recorded voltages^122^.

#### Current injection experiments

During DNg13 patch clamp recordings, a current step (either −75 pA or 100 pA, presented in a random sequence) was injected for 0.5 s every 2.5 s, or for 1 s every 3 s. Half of the flies experienced the short protocol and half of the flies experienced the long protocol. The short and long current injection steps had equivalent effects on the behavior and spike rate (i.e. neither adapted during the step), and so the data were pooled for subsequent analyses (described in *Data analysis - Figures 4,* S5, S6, and S7E–F).

Hyperpolarizing current injection into DNg13 could suppress spike generation in this cell almost completely, whereas depolarizing current injection into DNg13 could reliably elevate the spike rate to >100 spikes/s. However, using current injection into DNa02, large and reliable changes in spike rate could not be generated. This could occur if the spike initiation zone in DNa02 is electrotonically distant from the soma. In any event, this precluded us from using current injection to create bidirectional perturbations of DNa02 activity, as we did for DNg13.

#### Data processing - Freely walking fly behavior

Using Animal Part Tracker (APT)^61^ running the Mixture Density Model (MDN) deep convolutional network, the tips of the 6 tarsi, the front of the head, 3 points on the thorax (both postpronotal lobes and the posterior tip of the scutellum), the tip of the abdomen, and the 2 wing tips were tracked in each frame of the videos acquired from the Coliseum^60^. Using this position within the camera frame and the position of the CNC, the absolute position in world coordinates of each tracked point was extracted. From the tracked points, the position of the tarsi was extracted in units of body length in the coordinate frame defined by the fly’s anterior-posterior and medial-lateral axes. Specifically, a line was fit to the head, the midpoint between the postpronotal lobes, the posterior tip of the scutellum, and the tip of the abdomen to define the anterior-posterior axis of the body. The head, scutellum, and abdomen tracked points were projected to this axis; the distance between the head and abdomen projected points was defined as the fly’s body length and the scutellum point as the midpoint of the fly. The medial-lateral axis was defined as the axis orthogonal to the anterior-posterior axis that passed through the fly’s midpoint. The angle of the vector from head to abdomen along the anterior-posterior axis was the fly’s heading angle. This angle was used to disambiguate the heading angles computed by FlyVR (described below) but was not used to compute any body velocity parameters. After projecting the tarsi points to the fly’s coordinate frame, outliers were removed using the following procedure: the median filtered position was computed over a 5-frame window, and outliers that deviated by more than 50% of the range in median-filtered values over a 500-frame window were replaced with the median-filtered value. The maxima and minima of position in the anterior-posterior axis were extracted for each leg. Individual steps of the walking stride cycle were defined as a single complete cycle from posterior to anterior to posterior, and the following parameters were computed on each step:

- Anterior extreme position (AEP) - the position in both the anterior-posterior and medial-lateral axes of the tarsal tip when it was at its most anterior position of the stride cycle
- Posterior extreme position (PEP) - the position in both axes of the tarsal tip when it was at its most posterior position of the stride cycle
- Stride length - the distance between the AEP and the PEP in the anterior-posterior axis
- Step direction - the angle of the vector from the AEP to the PEP
- Stance or swing - for each half step (posterior to anterior or anterior to posterior), the translational velocity of the leg in the world coordinate frame was computed. The half step where the translational velocity was smaller (i.e. the leg was stationary relative to the ground) was called stance, and the half step where the velocity was greater (i.e. the leg was moving relative to the ground) was called swing.
- Return stroke position - the AEP in both axes at the end of the swing phase (i.e. only when the leg is walking forward)
- Power stroke position - the PEP in both axes at the end of the stance phase (i.e. only when the leg is walking forward)

The phase of each leg during each frame was computed by assigning the phase to be 0° for the frame at the start of the swing phase for each step (based on the swing/stance calls), 180° for the frame at the swing/stance transition, and 360° for the frame at the end of the stance phase, and then for each half step, linearly interpolating between these values using the position in the anterior-posterior axis. Spline fits to the maxima and minima of position in the anterior-posterior axis were used to compute continuous estimates of the AEP and PEP. The difference between the AEP and PEP was the continuous estimate of stride length. The angle of the vector from the AEP to the PEP was the continuous estimate of step direction.

The FlyVR software extracted the position and angle of the fly within the camera frame by fitting an ellipse to the body; the body position was the centroid of the ellipse, and the body angle was the angle of the long axis of the ellipse. This measure of body angle did not distinguish between the front and back of the fly, so it was compared with the fly’s heading angle from the leg tracking procedure, and any ~180° deviations were corrected. The absolute body position in world coordinates was computed by summing the fly’s position in the camera frame with the position of the CNC. The body position and the heading angle were smoothed using a 10-frame gaussian-weighted moving average. The fly’s rotational velocity was the change in heading angle. The fly’s forward velocity was the change in position in the same direction as the heading angle. The fly’s lateral velocity was the change in position in the direction orthogonal to the heading direction. These body velocities were smoothed using a 15-frame Gaussian-weighted moving average. A more heavily smoothed version of the body velocities was also generated using a 50-frame Gaussian-weighted moving average instead; as described below, these velocities were used to identify turning bouts.

#### Data processing - Calcium imaging

Raw images in each time series were motion-corrected using the NoRMCorre software package^111^ to perform rigid subpixel registration of the jGCaMP7s channel. The CyRFP1 channel was shifted to match the jGCaMP7s channel. Regions of interest (ROIs) around the arbors of individual neurons were selected by thresholding on the ΔF/F signal computed over space in the time-series averaged image in the jGCaMP7s channel (i.e. for pixel i, F_i_/mean(F_all pixels_) − 1) and then by manually selecting the region of the thresholded mask to include. For each frame in the time series, intensity values for the pixels within each ROI were averaged and the mean background value (the average intensity in a region of the image without cells) was subtracted for each imaging frame within the time series. To correct for bleaching, the background-subtracted intensity time series for each ROI was then fit with the sum of two exponentials, and F(t)/F_0_ was calculated where F_0_ was the fitted value at each time t. F(t)/F_0_ was computed independently for the jGCaMP7s and CyRFP1 channels. To further account for motion artifacts, the jGCaMP7s signal was normalized to the CyRFP1 signal such that ΔF/F was (jGCaMP7s F(t)/F_0_) / (CyRFP1 F(t)/F_0_) − 1. The right-left difference and the right+left sum in ΔF/F was computed for the ROIs corresponding to the right and left cells for each trial.

#### Data processing - Tethered fly behavior

Raw FicTrac signals of the position of the ball in all three axes (yaw, pitch, and roll) were low-passed filtered at 40 Hz and then downsampled to 1000 Hz. These signals were then smoothed with a 200 ms time window Gaussian-weighted moving average. The fly’s velocity in each of the three axes (rotational, forward, and lateral) was computed by finding the derivative of the position signal. Rotational velocity was defined as the rate of change in the fly’s heading angle over time; this was taken as the opposite of the ball’s yaw velocity. Forward velocity was defined as the rate of change in position over time in the direction parallel to the fly’s anterior-posterior axis; this was taken as the opposite of the ball’s pitch velocity. Lateral velocity was defined as the rate of change in position over time in the direction orthogonal to the fly’s anterior-posterior axis; this was taken as the opposite of the ball’s roll velocity. The velocity signals were then smoothed with a 100 ms time window Gaussian-weighted moving average. The fly’s forward/rotational/lateral acceleration was computed by further smoothing the fly’s forward/rotational/lateral velocity signal with a 250 ms window Gaussian-weighted moving average, computing the derivative, and then smoothing again with a 250 ms window Gaussian-weighted moving average.

Rotational velocity and lateral velocity were overall highly correlated. Also, DNa02 and DNg13 spike rates correlated with lateral velocity, just as they did with rotational velocity, and DNa02 and DNg13 perturbations had effects on lateral velocity that mirrored their effects on rotational velocity. Our analyses focused on rotational velocity, but it should be kept in mind that steering generally involves concerted changes in both rotational and lateral velocity.

Using Animal Part Tracker (APT)^61^ running the Mixture Density Model (MDN) deep convolutional network, the tips of the 6 tarsi, the front of the head, the 3 midpoints between the 3 pairs of legs, and the tip of the abdomen, were tracked in each frame of the videos acquired from below the fly in our tethered fly behavioral setup. One network was trained for all of the tethered fly data, and it was a separate network from the one used for the freely walking flies. Tracked points were converted to positions in the fly’s coordinate frame and then to step parameters as described for the *Data analysis* - Figures 1 and S1 section, except for the following differences. The line used to define the anterior-posterior axis of the fly was fit directly to the head, the midpoints between each pair of legs, and the tip of the abdomen. The angle of the vector between the head and the abdomen points was not a readout of the fly’s heading as it was fixed; the FicTrac signal alone was used for heading. For each step, swing was defined as the half step with the shorter duration, and stance was the half step with the longer duration. Swing and stance were computed in this manner because it was not possible to determine whether the leg was moving or stationary relative to the ground when the ground (the ball) was also moving; however, the duration of the half-steps was a reliable readout of swing/stance as swing is always shorter than stance^66^.

For the calcium imaging experiments, time periods where the fly was not walking were determined by setting a threshold on the total speed across all three axes. This threshold was manually adjusted for each trial, and time points that fell below the threshold were preliminarily called not walking. The minimum duration of each walking or not-walking bout was 200 ms, and bouts that were too short were merged with their prior bout (i.e. the walk/not-walk call was flipped) until they exceeded the minimum duration.

For the optogenetic and electrophysiology experiments, time periods where the fly was not walking were determined by setting a threshold on the velocity of the two middle legs and on the total speed across all three body velocity axes. The thresholds were manually adjusted for each trial, and time points that fell below both thresholds were preliminarily called not walking. The minimum duration of each walking or not-walking bout was 200 ms, and bouts that were too short were merged with their prior bout (i.e. the walk/not-walk call was flipped) until they exceeded the minimum duration.

#### Data processing - Electrophysiology

The time when each spike occurred was determined as the time of threshold crossing in the first derivative of the membrane potential. Threshold crossings that were less than 2 ms after the prior threshold crossing were excluded. The membrane potential threshold was determined manually for each trial. Spike rate was then computed as the inverse of the interspike interval, which was computed as the derivative of the vector of spike times.

#### Data processing - Connectomics

DNa02 was identified in the FAFB/FlyWire full *Drosophila* female brain connectome^23,26^ by manually comparing the morphology of every neuron that had its primary neurite in the appropriate tract with the light microscopy images of DNa02^29^. DNg13 was identified in FlyWire by first using natverse^112^ to register the light microscopy image of DNg13^29^ into the FAFB space to find the approximate location of the midline-crossing neurite and then by manually examining the morphology of every neuron that crossed the midline anywhere near that location. DNa02 was identified in the FANC full *Drosophila* female ventral nerve cord connectome^25^ by the lab of Gregory Jefferis. DNg13 was preliminarily identified in FANC by the Jefferis lab and that identification was then confirmed in this work by manual comparison of the morphology of every neuron that passed through the left half of the neck connective with the light microscopy images of DNg13^29^.

The connectivity of DNa02 and DNg13 in the brain was obtained from FlyWire^23,26,71,123,124^ using the BrainCircuits.io platform. Data was from the fruitfly_fafb_flywire_public API accessed on September 27, 2023. Each neuron presynaptic to DNa02 or DNg13 (minimum number of synapses = 10) was categorized into one of six categories by manual examination of its morphology. The categories were:

- Motor-associated - the input neuron arborizes predominantly in motor-associated brain regions (LAL, VES, IPS, SPS, EPA, or GNG)
- Descending neuron - the input neuron has a cell body in the brain and projects out of the brain through the neck connective
- Ascending neuron - the input neuron has a process in the neck connective and no cell body in the brain
- Visual - the input neuron has a dendritic arbor in a visual region (optic lobe, PVLP, AVLP, PLP, or AOTU)
- Superior brain - the input neuron’s dendrites are primarily in superior brain regions (SMP, SIP, SLP, MB, or LH)
- Central complex - the input neuron arborizes in the central complex (EB, PB, FB, NO, or AB; in practice, all of these neurons are PFL3).

Brain region abbreviations follow standard conventions^125^. Monosynaptic input neurons for the DNa02 and DNg13 cells in each hemisphere were considered to be shared if they made 10 or more synapses onto each of the two DNs. They were considered to be unique inputs if they made 10 or more synapses onto one neuron but not the other.

The connectivity of DNa02 and DNg13 in the ventral nerve cord was obtained from FANC using the fancr package in natverse (https://github.com/flyconnectome/fancr). Many of the neurons downstream of DNa02 and DNg13 were proofread as part of a recent independent study^126^. The connectivity was last pulled on September 3, 2023. Motor neuron IDs were from the CAVEclient fanc_v4 tables: neck_motor_neuron_table_v0, haltere_motor_neuron_table_v0, wing_motor_neuron_table_v0, all_leg_motor_neuron_table_v0, and motor_neuron_table_v7^27^. Each neuron postsynaptic to DNa02 or DNg13 (minimum number of synapses = 10) was first categorized as likely leg or wing based on its morphology and basic connectivity:

- Leg - the neuron is a leg motor neuron, a leg premotor neuron, or arborizes in the leg neuromeres
- Wing - the neuron is a wing, haltere, or neck motor neuron or it arborizes in the dorsal neuropils and is not a leg premotor neuron

Neurons in the leg category were then divided into six further categories:

- Motor neuron - leg motor neuron
- Local premotor - interneuron whose arbors are restricted to one leg neuromere and that synapses onto at least one leg motor neuron
- Local - interneuron whose arbors are restricted to one leg neuromere and that does not synapse onto any leg motor neurons
- Intersegmental/bilateral premotor - neuron that arborizes in more than one leg neuromere, either bilaterally or intersegmentally, and synapses onto at least one leg motor neuron
- Intersegmental/bilateral - neuron that arborizes in more than one leg neuromere, either bilaterally or across segments, and that does not synapse onto any leg motor neurons
- Ascending neuron - neuron that has its cell body in the ventral nerve cord and sends a projection into the neck connective

Neurons in the wing category were then further divided into six categories:

- Wing motor neuron
- Haltere motor neuron
- Neck motor neuron
- Ascending neuron
- Unilateral interneuron - arborizes on one side of the dorsal neuropils
- Bilateral interneuron - arborizes on both sides of the dorsal neuropils

Monosynaptic output neurons for DNa02 and DNg13 were considered to be shared if they received 10 or more synapses from each of the two DNs. They were considered to be unique outputs if they received 10 or more synapses from only one of the two neurons.

Neurotransmitter predictions were made by identifying the hemilineage each neuron belonged to based on anatomical similarity to the published descriptions of the neurons in each hemilineage and assigning the neuron’s neurotransmitter as the reported neurotransmitter for that hemilineage^72,127,128^.

In the Discussion, we note that approximately 20% of DNs project unilaterally and form output synapses in all three leg neuromeres; this information was obtained from the manc:v1.2.1 connectome^28,70,129^ (using neuPrint+^130^) by identifying the number of neurons of class “descending neuron” that had synaptic output (>0 synapses) in three homolateral leg neuromeres (Output Brain Regions: LegNp(T1)(L), LegNp(T2)(L), and LegNp(T3)(L) or LegNp(T1)(R), LegNp(T2)(R), and LegNp(T3)(R)) and none of the other leg neuromeres.

#### Data analysis - Figures 1 and S1

For plotting the example trajectories in Figures 1B and 1D, body and leg positions were resampled to 100 Hz from the acquisition rate of ~125 Hz.

Turning bouts were identified by finding peaks (using the MATLAB findpeaks function) in the aggressively smoothed version of the rotational velocity. The start and end of the bouts were defined as when the rotational velocity fell below 10°/s. Only bouts with a duration (end time – start time) of 0.3–0.7s were included in the subsequent analyses. Bouts where the fly turned left and bouts where the fly turned right were both extracted, with the left turn bouts being identified in the sign-inverted rotational velocity. Right and left turning bouts were combined by inverting the rotational velocity and step direction values and by swapping the left and right leg step parameter values for left turning bouts.

The step parameter values for each bout were the values during the step (for each leg) that occurred when the rotational velocity peaked. They were expressed as the change from the mean value across all flies when they were not turning (rotational speed < 10 °/s). In Figure 1C and Figure S1B, only bouts with a forward velocity of 10–15 mm/s at the peak in the rotational velocity were included. Bouts were then binned by their peak rotational velocity. In Figures 1E and S1C, only bouts with a forward velocity of 10–15 mm/s at the start of the bout and a peak rotational velocity of 50–150 °/s were included. Bouts were categorized as pivots if the difference between the forward velocity at the start of the bout and at the rotational velocity peak (peak – start) was less than −1 mm/s. In our dataset, in a typical fly, pivots outnumbered swerves. Swerves were bouts where the difference between the peak and the start was greater than 1 mm/s. Pivots and swerves are not discrete; rather, they exist on a continuum of forward velocity values. Violin plots were generated using Violin Plot (MATLAB Central File Exchange) and represent Gaussian kernel estimates fit to all of the data points.

As step direction is a circular parameter, all descriptive and inferential statistics on it, in this and subsequent figures, were performed with the CircStat Toolbox^113^. For Figures S1B and S1C, Violin Plot was modified with CircStat functions to plot step direction appropriately.

For Figure 1C, two-way, unbalanced ANOVAs with leg identity and peak rotational velocity (including a no turning reference) as factors were performed and followed up with post-hoc Tukey-Kramer tests against reference for each rotational velocity and each leg.

For Figures 1E and S1C, except for step direction, two-way unbalanced ANOVAs with leg identity and pivot/swerve as factors were performed and followed up with post-hoc Tukey-Kramer tests comparing pivots and swerves for each leg.

For step direction (Figures S1B and S1C right), parametric Watson-Williams multi-sample tests for equal means (CircStat Toolbox) were performed comparing pivot and swerve for each leg. Holm-Bonferroni correction for multiple comparisons was applied.

#### Data analysis - Figures 2 and S2

The heatmaps in Figure 2A were generated by plotting the mean right-left difference in ΔF/F for each of the rotational and forward velocity bins (30 bins each) for single example flies. ΔF/F was interpolated to 100 Hz. Only points from when the fly was walking were included in the heatmap. Data from transitions between walking and not walking periods were also excluded (0.14 s after the start of walking and 0.25 s before the end of walking). The time windows for exclusion were chosen to approximate the on kinetics of jGCaMP7s^106^. Bins were colored gray if there were fewer than 20 data points.

For Figures 2B, S2A, and S2D, each fly’s mean rotational velocity (as the difference from that fly’s mean rotational velocity) was plotted against the right-left difference in ΔF/F (30 bins). For Figures S2B and S2E, each fly’s mean forward velocity (as the difference from that fly’s mean forward velocity) was plotted against the right+left sum in ΔF/F (30 bins). Similarly to Figure 2A, ΔF/F was interpolated to 100 Hz, and only points from when the fly was walking and not transitioning between walking and not walking were included. Bins with fewer than 50 data points were excluded. The mean across flies was also plotted. The Pearson correlation coefficient between the behavioral parameter and ΔF/F was computed for each fly and the mean ± SEM across flies is displayed in the figures.

For Figures 2C and 2D, linear filters relating the right-left difference in ΔF/F and the fly’s rotational velocity were estimated by computing the first-order Wiener kernel^131^ for each fly. Both ΔF/F and rotational velocity were interpolated to 100 Hz. In the frequency domain, the kernel F(ω) = I*(ω)R(ω)/I*(ω)I(ω), where I(ω) and R(ω) are the input and response, respectively. ω is the frequency and * indicates the complex conjugate. The kernels were computed in the frequency domain, low pass filtered according to c(ω) = ê(−abs(ω − f_cut_)/f_τ_) for abs(ω) ≥ f_cut_. f_cut_ was 4 Hz and f_τ_ was 1 Hz. For Figure 2C, ΔF/F was the input and rotational velocity was the response. For Figure 2D, rotational velocity was the input and ΔF/F was the response. The kernels were computed for each fly using all data, regardless of whether the fly was walking or not, and then averaged across flies. The time axis in Figure 2C was inverted so negative time values were when ΔF/F preceded behavior for both types of filters.

For Figures S2C and S2F, the mean right+left sum in ΔF/F was calculated for each fly when it was walking and when it was not walking. Paired t-tests for a difference between walking and not walking (paired by fly) were performed for each cell type.

#### Data analysis - Figures 3, S4B, and S4C

Figure 3A displays the number of neurons that are presynaptic to DNa02 alone, DNg13 alone, or shared between the two for each hemisphere. Figure S4B shows the total number of synapses made onto the DNs by the neurons in each of those categories.

In Figure 3B, monosynaptic inputs of DNa02 and DNg13 were grouped by category; each neuron was treated equally and the plot expresses category membership as the percent of the total number of input neurons. Figure S4C totals up the number of synapses made onto the DNs by neurons in each category, and the plot displays the number of synapses each category contributes, as a percent of the total number of synapses.

For DNa02 in Figure 3D, the duration of optogenetic stimulation was 200 ms, and the illumination power was 0.09 mW/mm^2^. For DNg13, the duration of the current injection was 1 s, and the amplitude was 100 pA.

#### Data analysis - Figures 4, S5, S6, and S7E–F

For Figures 4A–C, S6A–C, S6G (left), and S7E, the duration of optogenetic stimulation of CsChrimson was 200 ms and the illumination power was 0.09 mW/mm^2^. For Figures 4D–F, S6D–F, S6G (middle), and S7E, the duration of optogenetic stimulation of GtACR1 was 2 s and the illumination power was 2.1 mW/mm^2^. For Figures 4G–I, S6G–I, S6G (right), and S7F, the duration of current injection was either 0.5 or 1 s. For hyperpolarization (−75 pA current injection step), only trials where the spike rate was suppressed below 5 spikes/s were analyzed; for depolarization (100 pA current injection step), only trials where the spike rate was increased to above 130 spikes/s were analyzed. For Figures 4A–C, 4G–I, S6A–C, S6G (left and right), S7E (CsCh), and S7F, stimulation trials were only included if the fly maintained an average forward velocity of 1 mm/s or greater from 200 ms before the start of the stimulation to 200 ms after the end of stimulation. Matched non-stimulated epochs for each fly were defined as a time window that matched the stimulation duration in the middle of each inter-stimulation period and that met the forward velocity criteria. For Figures 4D–F, S6D–F, S6G (middle), and S7E (GtACR1), stimulation trials were only included if the fly maintained an average forward velocity of 1 mm/s or greater from 500 ms before the start of the stimulation to 500 ms into the stimulation. The non-stimulated epoch was taken to be the 500 ms immediately prior to the start of stimulation for each trial.

For Figures 4A, 4D, 4G, S6A, and S6D, the plotted mean rotational velocity for each fly was the mean cumulative change in heading direction across perturbation trials minus the mean value during non-stimulated epochs in the same fly, divided by the time window of consideration (the start of stimulation (fictive stimulation start for non-stimulated epochs) to 500 ms after the start of stimulation, chosen to approximate the duration over which perturbation affected spike rate (Figure S5) plus the latency between spike rate and behavior (Figure 6A)). The plotted mean forward velocity for each fly was computed identically, except with the mean cumulative forward displacement instead of heading direction. For Figures 4A, 4D, S6A, and S6D, two-sample t-tests were performed comparing ±CsCh (Figures 4A and S6A) or ±GtACR1 (Figures 4D and S6D) for the difference in rotational velocity and the difference in forward velocity. For Figure 4G, two-sample t-tests comparing hyperpolarization or depolarization trials with no-stimulation trials were performed.

In Figures 4B, 4C, 4H, 4I, S6B, S6C, S6G (left), and S6G (right), the change in the step parameter was measured as the mean value during perturbation, minus the mean value during non-stimulated epochs in the same fly. The change was measured using steps that fell between the start of the stimulation to 0.1 s after the end of stimulation. These time windows are different from those of Figures 4A, 4D, 4G, S6A, and S6D because step parameters are computed only once per step cycle. In Figures 4E, 4F, S6E, and S6F, the change in the step parameter was measured as the mean value for steps that fell during the first 500 ms of the optogenetic stimulation, minus the mean value of steps that fell during the non-stimulated epoch (the 500 ms immediately prior to the start of the optogenetic stimulation), in the same fly.

For Figures 4B, 4C, 4E, 4F, 4H, 4I, S6B, S6C, S6E, and S6F, mixed ANOVAs with legs (front, mid, or hind) and side (ipsilateral or contralateral) as within-subjects variables and CsChrimson expression (Figures 4B, 4C, S6B, S6C), GtACR1 expression (Figures 4E, 4F, S6E, and S6F), or depolarization/hyperpolarization (Figures 4H and 4I) as the factor were performed. These tests were followed up with post-hoc Tukey-Kramer tests comparing ±CsChrimson, ±GtACR1, or depolarization/hyperpolarization for each side. Mixed ANOVAs with leg identity (ipsilateral front, mid, or hind, or contralateral front, mid, or hind) as the within-subjects variables and CsChrimson expression (Figures 4B, 4C, S6B, S6C), GtACR1 expression (Figures 4E, 4F, S6E, and S6F), or depolarization/hyperpolarization (Figures 4H and 4I) as the factor were also performed and followed up with post-hoc Tukey-Kramer tests comparing ±CsChrimson, ±GtACR1, or depolarization/hyperpolarization for each leg.

In Figure S6G, parametric two-way ANOVAs for circular data with interaction were performed, with leg identity and ±CsChrimson, ±GtACR1, or depolarization/hyperpolarization as factors. For DNa02, unilateral and bilateral expression were separately compared with no expression. For experiments with significant differences, post-hoc parametric Watson-Williams two-sample tests for equal means, comparing ±CsChrimson, ±GtACR1, or depolarization/hyperpolarization conditions for each leg were performed. Holm-Bonferroni correction for multiple comparisons was performed.

For the CsChrimson trace in Figure S5A, the duration of optogenetic stimulation was 200 ms, and the illumination power was 0.02 mW/mm^2^. For the GtACR1 trace in Figure S5A, the duration of optogenetic stimulation was 2 s, and the illumination power was 0.11 mW/mm^2^. For the depolarization trace in Figure S5B, 100 pA of current was injected for 1 s. For the hyperpolarization trace in Figure S5B, −75 pA of current was injected for 1 s. The two traces are from the same fly.

For Figures S7E and S7F, the difference in the return or power stroke position was plotted in two-dimensions (anterior-posterior and medial-lateral axes). For slow – fast forward velocity, steps that occurred when the fly was walking straight (rotational speed < 10°/s) and at slow (1–8 mm/s) or fast (>8 mm/s) forward velocities were included; for each fly, the difference plotted was the mean for the steps when the fly was walking at the fast forward velocity subtracted from the mean for the steps when the fly was walking at the slow forward velocity. The flies included were all the flies used in the DNa02 and DNg13 manipulation experiments presented in these two figure panels; steps were excluded if they fell during stimulation or within 0.5 s after the end of stimulation. Leg positions during straight, unmanipulated walking were comparable across all of these flies, and thus the data were combined. For straight walking, the change in leg positions should be identical for the ipsilateral and contralateral sides, and this indeed held true for our observations. However, in case there were systematic biases, from how flies were positioned on the ball, for example, ipsilateral and contralateral legs were kept separate. In Figure S7E, for comparison with DNa02 manipulations, the data for the three ipsilateral legs were plotted, as unilateral DNa02 manipulation largely affects the ipsilateral side. In Figure S7F, for comparison with DNg13 manipulations, the data for the three contralateral legs were plotted, as unilateral DNg13 manipulation largely affects the contralateral side. For all of the manipulation data, the same flies and the same steps for each fly as in Figures 4 and S6 were included; the only difference from Figures 4 and S6 was which particular step parameters were plotted.

#### Data analysis - Figures 5 and S7A–D

Behavioral variables were compared with normalized DN spike rate 100 ms prior. The precise latency between a DN spike and the resulting change in behavior is unknown, and this latency may be different for different DNs and different step parameters, but a fixed 100-ms offset was used for all DNs and all step parameters, in order to avoid overfitting. If the “best” offset for each DN and each step parameter is used, the strength of the measured correlation with DN spiking increases, but this improvement may not be biologically meaningful due to overfitting.

Normalized spike rate was computed by subtracting the mean spike rate when the fly was not walking (which is typically very low in these DNs) and then dividing by the 95^th^ percentile value of the resulting distribution. For the stride parameters, continuous estimates were used rather than their values per step. Ipsilateral/contralateral stride length was defined as the average of the mean subtracted stride length for each of the three legs ipsilateral/contralateral to the recorded DN soma. Ipsilateral/contralateral return stroke position was the average of the return stroke position for all ipsilateral/contralateral legs, with values from each leg centered by subtracting the mean for that leg. Ipsilateral/contralateral power stroke position was the average of the power stroke position for all ipsilateral/contralateral legs, with values from each leg centered by subtracting the mean for that leg. Note that, when data was pooled across homolateral legs, they were weighted equally, but in truth, these DNs may not have an equal influence on all legs. The three homolateral legs were weighted equally to avoid overfitting; if the “best” relative weighting of the legs is used, the strength of the measured correlation with DN spiking increases, but this improvement may not be biologically meaningful due to overfitting.

For Figures 5B–D, the mean in the behavioral variable was computed for each fly across 50 bins of normalized DN spike rate. Only data from times when the middle and hind legs were stepping forward (based on step direction) were included. Data were interpolated to 250 Hz. Bins with fewer than 50 data points were excluded from the mean. The Pearson correlation coefficient between the behavioral parameter and the spike rate was computed for each fly and the mean ± SEM across flies is displayed in the figure panels. For Figures S7C and S7D, the data was further split into times when the fly’s rotational speed was less than 25 °/s (not turning) and times when it was greater than or equal to 25 °/s (turning), and the Pearson correlation coefficient was computed between the behavioral variable and the spike rate for these two conditions. Correlation coefficients (r values) are reported as mean ± SEM across flies and are displayed in the forward velocity figure panels (right). Paired t-tests were performed for stride length.

For Figure 5E, time points when each fly was turning ipsiversive to the recorded DN soma at 50–100 °/s were identified, and these time points were subdivided according to the fly’s forward movement, specifically focusing on forward deceleration (rate of change in forward velocity < −10 mm/s^2^) or forward acceleration (rate of change in forward velocity > 10 mm/s^2^). The mean normalized spike rate was then computed in the 100 ms prior to each of these timepoints. Only data from timepoints when the middle and hind legs were stepping forward were included. Data were interpolated to 250 Hz. Both the mean normalized spike rate and the difference between the increasing and decreasing forward velocity conditions were plotted. Paired t-tests were performed for each DN comparing the mean normalized spike rate between deceleration and acceleration conditions for each DN. A two-sample t-test was performed to determine if this comparison was different for DNa02 and DNg13.

For Figure S7A, the mean spike rate was computed for each fly when it was not walking versus walking; during walking epochs, we required that any nonzero rotational velocity values were in the contraversive direction (i.e. rotational velocity < 0 °/s). Paired t-tests were performed.

For Figure S7B, the Pearson correlation coefficient was computed between each pair of behavioral variables that was plotted in Figures 5C and 5D. Only data from times when the middle and hind legs were walking forwards were included. The correlation was computed for each fly, and the mean across flies was reported.

#### Data analysis - Figure 6

For Figure 6A, the linear filters relating rotational velocity or stride length (ipsilateral for DNa02 and contralateral for DNg13) with DN spike rate were computed by first computing the cross-correlation between the behavioral variable and the spike rate as well as the autocorrelation of the spike rate, transforming them into the frequency domain, dividing them, low pass filtering them, and then transforming them back into the time domain. This effectively computes the first-order Wiener kernel. Only data from times when the middle and hind legs were stepping forward were included. The cross-correlation and the autocorrelation were computed by calculating the Pearson correlation coefficient at every time lag between −1 and 1 s, in 1 ms increments. The data was low pass filtered according to c(ω) = ê(−abs(ω − f_cut_)/f_τ_) for abs(ω) ≥ f_cut_. f_cut_ was 2 Hz and f_τ_ was 0.5 Hz. Cells for which the cross-correlation did not have a defined peak were excluded, as they did not produce meaningful filters; four DNg13 cells were excluded for this reason. The filter relating the ipsilateral stride length and DNa02 spike rate was sign-inverted to make comparison with the DNg13 filter easier. The peak time was the time at which the maximum in the filter occurred. One-sample t-tests were performed to determine whether the peak times were significantly different from 0.

Figure 6B plots the autocorrelation for DNa02 and DNg13 spike rate. Only data from times when the middle and hind legs were stepping forward were included. The full-width at half-maximum of the autocorrelation function for each fly was determined by finding the time window when the autocorrelation was greater than or equal to 0.5. A two-sample t-test was performed to determine whether the difference in the full-width at half-maximum between DNa02 and DNg13 was significant.

For Figure 6C, turning bouts were identified in the freely walking fly data as described for Figure 1 and S1. Bouts were included if they had an initial forward velocity of 10–15 mm/s, a peak rotational velocity of 50–150 °/s, and a duration of 0.3–0.7 s. The continuous estimate of stride length from 0.5 s before the peak in rotational velocity to 0.5 s after the peak in rotational velocity was extracted for each leg in each bout; this was interpolated to 100 Hz. This was then normalized by subtracting the minimum stride length during the snippet and dividing by the range (max − min). The mean across bouts was then computed for each leg. For plotting, the mean was again normalized to 0–1 by subtracting the minimum and dividing by the range. The full-width at half-maximum was computed on the normalized stride length for each bout. The maximum was found in the stride length within ±0.2 s of the peak in rotational velocity, and the duration that the stride length spent greater than the half-max value was determined.

In Figure 6D, the phase of the stride cycle was computed as the average phase across all six legs, with the phases of all the legs aligned by subtracting the mean offset relative to a reference leg (ipsilateral middle leg for DNa02 and contralateral middle leg for DNg13). In the bottom plots, each spike was assigned to a phase bin (72 bins) based on the phase 100 ms prior, which was the offset that approximately maximized the degree of phase locking in DNa02 (DNg13 spikes were not phase locked, regardless of the offset used). Spike counts per bin were normalized by the time spent at each phase and then expressed as the difference from the mean across all phases. All spikes when the fly was walking were used.

#### Data analysis - Figures 7 and S4E–I

In Figures 7A and 7B, monosynaptic outputs of DNa02 and DNg13 were grouped by category; like for Figure 3B, each neuron was treated equally, and the plot expresses category membership as the percent of the total number of output neurons in the leg neuromeres. In Figures S4H and S4I, the plots on the left are similar, except that Figure S4H categorizes all output neurons, and Figure S4I looks at outputs in the dorsal neuropil regions, specifically. Figures S4E and S4F and the right plots in Figures S4H and S4I sum the number of synapses made by the neurons in each category and express them as a percent of the total number of synapses (like Figure S4C).

Figure 7C displays the number of neurons that are postsynaptic to DNa02 alone, DNg13 alone, or shared between the two for each hemisphere. Figure S4G shows the total number of synapses made onto neurons in each of those categories.

Figure 7D shows the monosynaptic and disynaptic connections between DNa02/DNg13 and the T1 leg motor neurons (minimum number of synapses = 15). The graphs for DNa02 and DNg13 are plotted separately, so there are motor neurons that are repeated between the two. Connections between premotor neurons are not displayed. Leg motor neurons are labeled based on their inferred effect on the leg^27^. Neurotransmitter labels reflect machine vision predictions^71^ (for DNs) or hemilineage membership^72,127,128^ (for premotor cells). Neurotransmitters were deemed excitatory or inhibitory based on the effect of their ionotropic receptors; glutamate was deemed inhibitory as the inhibitory glutamate-gated chloride channel GluClɑ^132,133^ is widely expressed in the ventral nerve cord^134^.

Figures 7E and 7F show the premotor and motor neurons downstream of DNg13 that were hypothesized to be active during the power (Figure 7E) and return (Figure 7F) phases of the stride cycle. Neurons were hypothesized to be active if their inferred effect on the leg (accounting for the sign of the connections) was consistent with the results of DNg13 perturbation (Figure 4) during that phase.

### Quantification and statistical analysis

All statistical methods used in the paper are described briefly in the figure legends and in detail in the Method details. The p-values for all statistical tests are in Table S1. Statistical significance was defined at ɑ = 0.05. When appropriate, Holm-Bonferroni correction was applied for multiple comparisons. Definitions of sample size, measures of center and dispersion, and precision measures are also indicated in figure legends and Method details. Statistics were computed using MATLAB.

## Supplementary Material

Supplementary Figure 1 Figure S1. Modulations of step parameters during steering in freely walking flies, related to Figure 1 (A) *Top*: schematic of the walking features tracked with a camera from above: the velocity in three body axes and the positions of the tips of the 6 legs. *Bottom:* during a step cycle, the tip of the leg moves posterior relative to the body and then anterior, defining the anterior and posterior extreme positions (AEP and PEP). The stride length is the anterior-posterior distance between these two positions and the step direction is the angle of the vector from the AEP to the PEP.
(B) When flies steer, they modulate the step direction of the front and middle legs. Positive differences in step direction are when the power stroke brings the leg tip further toward the outside; negative differences are when the power stroke brings the leg tip further toward the inside. Asterisks mark legs with significant differences. For each turning bout, we measured the bout’s peak rotational velocity, as well as the step direction for the step that occurred at that peak. Step directions are expressed as the change from the mean when the fly was not turning. Violin plots are Gaussian kernel estimates fit to all data points. n = 138 (55), 454 (68), 434 (68), and 151 (50) bouts (flies) for 20–50, 50–100, 100–150, and 150–200 °/s, respectively.
(C) Pivots and swerves differentially modulate the return stroke position, the power stroke position, and the step direction. The return and power stroke positions are plotted as positions in the anterior-posterior axis, and step direction is plotted as the difference from the mean when the fly was not turning. n = 389 (64) and 155 (53) bouts (flies) for pivots and swerves, respectively.
See Table S1 for statistics.

Supplementary Figure 2 Figure S2. Correlations between the activity of all DN types imaged and walking, related to Figure 2 Each DN type imaged is represented by a single right-left pair of neurons. The three plots for each cell type are: *(A and D)* rotational velocity vs. the right cell – left cell difference in ΔF/F. Individual flies are in gray, and the mean across flies is in black. *(B and E)* forward velocity vs. the right cell + left cell sum in ΔF/F. *(C and F)* the mean right + left sum in ΔF/F during time periods when the fly is walking or not walking. The mean activity is higher when the fly was walking than when it was not walking for many DN types. Asterisks mark significant differences. Dots are individual flies, and the horizontal line is the mean across flies. Gray lines connect points from the same fly. n=10 flies for DNa01; n=8 flies for DNa02; n=7 flies for DNb05; n=7 flies for DNb06; n=5 flies for DNg13; n=6 flies for DNg14; n=3 flies for DNg15; n=7 flies for DNg16; n=7 flies for DNg31; n=14 flies for DNg34; n=5 flies for DNp05; n=4 flies for DNp09; n=5 flies for DNp11; n=5 flies for DNp12; n=5 flies for DNp18; n=4 flies for DNp32.
See Table S1 for statistics.

Supplementary Figure 3 Figure S3. Example DN calcium responses during walking, related to Figure 2 For each DN type, from *top* to *bottom*, left cell ΔF/F, right cell ΔF/F, the fly’s rotational velocity, and the fly’s forward velocity, during an example 20-s period. (A) DNg34, (B) DNa02, (C) DNa02, (D) DNb05, (E) DNb06, and (F) DNg13. In general, we observed that the signal-to-noise ratio in DN calcium signals was relatively low, compared to other neurons in the *Drosophila* brain.

Supplementary Figure 4 Figure S4. Steering descending neurons have distinct inputs and outputs, related to Figures 3 and 7 (A) Image shows the morphology of the left DNa02 and the right DNg13 in the brain, from the full female brain connectome^23,26^.
(B) The number of synapses made onto the DNs by their shared or unique presynaptic cells. For shared inputs, the number of synapses is colored based on the postsynaptic DN. Shared presynaptic cells contribute a smaller fraction of the DN’s inputs than their unique presynaptic partners. For comparison, Figure 3A shows the same connectivity data broken down by presynaptic neurons, rather than synapses.
(C) Stacked bar charts summarize the percent of synapses made by the cells in each category onto each DN in the brain, expressed as a percentage of all presynapses. Figure 3B shows the same connectivity data broken down by presynaptic neurons rather than synapses.
(D) Image shows the morphology of the DNa02 axon and the DNg13 axon on the left side of the ventral nerve cord^25,27^. These axons come from the DNa02 in the left hemisphere of the brain and the DNg13 in the right hemisphere of the brain.
(E) Output synapses from DNa02 and DNg13 onto cells in each category in the leg neuromeres of the ventral nerve cord, expressed as a percentage of all postsynapses made in the leg neuromeres. Note that DNa02 synapses onto neurons in the dorsal neuropils (associated with the wings, halteres, and neck), and those are excluded from this plot. For comparison, Figure 7A shows the same connectivity data broken down by postsynaptic neurons, rather than synapses.
(F) Synapses made onto neurons in each of the three leg neuromeres (T1, T2, T3), expressed as a percentage of all postsynapses made in the leg neuromeres. Intersegmental neurons are assigned to a segment based on their cell body locations. For comparison, Figure 7B shows the same connectivity data broken down by postsynaptic neurons, rather than synapses.
(G) The number of synapses made onto shared and unique postsynaptic targets of each DN. For shared targets, the number of synapses made is colored based on the presynaptic DN. DNa02 and DNg13 axons projecting to the left side of the ventral nerve cord make very few synapses onto the same cells. For comparison, Figure 7C shows the same connectivity data broken down by postsynaptic neurons, rather than synapses.
(H) *Left:* The percent of all monosynaptic output neurons in the ventral nerve cord that likely belong to leg or wing circuitry. *Right:* the fraction of synapses made onto leg or wing circuitry neurons, expressed as a percentage of the total synapses made by these DNs.
(I) Percent of neurons (*left*) and percent of synapses made onto neurons in each category (*right*) for the monosynaptic output neurons classified as belonging to the wing circuitry.

Supplementary Figure 5 Figure S5. Manipulations of DN spike rate, related to Figure 4 (A) Example recordings from DNa02 cells expressing CsChrimson (CsCh, *top*) and GtACR1 (*bottom*) through our genetic mosaic strategy. A light pulse (with onset and offset denoted by colored lines) depolarizes DNa02 and increases spiking when it expresses CsCh, whereas it hyperpolarizes DNa02 and suppresses spiking when it expresses GtACR1. Note that we performed these recordings in flies that were 7 days post-eclosion to match the behavioral experiments, and it is extremely challenging to obtain high-quality recordings in behaving flies at this age; consequently, the fly in the CsCh recording was not walking, explaining the lack of non-light-induced spiking, as DNa02 does not spike if the fly is still (see Figure S7A). The fly in the GtACR1 recording was walking, but the spikes are small because of poorer recording quality.
(B) A depolarizing current injection step (*top*) increases DNg13 spike rate, while a hyperpolarizing current step suppresses spiking (*bottom*). Colored lines mark the start and end of current injection. Note that this is an example recording from a fly in Figure 4.

Supplementary Figure 6 Figure S6. Perturbing steering-related descending neurons, related to Figure 4 (A) Effects of bilateral optogenetic activation of DNa02 with CsChrimson (CsCh). Dots are individual flies, lines are means across flies. Ipsi/contra were randomly assigned. The effect of optogenetic stimulation is significant for forward velocity but not rotational velocity. n=3 flies (CsCh+) and 7 flies (no CsCh). Box marks a significant change.
(B) Bilateral optogenetic activation changes the stride length across legs. Boxes around ipsi/contra mark significant effects ±CsCh in post-hoc tests considering all three legs on one side, while boxes around panels mark significant effects for that leg.
(C) Bilateral optogenetic activation of DNa02 shortens the return stroke but has little effect on the power stroke.
(D) Difference in rotational and forward velocity during bilateral optogenetic inhibition of DNa02 with GtACR1. Ipsi/contra were randomly assigned. Optogenetic inhibition has no significant effect. n=4 flies (GtACR1) and n=7 flies (no GtACR1).
(E) Bilateral inhibition of DNa02 has no significant effect on stride length.
(F) Bilateral optogenetic inhibition of DNa02 has little effect on the return or power stroke.
(G) *Left:* unilateral activation of DNa02 with optogenetic stimulation of CsCh produces changes in the step direction of all six legs.
*Middle*: unilateral optogenetic inhibition of DNa02 with GtACR1 affects step direction.
*Right:* unilateral hyperpolarization and depolarization of DNg13 produce changes in the step direction of the front and middle legs.
Changes in step direction are expressed as the difference from the step direction during matched periods without stimulation. Dots are individual flies, and the horizontal lines are the means across flies. Gray lines connect points from the same fly.
See Table S1 for statistics.

Supplementary Figure 7 Figure S7. DNa02 and DNg13 are not routinely recruited as speed-control DNs, related to Figure 5 (A) Mean DNa02/DNg13 spike rate when the fly was walking (and not making ipsiversive turns, i.e. any turning was in the contraversive direction) compared to epochs the fly was not walking. Dots are individual flies, and horizontal lines are the means across flies. Points from the same fly are connected with gray lines. Asterisks mark significant changes. n=4 cells in 4 flies for DNa02, and n=9 cells in 9 flies for DNg13.
(B) Many behavioral parameters are correlated during turning. Mean correlations (Pearson correlation coefficient) between behavioral parameters that were correlated with DNa02 and DNg13 spike rate, in the flies from Figure 5.
(C) *Left*: DNa02 spike rate is minimally correlated with forward velocity when flies are turning (rotational speed >25°/s) or not turning (rotational speed <25°/s).
*Right*: the correlation between ipsilateral stride length and normalized DNa02 spike rate only emerges when the fly is turning. Summary plot shows Pearson correlation coefficient between ipsilateral stride length and normalized DNa02 spike rate in both conditions; here, pairs of connected dots are flies, and horizontal lines are means across flies.
(D) *Left*: DNg13 spike rate is weakly positively correlated with forward velocity when flies are turning or not turning.
*Right*: correlation between contralateral stride length and normalized DNg13 spike rate is significantly weaker when flies are not turning.
(E) Difference in the position of the ipsilateral leg tips at the end of the return stroke (*left*) and the end of the power stroke (*right*) when comparing slow – fast forward velocity (< 8 mm/s vs. > 8 mm/s), optogenetic activation of DNa02 with CsChrimson (CsCh), and optogenetic silencing of DNa02 with GtACR1. Each dot is a fly, and x symbols are means across flies. Note that, when flies slow down spontaneously, their middle leg positions tend to shift medially^66^, and we also see the same medial shift for the front legs (black points). Activating DNa02 bilaterally slows down the fly, but it produces a lateral shift rather than a medial shift in the front and middle legs during the return stroke (red and yellow points). Points from slow – fast forward velocity are all DNa02 and DNg13 manipulation flies during time periods without stimulation and without turning (rotational speed <10°/s) (n=52 flies). n=7 flies (no CsCh), n=8 flies (unilateral CsCh), n=3 flies (bilateral CsCh), n=7 flies (no GtACR1), n=17 flies (unilateral GtACR1), n=4 flies (bilateral GtACR1).
(F) Difference in the position of the ipsilateral leg tips at the end of the return stroke (*left*) and the end of the power stroke (*right*) when comparing slow – fast forward velocity (< 8 mm/s vs. > 8 mm/s), unilateral silencing of DNg13 with hyperpolarizing current injection, and unilateral activation of DNg13 with depolarizing current injection. Changes in leg tip positions upon manipulation do not match the differences between slow and fast forward velocity, suggesting that DNg13 does not play a major role in forward speed control. n=6 flies for DNg13 current injection.

Table S1

## Figures and Tables

**Key resources table:** 

REAGENT or RESOURCE	SOURCE	IDENTIFIER
Chemicals, peptides, and recombinant proteins		
all-trans retinal	Sigma-Aldrich	CAS: 116-31-4
dispase	Worthington Biochemical	CAS: 9001-92-7
Experimental models: Organisms/strains		
*D. melanogaster*: isoD1 strain	Gift from T. Clandinin^105^	N/A
*D. melanogaster: P{y[+t7.7] w[+mC]=R22C05-p65.AD}attP40; P{y[+t7.7] w[+mC]=R56G08-GAL4.DBD}attP2*	Gift from G. Card^29^	Janelia line SS00731 RRID: BDSC_75862
*D. melanogaster: P{y[+t7.7] w[+mC]=R75C10-p65.AD}attP40; P{y[+t7.7] w[+mC]=R87D07-GAL4.DBD}attP2*	Gift from G. Card^29^	Janelia line SS00730
*D. melanogaster: P{y[+t7.7] w[+mC]=VT019391-p65.AD}attP40; P{y[+t7.7] w[+mC]=VT028198-GAL4.DBD}attP2*	Gift from G. Card^29^	Janelia line SS01051 RRID:BDSC_75921
*D. melanogaster: P{y[+t7.7] w[+mC]=R20C04-p65.AD}attP40; P{y[+t7.7] w[+mC]=VT025999-GAL4.DBD}attP2/TM6B, Tb[1]*	Gift from G. Card^29^	Janelia line SS02631 RRID: BDSC_75976
*D. melanogaster: P{y[+t7.7] w[+mC]=VT027166-p65.AD}attP40; P{y[+t7.7] w[+mC]=VT009857-GAL4.DBD}attP2*	Gift from G. Card^29^	Janelia line SS02259 RRID: BDSC_75873
*D. melanogaster: P{y[+t7.7] w[+mC]=R38H06-p65.AD}attP40; P{y[+t7.7] w[+mC]=VT018689-GAL4.DBD}attP2*	Gift from G. Card^29^	Janelia line SS01069 RRID: BDSC_75828
*D. melanogaster: P{y[+t7.7] w[+mC]=VT043400-p65.AD}attP40; P{y[+t7.7] w[+mC]=VT043662-GAL4.DBD}attP2/TM6B, Tb[1]*	Gift from G. Card^29^	Janelia line SS02377 RRID: BDSC_75874
*D. melanogaster: P{y[+t7.7] w[+mC]=VT019060-p65.AD}attP40; P{y[+t7.7] w[+mC]=VT003280-GAL4.DBD}attP2*	Gift from G. Card^29^	Janelia line SS00865 RRID: BDSC_75877
*D. melanogaster: P{y[+t7.7] w[+mC]=VT023490-p65.AD}attP40; P{y[+t7.7] w[+mC]=R38F04-GAL4.DBD}attP2*	Gift from G. Card^29^	Janelia line SS01540 RRID: BDSC_75903
*D. melanogaster: P{y[+t7.7] w[+mC]=VT025392-p65.AD}attP40; P{y[+t7.7] w[+mC]=VT057247-GAL4.DBD}attP2*	Gift from G. Card^29^	Janelia line SS02891 RRID: BDSC_76005
*D. melanogaster: P{y[+t7.7] w[+mC]=VT064490-p65.AD}attP40; P{y[+t7.7] w[+mC]=R69C11-GAL4.DBD}attP2/TM6B, Tb[1]*	Gift from G. Card^29^	Janelia line SS02392 RRID: BDSC_75878
*D. melanogaster: P{y[+t7.7] w[+mC]=R61H01-p65.AD}attP40; P{y[+t7.7] w[+mC]=R82C10-GAL4.DBD}attP2/TM6B, Tb[1]*	Gift from G. Card^29^	Janelia line SS02394 RRID: BDSC_75961
*D. melanogaster: P{y[+t7.7] w[+mC]=VT028153-GAL4}attP2*	Vienna Drosophila Resource Center	RRID:Flybase_FBst0486174
*D. melanogaster: P{y[+t7.7] w[+mC]=GMR55D12-GAL4}attP2*	Bloomington Drosophila Stock Center	RRID: BDSC_39430
*D. melanogaster: P{y[+t7.7] w[+mC]=GMR83A12-GAL4}attP2*	Bloomington Drosophila Stock Center	RRID: BDSC_40348
*D. melanogaster: P{y[+t7.7] w[+mC]=GMR47E06-GAL4}attP2*	Bloomington Drosophila Stock Center	RRID: BDSC_50313
*D. melanogaster: PBac{y[+mDint2] w[+mC]=20XUAS-IVS-jGCaMP7s} VK00005*	Bloomington Drosophila Stock Center^106^	RRID: BDSC_79032
*D. melanogaster: PBac{y[+mDint2] w[+mC]=20XUAS-IVS-CyRFP1}VK00037*	Gift from T. Clandinin; flies are not previously published but see ref.^107^ for CyRFP1	N/A
*D. melanogaster: P{y[+t7.7] w[+mC]=20XUAS-IVS-mCD8::GFP}attP40*	Gift from B. Pfeiffer and G. Rubin^108^	N/A
*D. melanogaster: TI{20XUAS-SPARC2-D-Syn21-CsChrimson::tdTomato-3.1}CR-P40*	Bloomington Drosophila Stock Center^36^	RRID: BDSC_84143
*D. melanogaster: P{y[+t7.7] w[+mC]=nSyb-IVS-phiC31}attP18*	Gift from T. Clandinin^36^	RRID: BDSC_84150
*D. melanogaster: norpA[P24]*	Gift from T. Clandinin^109^	FlyBase: FBal0013129
*D. melanogaster: TI{20XUAS-SPARC2-D-GtA CR1::eYFP}CR-P40*	This study	N/A
Software and algorithms		
FlyVR	T. Clandinin lab^60^	github.com/ClandininLab/FlyVR
Animal Part Tracker (APT)	K. Branson lab^61^	github.com/kristinbranson/APT
FicTrac	Moore et al. 2014^110^	rjdmoore.net/fictrac
SpinView 2.5.0.80	FLIR Systems, Inc.	www.flir.com/products/spinnaker-sdk
NoRMCorre	Pnevmatikakis and Giovannucci 2017^111^	github.com/flatironinstitute/NoRMCorre
ScanImage 2018	MBF Bioscience	archive.scanimage.org/SI2018/index.html
natverse	Bates et al. 2020^112^	natverse.org
fancr package	G. Jefferis lab	github.com/flyconnectome/fancr
BrainCircuits.io	S. Gerhard	braincircuits.io
CircStat Toolbox	Berens 2009^113^	www.mathworks.com/matlabcentral/fileexchange/10676-circular-statistics-toolbox-directional-statistics
Violin Plot	MATLAB Central File Exchange	www.mathworks.com/matlabcentral/fileexchange/45134-violin-plot
analysis code for this paper	This paper	doi.org/10.5281/zenodo.12775493
MATLAB 2018a, 2020b, and 2021b	MathWorks	RRID: SCR_001622
R version 4.2.2	R Project for Statistical Computing	RRID:SCR_001905
Psychtoolbox-3	refs^114,115^	http://psychtoolbox.org/
Other		
matte black spray paint	Grainger	Tough Guy 4WGC1
302 stainless steel foil with etched teardrop-shaped hole	Etchit	github.com/wilson-lab/design-files
UV-cured glue	Henkel Adhesives	Loctite AA 3972
UV gun	Electro-Lite Co.	LED-200
In-line heater	Warner Instrument Corporation	TC-324
¼”-diameter acrylic sphere	McMaster-Carr	1383K52
900-nm long-pass ink	Epolin	Spectre 120
far-red fluorescent ink	LDP LLC/maxmax.com	IR Ink 1
clear acrylic paint	Liquitex	Slow-Dri Blending Medium
780 nm LED	Thorlabs	M780L3
769/41-nm bandpass filter	Semrock	FF01-769/41
940 nm LED	Thorlabs	M940L3
950 nm longpass filter	Thorlabs	FELH0950
FicTrac camera	FLIR	GS3-U3-51S5M
FicTrac camera lens	Computar	M7528-MP
832/37 nm bandpass filter	Semrock	FF01-832/37
USB DAQ	Measurement Computing	USB-3101
NI DAQ, 16-bit A/D converter	National Instruments	NI PXIe-6361
leg camera	FLIR	GS3-U3-41C6NIR
leg camera lens	Computar	MLM3X-MP
silver mirror	Thorlabs	CCM1-P01
Ti:sapphire laser	Spectra-Physics	Mai-Tai HP with a DeepSee precompensation unit
20× water-dipping objective for two-photon imaging	Olympus	20×/NA1.0 XLUMPLFLN
25× water-dipping objective for two-photon imaging	Nikon	25×/NA1.1 N25×-APO-MP
photomultiplier tube	Hamamatsu	H10770PA-40 SEL
photomultiplier tube	Hamamatsu	R9110
525/50-nm bandpass filter	Semrock	FF01-525/50-50
629/53-nm bandpass filter	Semrock	FF01-629/56-50
galvo-galvo system	Cambridge Technology	6210HSM40
mercury lamp	Olympus	U-LH100HG
620/60 nm bandpass filter	Chroma	49019
5× objective	Olympus	5×/NA0.15 MPlan FL N
TRITC/Cy3 longpass filter cube	Chroma	19004
520/44-nm bandpass filter	Semrock	FF01-520/44-25
561 longpass filter	Semrock	LP02-561RU-25
561 dichroic	Semrock	Di02-R561-25x36
8x8 pixel blue LED	Dongguan Houjie Keming Electronic Factory	KEM-12088-AB
borosilicate glass (O.D. 1.5 mm, I.D. 0.86 mm)	Sutter	BF150-86-10HP
Flaming/Brown micropipette puller	Sutter	Model P-97
DIY Cerna body	Thorlabs	CEA1500
trinoculars and epifluorescence illumination optical path	Olympus	BX51W1
camera for electrophysiology	FLIR	GS3-U3-41C6NIR
470 nm LED	Thorlabs	M470L2-C1
FITC/EGFP longpass filter cube	Chroma	49012
borosilicate glass (O.D. 1.5mm, I.D. 1.1mm)	Sutter	B150-110-10HP
Axopatch 200B amplifier	Molecular Devices	Axopatch 200B
CV-203BU headstage	Molecular Devices	CV-203BU
