# Supplementary figures and images for: Fine-grained descending control of steering in walking Drosophila
Source: Cell. Author manuscript; Available in PMC 2026 Jan 7. (PMC12778575; doi:10.1016/j.cell.2024.08.033)

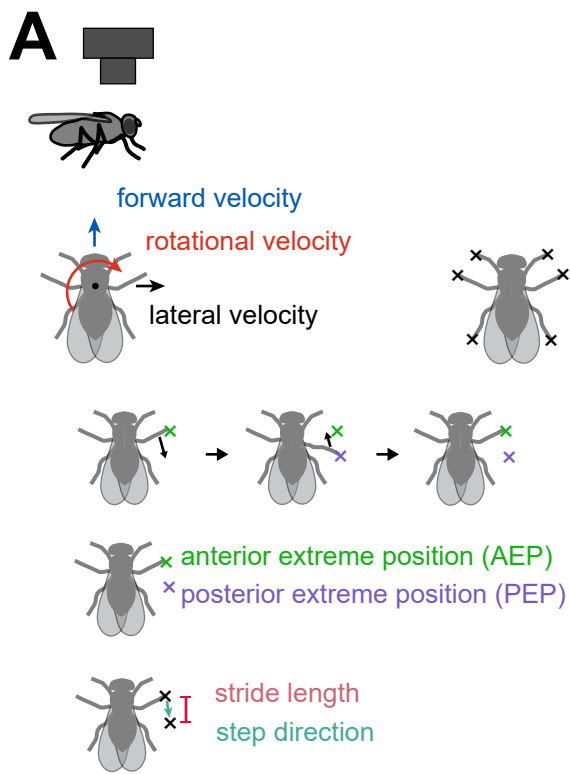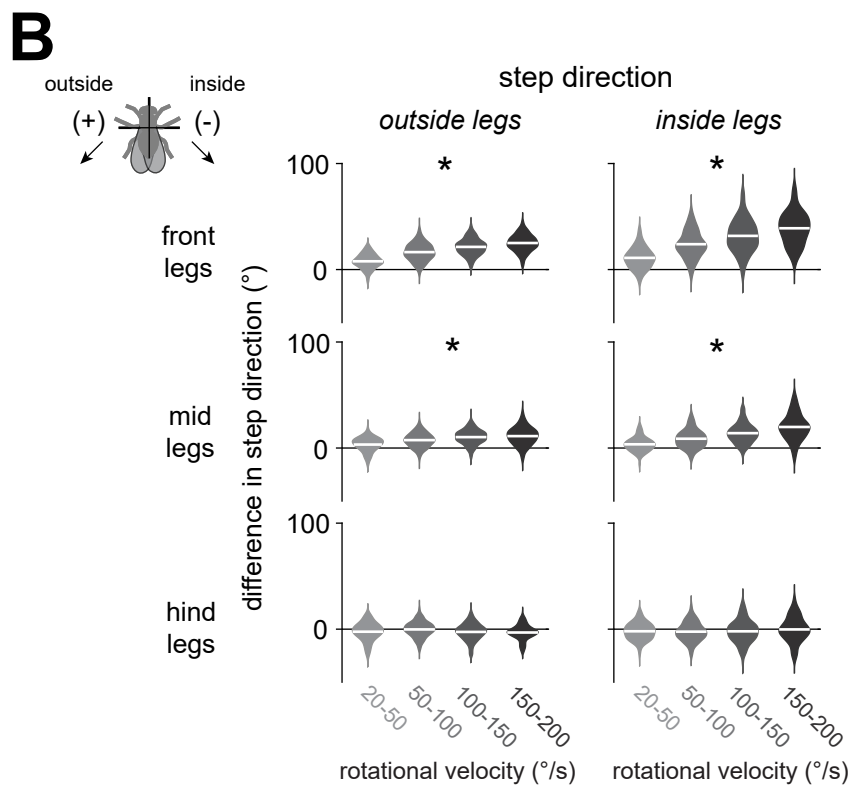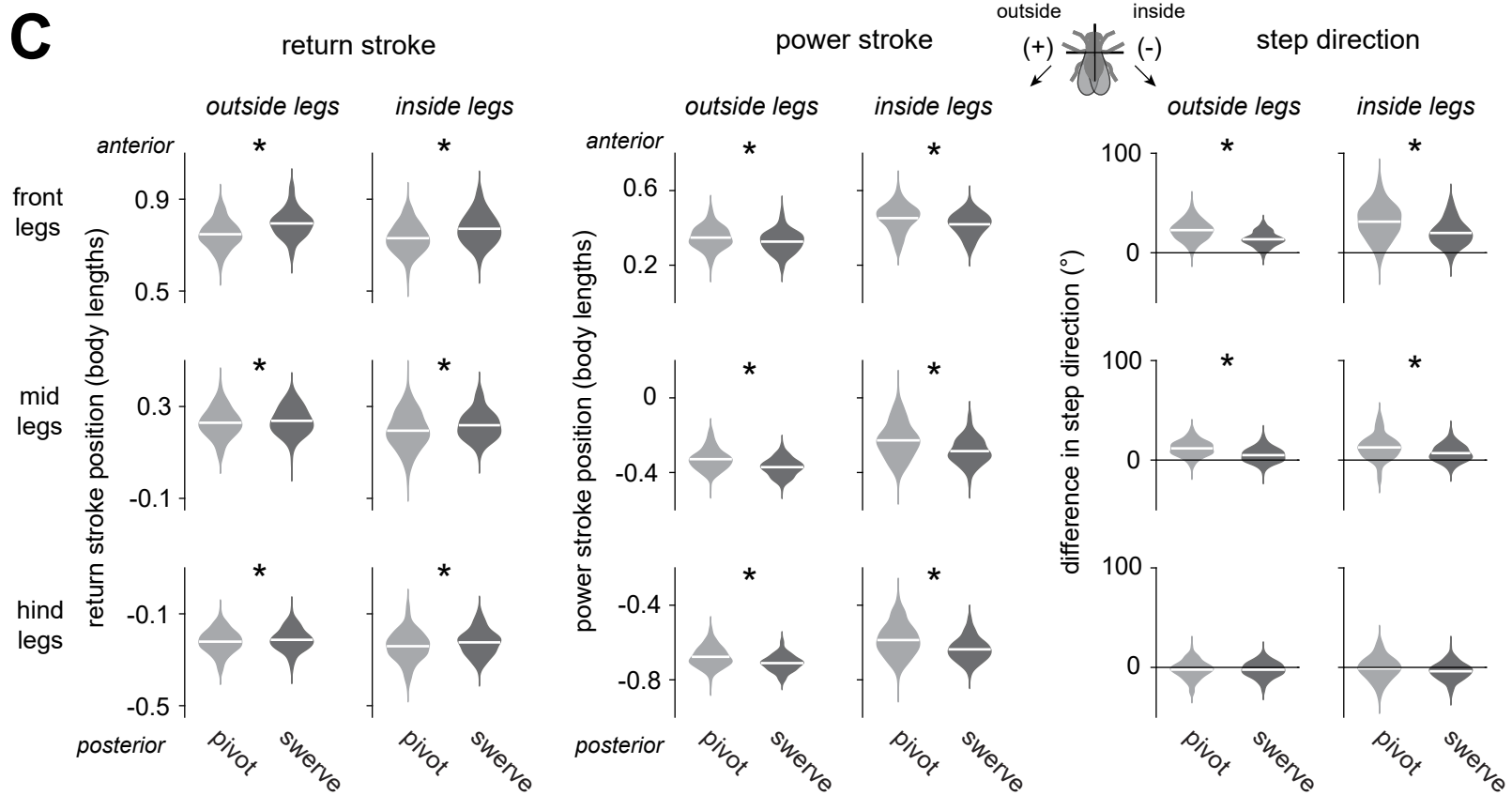

Supplement: Supplementary Figure 1 — (A) Top: schematic of the walking features tracked with a camera from above: the velocity in three body axes and the positions of the tips of the 6 legs. Bottom: during a step cycle, the tip of the leg moves posterior relative to the body and then anterior, defining the anterior and posterior extreme positions (AEP and PEP). The stride length is the anterior-posterior distance between these two positions and the step direction is the angle of the vector from the AEP to the PEP. (B) When flies steer, they modulate the step direction of the front and middle legs. Positive differences in step direction are when the power stroke brings the leg tip further toward the outside; negative differences are when the power stroke brings the leg tip further toward the inside. Asterisks mark legs with significant differences. For each turning bout, we measured the bout’s peak rotational velocity, as well as the step direction for the step that occurred at that peak. Step directions are expressed as the change from the mean when the fly was not turning. Violin plots are Gaussian kernel estimates fit to all data points. n = 138 (55), 454 (68), 434 (68), and 151 (50) bouts (flies) for 20–50, 50–100, 100–150, and 150–200 °/s, respectively. (C) Pivots and swerves differentially modulate the return stroke position, the power stroke position, and the step direction. The return and power stroke positions are plotted as positions in the anterior-posterior axis, and step direction is plotted as the difference from the mean when the fly was not turning. n = 389 (64) and 155 (53) bouts (flies) for pivots and swerves, respectively. See Table S1 for statistics. [file NIHMS2118063-supplement-Supplementary_Figure_1.pdf]

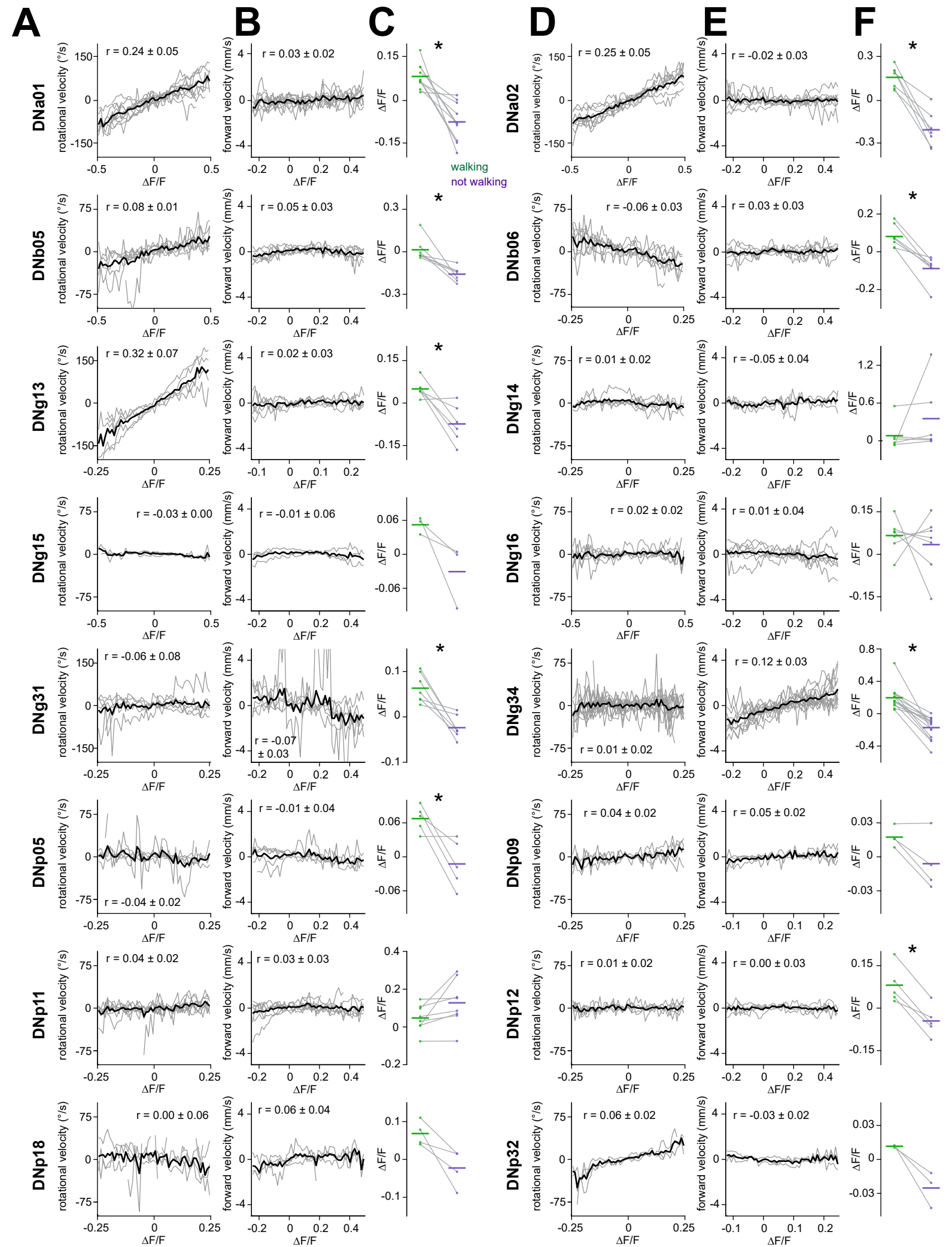

Supplement: Supplementary Figure 2 — Each DN type imaged is represented by a single right-left pair of neurons. The three plots for each cell type are: (A and D) rotational velocity vs. the right cell – left cell difference in ΔF/F. Individual flies are in gray, and the mean across flies is in black. (B and E) forward velocity vs. the right cell + left cell sum in ΔF/F. (C and F) the mean right + left sum in ΔF/F during time periods when the fly is walking or not walking. The mean activity is higher when the fly was walking than when it was not walking for many DN types. Asterisks mark significant differences. Dots are individual flies, and the horizontal line is the mean across flies. Gray lines connect points from the same fly. n=10 flies for DNa01; n=8 flies for DNa02; n=7 flies for DNb05; n=7 flies for DNb06; n=5 flies for DNg13; n=6 flies for DNg14; n=3 flies for DNg15; n=7 flies for DNg16; n=7 flies for DNg31; n=14 flies for DNg34; n=5 flies for DNp05; n=4 flies for DNp09; n=5 flies for DNp11; n=5 flies for DNp12; n=5 flies for DNp18; n=4 flies for DNp32. See Table S1 for statistics. [file NIHMS2118063-supplement-Supplementary_Figure_2.pdf]

## A DNg34

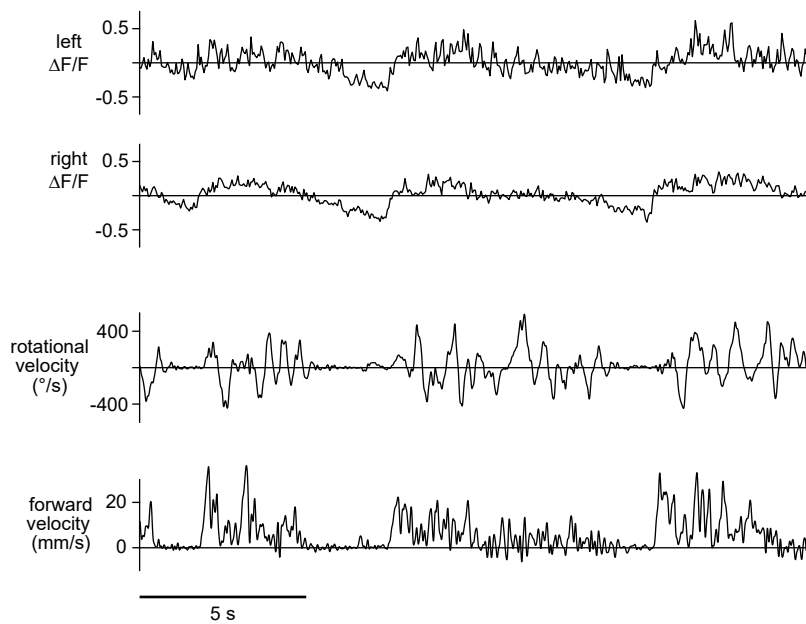

## B DNa01

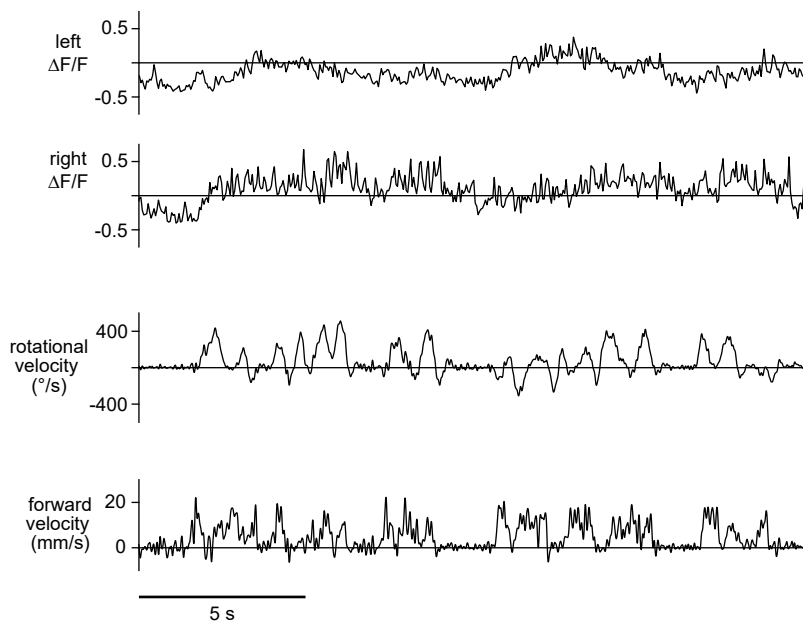

## C DNa02

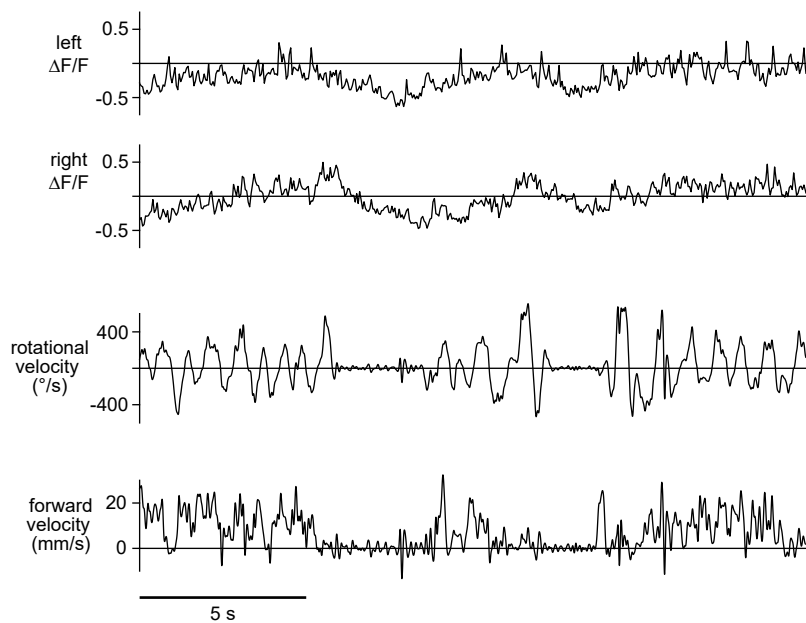

## D DNb05

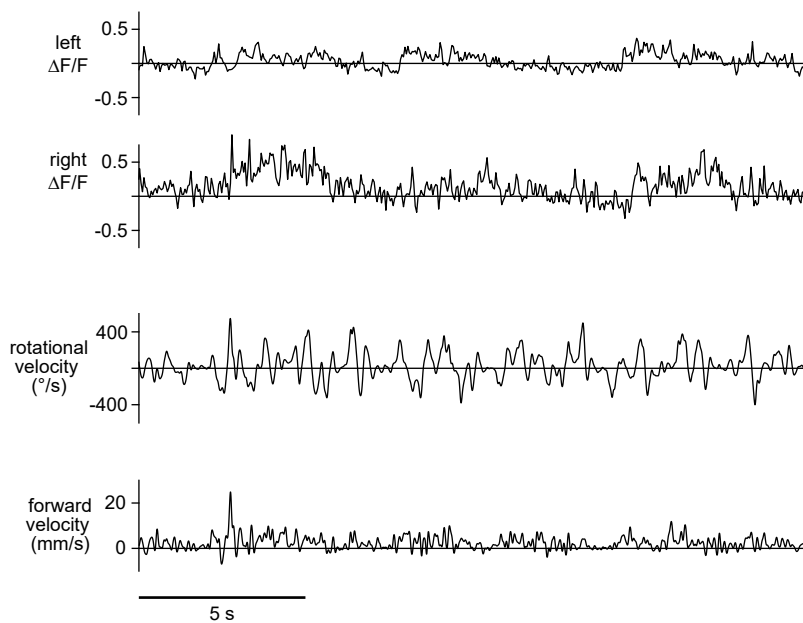

## E DNb06

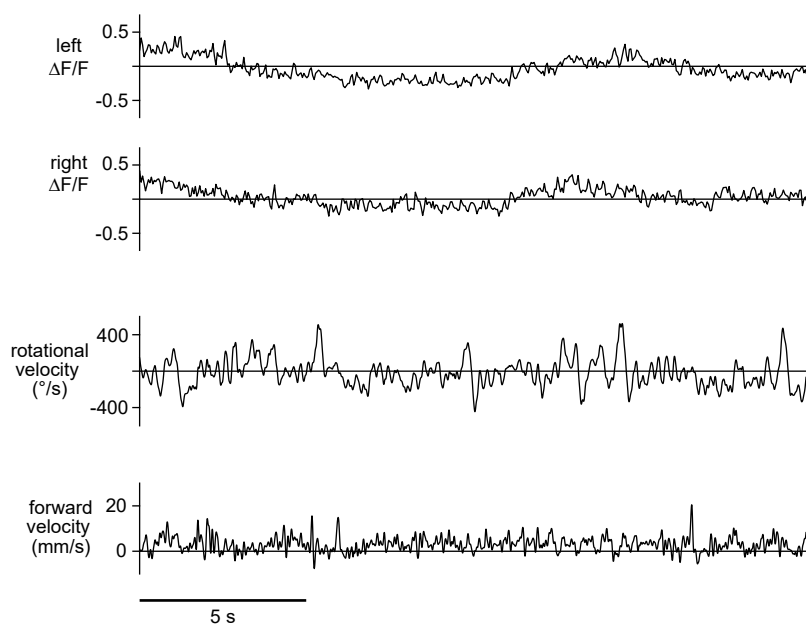

## F DNg13

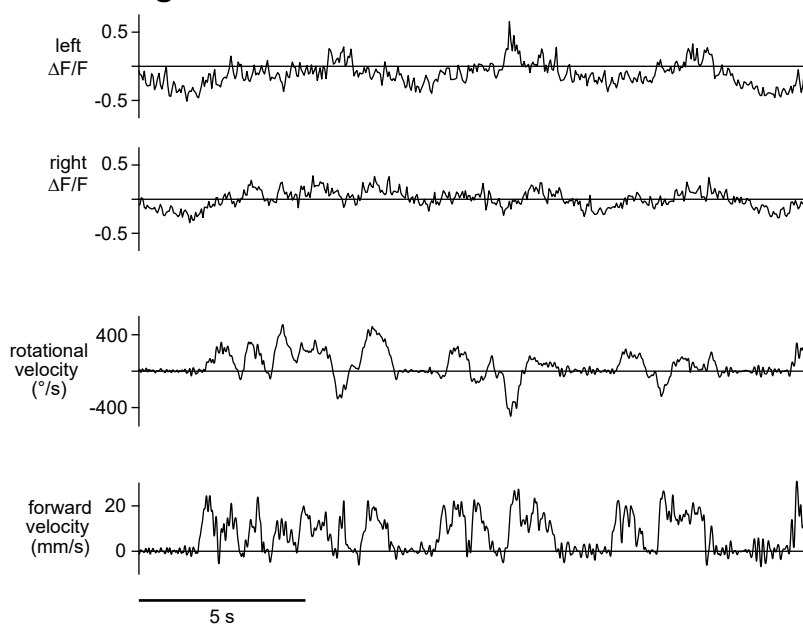

Supplement: Supplementary Figure 3 — For each DN type, from top to bottom, left cell ΔF/F, right cell ΔF/F, the fly’s rotational velocity, and the fly’s forward velocity, during an example 20-s period. (A) DNg34, (B) DNa02, (C) DNa02, (D) DNb05, (E) DNb06, and (F) DNg13. In general, we observed that the signal-to-noise ratio in DN calcium signals was relatively low, compared to other neurons in the Drosophila brain. [file NIHMS2118063-supplement-Supplementary_Figure_3.pdf]

**A**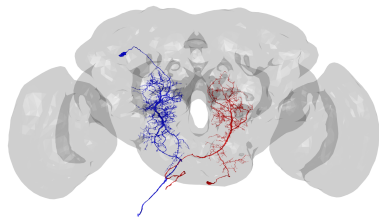**B**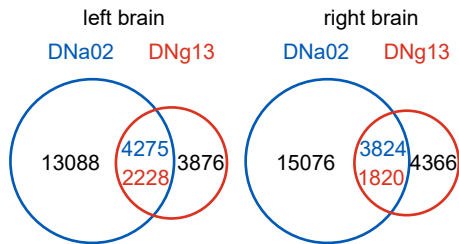**C**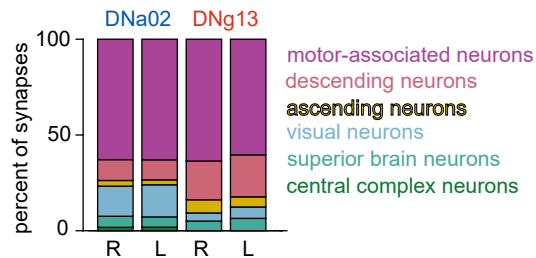**D**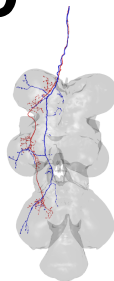**E**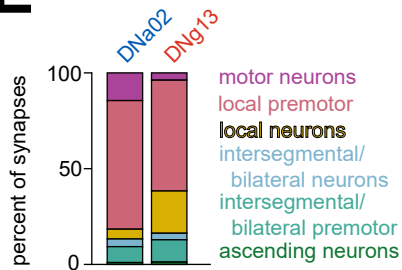**F**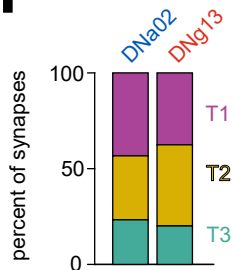**G**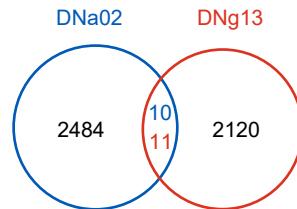**H**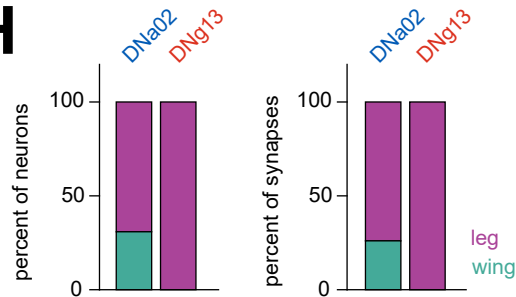**I**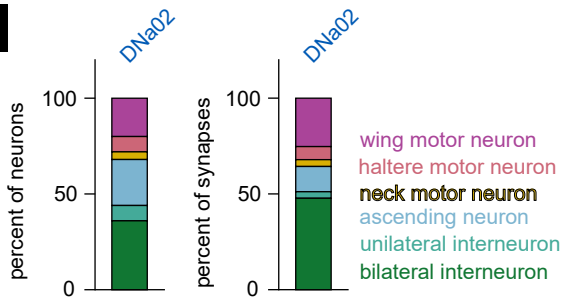

Supplement: Supplementary Figure 4 — (A) Image shows the morphology of the left DNa02 and the right DNg13 in the brain, from the full female brain connectome23,26. (B) The number of synapses made onto the DNs by their shared or unique presynaptic cells. For shared inputs, the number of synapses is colored based on the postsynaptic DN. Shared presynaptic cells contribute a smaller fraction of the DN’s inputs than their unique presynaptic partners. For comparison, Figure 3A shows the same connectivity data broken down by presynaptic neurons, rather than synapses. (C) Stacked bar charts summarize the percent of synapses made by the cells in each category onto each DN in the brain, expressed as a percentage of all presynapses. Figure 3B shows the same connectivity data broken down by presynaptic neurons rather than synapses. (D) Image shows the morphology of the DNa02 axon and the DNg13 axon on the left side of the ventral nerve cord25,27. These axons come from the DNa02 in the left hemisphere of the brain and the DNg13 in the right hemisphere of the brain. (E) Output synapses from DNa02 and DNg13 onto cells in each category in the leg neuromeres of the ventral nerve cord, expressed as a percentage of all postsynapses made in the leg neuromeres. Note that DNa02 synapses onto neurons in the dorsal neuropils (associated with the wings, halteres, and neck), and those are excluded from this plot. For comparison, Figure 7A shows the same connectivity data broken down by postsynaptic neurons, rather than synapses. (F) Synapses made onto neurons in each of the three leg neuromeres (T1, T2, T3), expressed as a percentage of all postsynapses made in the leg neuromeres. Intersegmental neurons are assigned to a segment based on their cell body locations. For comparison, Figure 7B shows the same connectivity data broken down by postsynaptic neurons, rather than synapses. (G) The number of synapses made onto shared and unique postsynaptic targets of each DN. For shared targets, the number of synapses made is colored ba [file NIHMS2118063-supplement-Supplementary_Figure_4.pdf]

**A****DNa02**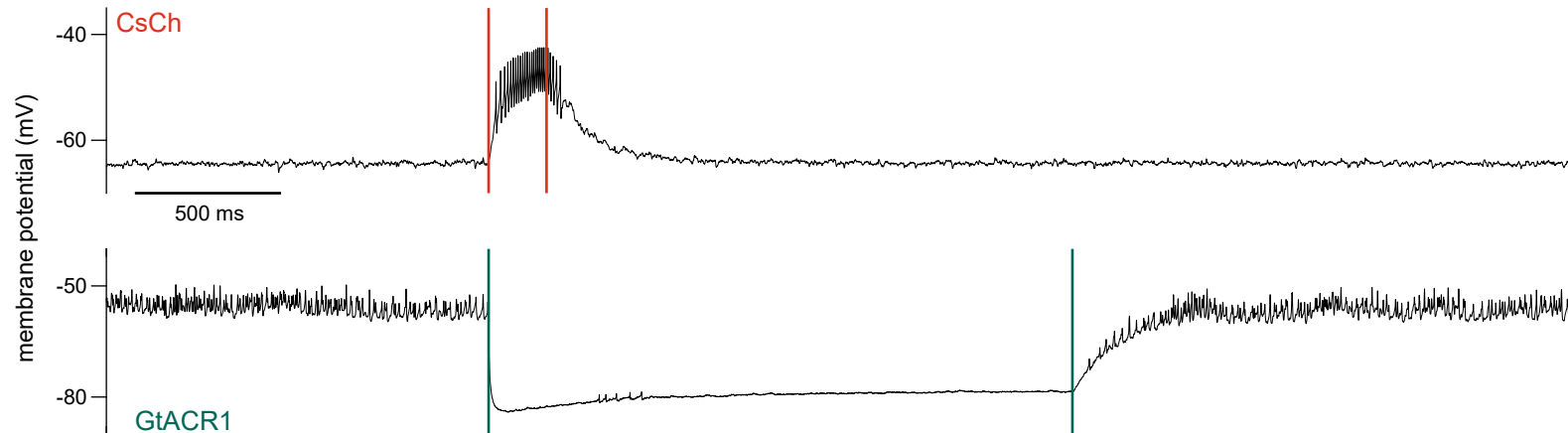**B****DNg13**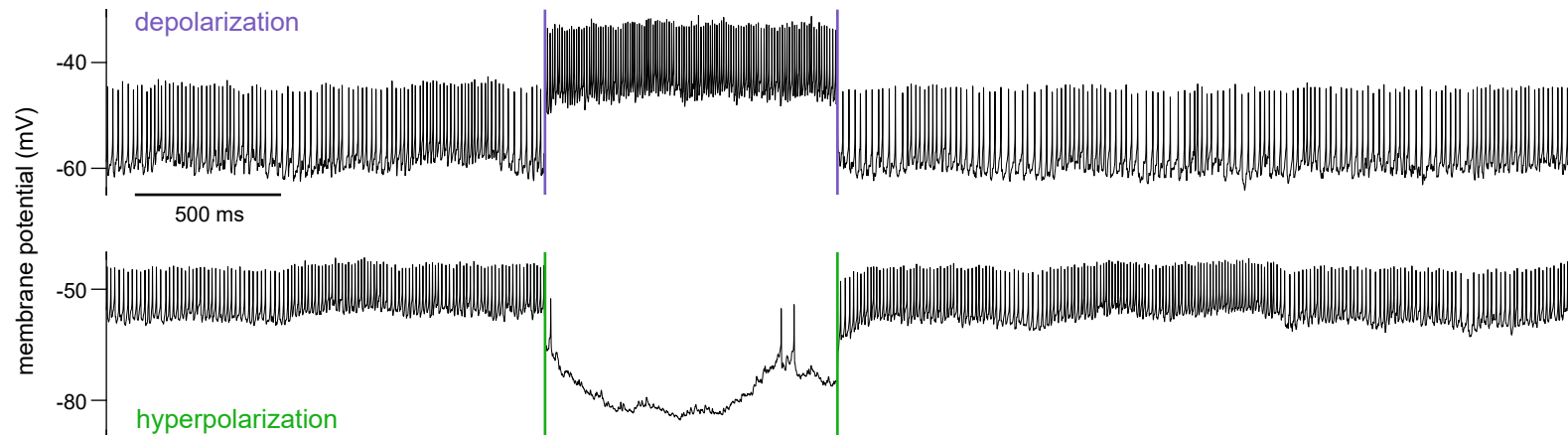

Supplement: Supplementary Figure 5 — (A) Example recordings from DNa02 cells expressing CsChrimson (CsCh, top) and GtACR1 (bottom) through our genetic mosaic strategy. A light pulse (with onset and offset denoted by colored lines) depolarizes DNa02 and increases spiking when it expresses CsCh, whereas it hyperpolarizes DNa02 and suppresses spiking when it expresses GtACR1. Note that we performed these recordings in flies that were 7 days post-eclosion to match the behavioral experiments, and it is extremely challenging to obtain high-quality recordings in behaving flies at this age; consequently, the fly in the CsCh recording was not walking, explaining the lack of non-light-induced spiking, as DNa02 does not spike if the fly is still (see Figure S7A). The fly in the GtACR1 recording was walking, but the spikes are small because of poorer recording quality. (B) A depolarizing current injection step (top) increases DNg13 spike rate, while a hyperpolarizing current step suppresses spiking (bottom). Colored lines mark the start and end of current injection. Note that this is an example recording from a fly in Figure 4. [file NIHMS2118063-supplement-Supplementary_Figure_5.pdf]

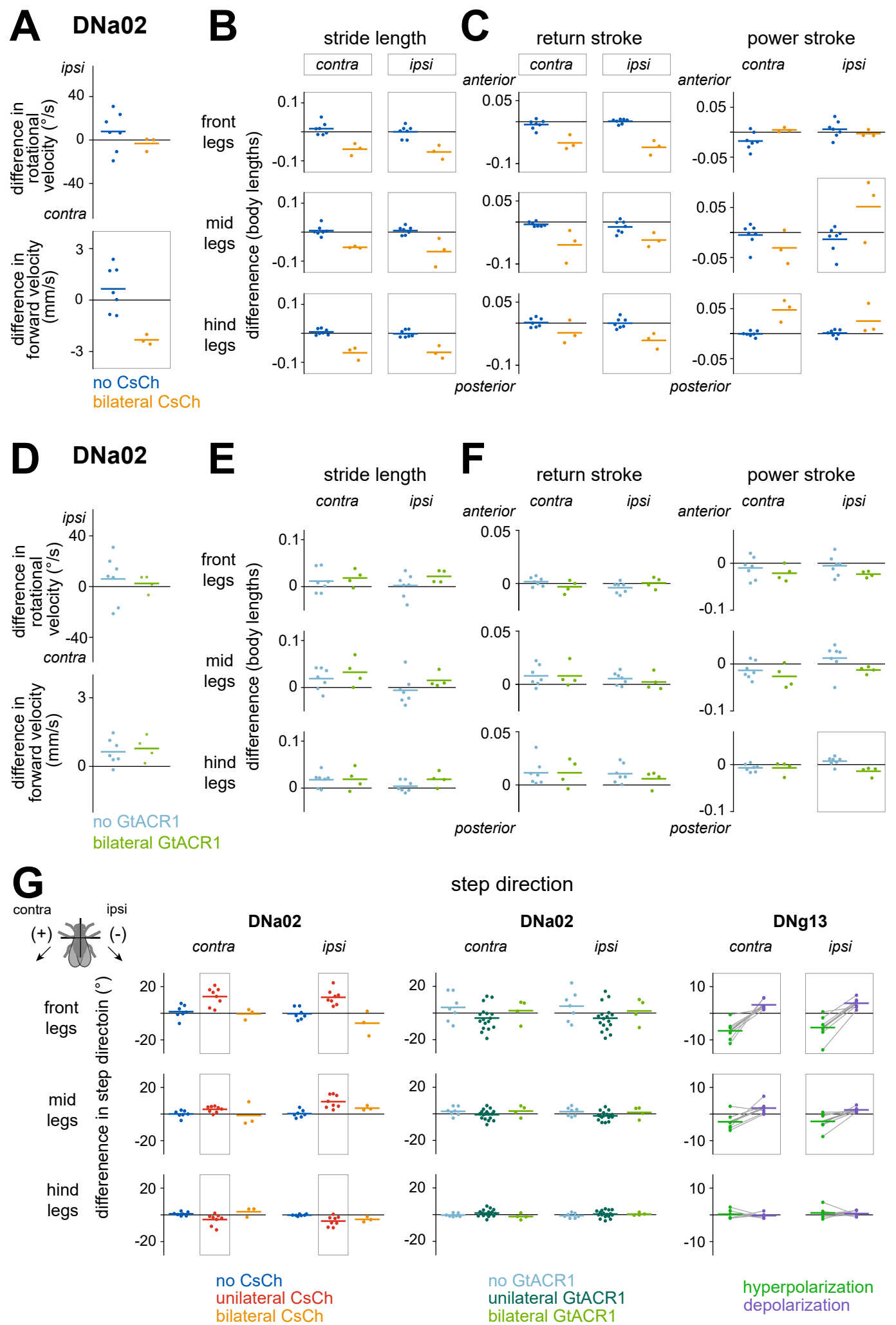

Supplement: Supplementary Figure 6 — (A) Effects of bilateral optogenetic activation of DNa02 with CsChrimson (CsCh). Dots are individual flies, lines are means across flies. Ipsi/contra were randomly assigned. The effect of optogenetic stimulation is significant for forward velocity but not rotational velocity. n=3 flies (CsCh+) and 7 flies (no CsCh). Box marks a significant change. (B) Bilateral optogenetic activation changes the stride length across legs. Boxes around ipsi/contra mark significant effects ±CsCh in post-hoc tests considering all three legs on one side, while boxes around panels mark significant effects for that leg. (C) Bilateral optogenetic activation of DNa02 shortens the return stroke but has little effect on the power stroke. (D) Difference in rotational and forward velocity during bilateral optogenetic inhibition of DNa02 with GtACR1. Ipsi/contra were randomly assigned. Optogenetic inhibition has no significant effect. n=4 flies (GtACR1) and n=7 flies (no GtACR1). (E) Bilateral inhibition of DNa02 has no significant effect on stride length. (F) Bilateral optogenetic inhibition of DNa02 has little effect on the return or power stroke. (G) Left: unilateral activation of DNa02 with optogenetic stimulation of CsCh produces changes in the step direction of all six legs. Middle: unilateral optogenetic inhibition of DNa02 with GtACR1 affects step direction. Right: unilateral hyperpolarization and depolarization of DNg13 produce changes in the step direction of the front and middle legs. Changes in step direction are expressed as the difference from the step direction during matched periods without stimulation. Dots are individual flies, and the horizontal lines are the means across flies. Gray lines connect points from the same fly. See Table S1 for statistics. [file NIHMS2118063-supplement-Supplementary_Figure_6.pdf]

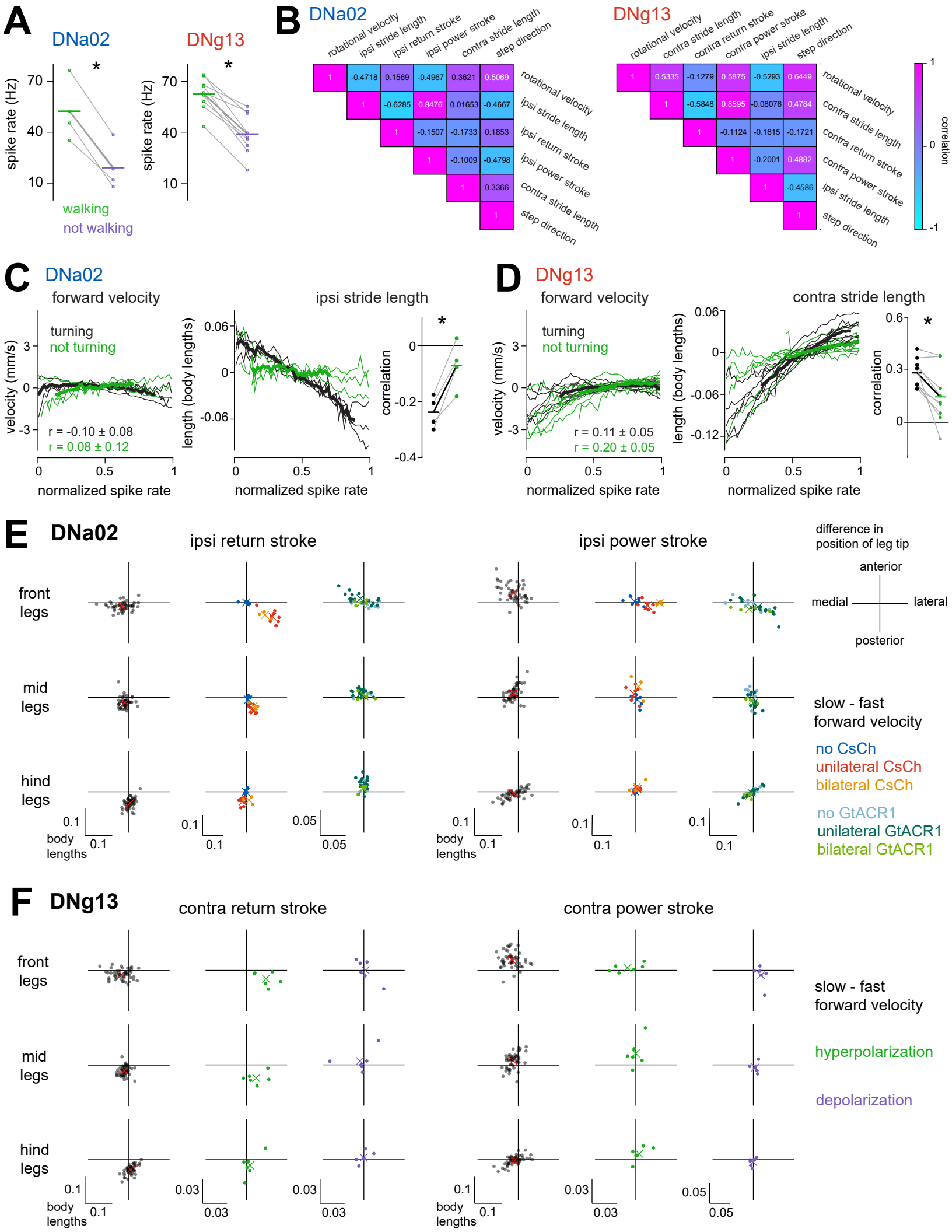

Supplement: Supplementary Figure 7 — (A) Mean DNa02/DNg13 spike rate when the fly was walking (and not making ipsiversive turns, i.e. any turning was in the contraversive direction) compared to epochs the fly was not walking. Dots are individual flies, and horizontal lines are the means across flies. Points from the same fly are connected with gray lines. Asterisks mark significant changes. n=4 cells in 4 flies for DNa02, and n=9 cells in 9 flies for DNg13. (B) Many behavioral parameters are correlated during turning. Mean correlations (Pearson correlation coefficient) between behavioral parameters that were correlated with DNa02 and DNg13 spike rate, in the flies from Figure 5. (C) Left: DNa02 spike rate is minimally correlated with forward velocity when flies are turning (rotational speed >25°/s) or not turning (rotational speed <25°/s). Right: the correlation between ipsilateral stride length and normalized DNa02 spike rate only emerges when the fly is turning. Summary plot shows Pearson correlation coefficient between ipsilateral stride length and normalized DNa02 spike rate in both conditions; here, pairs of connected dots are flies, and horizontal lines are means across flies. (D) Left: DNg13 spike rate is weakly positively correlated with forward velocity when flies are turning or not turning. Right: correlation between contralateral stride length and normalized DNg13 spike rate is significantly weaker when flies are not turning. (E) Difference in the position of the ipsilateral leg tips at the end of the return stroke (left) and the end of the power stroke (right) when comparing slow – fast forward velocity (< 8 mm/s vs. > 8 mm/s), optogenetic activation of DNa02 with CsChrimson (CsCh), and optogenetic silencing of DNa02 with GtACR1. Each dot is a fly, and x symbols are means across flies. Note that, when flies slow down spontaneously, their middle leg positions tend to shift medially66, and we also see the same medial shift for the front legs (black points). Activating DNa02 bilaterally slows [file NIHMS2118063-supplement-Supplementary_Figure_7.pdf]
