# Supplementary material for: Fine-grained descending control of steering in walking Drosophila
Source: Cell. Author manuscript; Available in PMC 2026 Jan 7. (PMC12778575; doi:10.1016/j.cell.2024.08.033)
Supplement: Table S1 [file NIHMS2118063-supplement-Table_S1.pdf]

Table S1. Statistics, related to Figures 1, 4, 5, 6, S1, S2, S6, and S7

Figure	Test	p-values
Figure 1C	Two-way ANOVA with legs (outside front/mid/hind, inside front/mid/hind) and rotational velocity as factors	rotational velocity: $p = 4.87\times10^{-77}$; leg identity: p = 0; interaction between leg identity and rotational velocity: p = 0
Figure 1C	Post-hoc Tukey-Kramer tests against reference for each rotational velocity (20-50°/s, 50-100°/s, 100-150°/s, 150-200°/s)	In ascending order of rotational velocity: out-front [0.0734, 6.67×10^{-12}, 0, 1.47×10^{-8}]; out-mid [0.0409, 7.15×10^{-18}, 0, 0]; out-hind [0.807, 1.68×10^{-4}, 0.00437, 7.18×10^{-10}]; in-front [0.00167, 0, 0, 0]; in-mid [1.00, 0, 0, 0]; in-hind [0.627, 0, 0, 0]
Figure 1E	Two-way ANOVA with legs (outside front/mid/hind, inside front/mid/hind) and pivot/swerve as factors	pivot/swerve: $p = 6.11\times10^{-31}$; leg identity: $p = 4.96\times10^{-66}$; interaction: p = 0.738
Figure 1E	Post-hoc Tukey-Kramer tests comparing pivot and swerve for each leg	out-front: $p = 4.28\times10^{-8}$; out-mid: p = 0.000744; out-hind: p = 0.000200; in-front: p = 0.000243; in-mid: $p = 5.29\times10^{-5}$; in-hind: p = 0.00524
Figure 4A	Two-sample t-tests comparing CsCh+ and no-CsCh	rotational velocity: p = 0.00141; forward velocity: p = 0.802
Figure 4B	Mixed ANOVA with legs (front/mid/hind) and side (ipsi/contra) as within-subjects variables and CsCh expression as the factor	±CsCh: p = 0.00650; legs: p = 0.713; side: $p = 2.01\times10^{-4}$; interaction between legs and ±CsCh: p = 0.808; interaction between side and ±CsCh: $p = 6.43\times10^{-4}$
Figure 4B	Post-hoc Tukey-Kramer tests comparing CsCh+ and no-CsCh for each side	ipsi: $p = 1.84\times10^{-4}$; contra: p = 0.126
Figure 4B	Mixed ANOVA with leg identity (ipsi front/mid/hind, contra front/mid/hind) as the within-subjects variable and CsCh expression as the factor	±CsCh: p = 0.00650; legs: $p = 5.87\times10^{-10}$; interaction between legs and ±CsCh: $p = 1.53\times10^{-8}$
Figure 4B	Post-hoc Tukey-Kramer tests comparing CsCh+ and no-CsCh for each leg	c-front: p = 0.915; c-mid: p = 0.0474; c-hind: p = 0.251; i-front: p = 0.0114; i-mid: $p = 6.40\times10^{-5}$; i-hind: $p = 1.58\times10^{-5}$.
Figure 4C (left)	Mixed ANOVA with legs (front/mid/hind) and side (ipsi/contra) as within-subjects variables and CsCh expression as the factor	±CsCh: $p = 4.41\times10^{-4}$; legs: p = 0.399; side: $p = 4.62\times10^{-5}$; interaction between legs and ±CsCh: p = 0.515; interaction between side and ±CsCh: $p = 4.61\times10^{-5}$
Figure 4C (left)	Post-hoc Tukey-Kramer tests comparing CsCh+ and no-CsCh for each side	ipsi: $p = 3.95\times10^{-6}$; contra: p = 0.127
Figure 4C (left)	Mixed ANOVA with leg identity (ipsi front/mid/hind, contra front/mid/hind) as the within-subjects variable and CsCh expression as the factor	±CsCh: $p = 4.41\times10^{-4}$; legs: $p = 4.27\times10^{-8}$; interaction between legs and ±CsCh: $p = 3.62\times10^{-8}$
Figure 4C (left)	Post-hoc Tukey-Kramer tests comparing CsCh+ and no-CsCh for each leg	c-front: p = 0.0144; c-mid: p = 0.0818; c-hind: p = 0.774, i-front: $p = 9.25\times10^{-5}$; i-mid: $p = 1.43\times10^{-4}$; i-hind: $p = 6.59\times10^{-5}$
Figure 4C (right)	Mixed ANOVA with legs (front/mid/hind) and side (ipsi/contra) as within-subjects variables and CsCh expression as the factor	±CsCh: p = 0.978; legs: p = 0.230; side: p = 0.0617; interaction between legs and ±CsCh: p = 0.826; interaction between side and ±CsCh: p = 0.285
Figure 4C (right)	Mixed ANOVA with leg identity (ipsi front/mid/hind, contra front/mid/hind) as the within-subjects variable and CsCh expression as the factor	±CsCh: p = 0.978; legs: p = 0.449; interaction between legs and ±CsCh: p = 0.00203
Figure 4C (right)	Post-hoc Tukey-Kramer tests comparing CsCh+ and no-CsCh for each leg	c-front: p = 0.117; c-mid: p = 0.107; c-hind: p = 0.201; i-front: p = 0.0615; i-mid: p = 0.102; i-hind: p = 0.00856
Figure 4D	Two-sample t-tests comparing GtACR1+ and no-GtACR1	rotational velocity: p = 0.0730; forward velocity: p = 0.436
Figure 4E	Mixed ANOVA with legs (front/mid/hind) and side (ipsi/contra) as within-subjects variables and GtACR1 expression as the factor	±GtACR1: p = 0.180; legs: p = 0.998; side: p = 0.894; interaction between legs and ±GtACR1: p = 0.384; interaction between side and ±GtACR1: p = 0.0429
Figure 4E	Post-hoc Tukey-Kramer tests comparing GtACR1+ and no-GtACR1 for each side	ipsi: p = 0.0336; contra: p = 0.234
Figure 4E	Mixed ANOVA with leg identity (ipsi front/mid/hind, contra front/mid/hind) as the within-subjects variable and GtACR1 expression as the factor	±GtACR1: p = 0.180; legs: p = 0.999; interaction between legs and ±GtACR1: p = 0.00385
Figure 4E	Post-hoc Tukey-Kramer tests comparing GtACR1+ and no-GtACR1 for each leg	c-front: p = 0.713; c-mid: p = 0.203; c-hind: p = 0.176, i-front: p = 0.108; i-mid: p = 0.0143; i-hind: p = 0.0828
Figure 4F (left)	Mixed ANOVA with legs (front/mid/hind) and side (ipsi/contra) as within-subjects variables and GtACR1 expression as the factor	±GtACR1: p = 0.251; legs: $p = 2.30\times10^{-6}$; side: p = 0.511; interaction between legs and ±GtACR1: p = 0.319; interaction between side and ±GtACR1: p = 0.0713
Figure 4F (left)	Post-hoc Tukey-Kramer tests comparing GtACR1+ and no-GtACR1 for each side	ipsi: p = 0.0356; contra: p = 0.491
Figure 4F (left)	Mixed ANOVA with leg identity (ipsi front/mid/hind, contra front/mid/hind) as the within-subjects variable and GtACR1 expression as the factor	±GtACR1: p = 0.251; legs: $p = 1.42\times10^{-4}$; interaction between legs and ±GtACR1: p = 0.0579
Figure 4F (left)	Post-hoc Tukey-Kramer tests comparing GtACR1+ and no-GtACR1 for each leg	c-front: p = 0.575; c-mid: p = 0.450; c-hind: p = 0.718; i-front: p = 0.00705; i-mid: p = 0.230; i-hind: p = 0.322
Figure 4F (right)	Mixed ANOVA with legs (front/mid/hind) and side (ipsi/contra) as within-subjects variables and GtACR1 expression as the factor	±GtACR1: p = 0.324; legs: p = 0.00962; side: p = 0.768; interaction between legs and ±GtACR1: p = 0.399; interaction between side and ±GtACR1: p = 0.0390
Figure 4F (right)	Post-hoc Tukey-Kramer tests comparing GtACR1+ and no-GtACR1 for each side	ipsi: p = 0.0546; contra: p = 0.165
Figure 4F (right)	Mixed ANOVA with leg identity (ipsi front/mid/hind, contra front/mid/hind) as the within-subjects variable and GtACR1 expression as the factor	±GtACR1: p = 0.324; legs: p = 0.299; interaction between legs and ±GtACR1: p = 0.00383
Figure 4F (right)	Post-hoc Tukey-Kramer tests comparing GtACR1+ and no-GtACR1 for each leg	c-front: p = 0.780; c-mid: p = 0.160; c-hind: p = 0.136; i-front: p = 0.355; i-mid: p = 0.0102; i-hind: p = 0.106
Figure 4G	Two-sample t-tests comparing depolarization or hyperpolarization with no-stimulation trials	rotational velocity, hyperpol: p = 0.00257; rotational velocity, depol: p = 0.0404; forward velocity, hyperpol: p = 0.828; depol: p = 0.695

Figure 4H	Mixed ANOVA with legs (front/mid/hind) and side (ipsi/contra) as within-subjects variables and depol/hyperpol as the factor	depol/hyperpol: p = 0.0177; legs: p = 0.775; side: p = 0.137; interaction between legs and depol/hyperpol: p = 0.172; interaction between side and depol/hyperpol: p = 0.00703
Figure 4H	Post-hoc Tukey-Kramer tests comparing depol/hyperpol for each side	ipsi: p = 0.817; contra: p = 2.93×10^{-4}
Figure 4H	Mixed ANOVA with leg identity (ipsi front/mid/hind, contra front/mid/hind) as the within-subjects variable and depol/hyperpol as the factor	depol/hyperpol: p = 0.0177; legs: p = 0.197; interaction between legs and depol/hyperpol: p = 6.16×10^{-4}
Figure 4H	Post-hoc Tukey-Kramer tests comparing depol/hyperpol for each leg	c-front: p = 0.00722; c-mid: p = 1.15×10^{-4}; c-hind: p = 0.00391; i-front: p = 0.270; i-mid: p = 0.772; i-hind: p = 0.643
Figure 4I (left)	Mixed ANOVA with legs (front/mid/hind) and side (ipsi/contra) as within-subjects variables and depol/hyperpol as the factor	depol/hyperpol: p = 0.120; legs: p = 0.0845; side: p = 0.0377; interaction between legs and depol/hyperpol: p = 0.0725; interaction between side and depol/hyperpol: p = 0.0300
Figure 4I (left)	Post-hoc Tukey-Kramer tests comparing depol/hyperpol for each side	ipsi: p = 0.870; contra: p = 0.0290
Figure 4I (left)	Mixed ANOVA with leg identity (ipsi front/mid/hind, contra front/mid/hind) as the within-subjects variable and depol/hyperpol as the factor	depol/hyperpol: p = 0.120; legs: p = 0.00908; interaction between legs and depol/hyperpol: p = 0.00760
Figure 4I (left)	Post-hoc Tukey-Kramer tests comparing depol/hyperpol for each leg	c-front: p = 0.144; c-mid: p = 0.00690; c-hind: p = 0.167; i-front: p = 0.469; i-mid: p = 0.0373; i-hind: p = 0.962
Figure 4I (right)	Mixed ANOVA with legs (front/mid/hind) and side (ipsi/contra) as within-subjects variables and depol/hyperpol as the factor	depol/hyperpol: p = 0.197; legs: p = 0.371; side: p = 0.705; interaction between legs and depol/hyperpol: p = 0.853; interaction between side and depol/hyperpol: p = 0.0314
Figure 4I (right)	Post-hoc Tukey-Kramer tests comparing depol/hyperpol for each side	ipsi: p = 0.0317; contra: p = 0.661
Figure 4I (right)	Mixed ANOVA with leg identity (ipsi front/mid/hind, contra front/mid/hind) as the within-subjects variable and depol/hyperpol as the factor	depol/hyperpol: p = 0.197; legs: p = 0.554; interaction between legs and depol/hyperpol: p = 0.171
Figure 4I (right)	Post-hoc Tukey-Kramer tests comparing depol/hyperpol for each leg	c-front: p = 0.180; c-mid: p = 0.0420; c-hind: p = 0.0436; i-front: p = 0.412; i-mid: p = 0.854; i-hind: p = 0.561
Figure 5E (left)	Paired t-test comparing decreasing/increasing forward velocity	DNa02: p = 0.205; DNg13: p = 0.0158
Figure 5E (right)	Two-sample t-test comparing increasing – decreasing forward velocity difference between DNa02 and DNg13	p = 0.0101
Figure 6A (left)	One-sample t-test, time of peak different from 0	DNa02: p = 2.85×10^{-4}; DNg13: p = 5.36×10^{-4}
Figure 6A (right)	One-sample t-test, time of peak different from 0	DNa02: p = 0.00250; DNg13: p=7.24×10^{-4}
Figure 6B	Two-sample t-test on the full-width at half-maximum for DNa02 vs. DNg13	p = 0.00520
Figure 6C (right)	Paired t-test for each pair of legs comparing the ipsilateral and contralateral full-width at half-maximum	front: p = 1.31×10^{-76}; mid: p = 8.08×10^{-84}; hind: p = 4.95×10^{-93}
Figure S1B	For each leg, parametric Watson-Williams multi-sample test for equal means	out-front: p = 0.0277; out-mid: p = 1.94×10^{-6}; out-hind: p = 0.943; in-front: p = 5.55×10^{-16}; in-mid: p = 1.42×10^{-4}; in-hind: p = 0.963
Figure S1C (left)	Two-way ANOVA with legs and pivot/swerve as factors	pivot/swerve: p = 3.68×10^{-15}; leg identity: p = 1.01×10^{-17}; interaction: p = 0.0198
Figure S1C (left)	Post-hoc Tukey-Kramer tests comparing pivots and swerves for each leg	out-front: p = 4.28×10^{-8}; out-mid: p = 0.000744; out-hind: p = 0.000200; in-front: p = 0.000243; in-mid: p = 5.29×10^{-5}; in-hind: p = 0.00524
Figure S1C (middle)	Two-way ANOVA with legs and pivot/swerve as factors	pivot/swerve p=1.75×10^{-27}, leg identity p=0, interaction: p=0.794
Figure S1C (middle)	Post-hoc Tukey-Kramer tests comparing pivots and swerves for each leg	out-front: p = 0.00220; out-mid: p = 4.75×10^{-6}; out-hind: p = 0.00120; in-front: p = 0.0235; in-mid: p = 1.54×10^{-5}; in-hind: p = 0.00143
Figure S1C (right)	For each leg, parametric Watson-Williams multi-sample test for equal means	out-front: p = 2.22×10^{-16}, out-mid: p = 2.22×10^{-16}; out-hind: p = 0.445; in-front: p = 4.13×10^{-12}; in-mid: p = 9.94×10^{-5}; in-hind: p = 0.140;
Figures S2C and S2F	Paired t-tests comparing walking and not-walking	DNa01: p = 0.000595; DNa02: p = 0.000558; DNb05: p = 0.00644; DNb06: p = 0.00301; DNg13: p = 0.0138; DNg14: p = 0.302; DNg15: p = 0.167; DNg16: p = 0.607; DNg31: p = 0.00370; DNg34: p = 5.60×10^{-6}; DNp05: p = 0.0290; DNp09: p = 0.0715; DNp11: p = 0.0520; DNp12: p = 0.00234; DNp18: p = 0.0503; DNp32: p = 0.0628
Figure S6A	Two-sample t-tests comparing CsCh+ and no-CsCh	rotational velocity: p = 0.341; forward velocity: p = 0.00564
Figure S6B	Mixed ANOVA with legs (front/mid/hind) and side (ipsi/contra) as within-subjects variables and CsCh expression as the factor	±CsCh: p = 3.29×10^{-5}; legs: p = 0.496; side: p = 0.350; interaction between legs and ±CsCh: p = 0.852; interaction between side and ±CsCh: p = 0.891
Figure S6B	Post-hoc Tukey-Kramer tests comparing CsCh+ and no-CsCh for each side	ipsi: p = 9.95×10^{-5}; contra: p = 3.82×10^{-4}
Figure S6B	Mixed ANOVA with leg identity (ipsi front/mid/hind, contra front/mid/hind) as the within-subjects variable and CsCh expression as the factor	±CsCh: p = 3.29×10^{-5}; legs: p = 0.702; interaction between legs and ±CsCh: p = 0.949
Figure S6B	Post-hoc Tukey-Kramer tests comparing CsCh+ and no-CsCh for each leg	c-front: p = 0.00147; c-mid: p = 6.55×10^{-4}; c-hind: p = 8.72×10^{-5}; i-front: p = 0.00191; i-mid: p = 0.00510; i-hind: p = 2.99×10^{-4}
Figure S6C (left)	Mixed ANOVA with legs (front/mid/hind) and side (ipsi/contra) as within-subjects variables and CsCh expression as the factor	±CsCh: p = 3.94×10^{-6}; legs: p = 0.126; side: p = 0.318; interaction between legs and ±CsCh: p = 0.377; interaction between side and ±CsCh: p = 0.316

Figure S6C (left)	Post-hoc Tukey-Kramer tests comparing CsCh+ and no-CsCh for each side	ipsi: $p = 2.61\times10^{-5}$; contra: $p = 1.18\times10^{-5}$
Figure S6C (left)	Mixed ANOVA with leg identity (ipsi front/mid/hind, contra front/mid/hind) as the within-subjects variable and CsCh expression as the factor	±CsCh: $p = 3.94\times10^{-6}$; legs: p = 0.0954; interaction between legs and ±CsCh: p = 0.205
Figure S6C (left)	Post-hoc Tukey-Kramer tests comparing CsCh+ and no-CsCh for each leg	c-front: p = 0.00121; c-mid: p = 0.00841; c-hind: p = 0.0647; i-front: $p = 1.91\times10^{-5}$; i-mid: p = 0.0177; i-hind: p = 0.00318
Figure S6C (right)	Mixed ANOVA with legs (front/mid/hind) and side (ipsi/contra) as within-subjects variables and CsCh expression as the factor	±CsCh: p = 0.0103 legs: p = 0.00123; side: p = 0.220; interaction between legs and ±CsCh: p = 0.0306; interaction between side and ±CsCh: p = 0.508
Figure S6C (right)	Post-hoc Tukey-Kramer tests comparing CsCh+ and no-CsCh for each side	ipsi: p = 0.115; contra: p = 0.0672
Figure S6C (right)	Mixed ANOVA with leg identity (ipsi front/mid/hind, contra front/mid/hind) as the within-subjects variable and CsCh expression as the factor	±CsCh: p = 0.0103; legs: p = 0.00368; interaction between legs and ±CsCh: p = 0.00171
Figure S6C (right)	Post-hoc Tukey-Kramer tests comparing CsCh+ and no-CsCh for each leg	c-front: p = 0.0637; c-mid: p = 0.181; c-hind: $p = 4.16\times10^{-4}$; i-front: p = 0.437; i-mid: p = 0.0415; i-hind: p = 0.0666
Figure S6D	Two-sample t-tests comparing GtACR1+ and no-GtACR1	rotational velocity: p = 0.731; forward velocity: p = 0.687
Figure S6E	Mixed ANOVA with legs (front/mid/hind) and side (ipsi/contra) as within-subjects variables and GtACR1 expression as the factor	±GtACR1: p = 0.0641 legs: p = 0.948; side: p = 0.333; interaction between legs and ±GtACR1: p = 0.589; interaction between side and ±GtACR1: p = 0.588
Figure S6F (left)	Mixed ANOVA with legs (front/mid/hind) and side (ipsi/contra) as within-subjects variables and GtACR1 expression as the factor	±GtACR1: p = 0.645 legs: p = 0.00194; side: p = 0.301; interaction between legs and ±GtACR1: p = 0.911; interaction between side and ±GtACR1: p = 0.927
Figure S6F (left)	Post-hoc Tukey-Kramer tests comparing GtACR1+ and no-GtACR1 for each side	ipsi: p = 0.754; contra: p = 0.709
Figure S6F (left)	Mixed ANOVA with leg identity (ipsi front/mid/hind, contra front/mid/hind) as the within-subjects variable and GtACR1 expression as the factor	±GtACR1: p = 0.645; legs: p = 0.00136; interaction between legs and ±GtACR1: p = 0.726
Figure S6F (left)	Post-hoc Tukey-Kramer tests comparing GtACR1+ and no-GtACR1 for each leg	c-front: p = 0.143; c-mid: p = 0.996; c-hind: p = 0.997; i-front: p = 0.198; i-mid: p = 0.466; i-hind: p = 0.390
Figure S6F (right)	Mixed ANOVA with legs (front/mid/hind) and side (ipsi/contra) as within-subjects variables and GtACR1 expression as the factor	±GtACR1: p = 0.0118 legs: p = 0.0716; side: p = 0.333; interaction between legs and ±GtACR1: p = 0.589; interaction between side and ±GtACR1: p = 0.427
Figure S6F (right)	Post-hoc Tukey-Kramer tests comparing GtACR1+ and no-GtACR1 for each side	ipsi: p = 0.0534; contra: p = 0.392
Figure S6F (right)	Mixed ANOVA with leg identity (ipsi front/mid/hind, contra front/mid/hind) as the within-subjects variable and GtACR1 expression as the factor	±GtACR1: p = 0.0118; legs: p = 0.123; interaction between legs and ±GtACR1: p = 0.697
Figure S6F (right)	Post-hoc Tukey-Kramer tests comparing GtACR1+ and no-GtACR1 for each leg	c-front: p = 0.423; c-mid: p = 0.342; c-hind: p = 0.990; i-front: p = 0.146; i-mid: p = 0.138; i-hind: p = 0.00482
Figure S6G (left)	Parametric two-way ANOVA for circular data with CsCh expression and leg identity (ipsi front/mid/hind, contra front/mid/hind) as factors	Unilateral vs. no-CsCh: ±CsCh: $p=1.33\times10^{-6}$; leg identity: $p=3.37\times10^{-10}$; interaction between leg identity and ±CsCh: $p=8.08\times10^{-9}$<br>Bilateral vs. no-CsCh: ±CsCh: p=0.277; leg identity: p=0.222; interaction between leg identity and ±CsCh: p=0.0948
Figure S6G (left)	Post-hoc parametric Watson-Williams multisample tests for equal means, comparing ±CsCh for each leg, with Holm-Bonferroni correction for multiple comparisons	Unilateral vs. no-CsCh: c-front: p = 0.00310 *; c-mid: p = 0.0164 *; c-hind: p = 0.0253 *; i-front: $p = 4.63\times10^{-4}$ *; i-mid: $p = 8.01\times10^{-4}$ *; i-hind: p = 0.00751 * (where * denotes significance after multiple comparisons correction, α = 0.05)
Figure S6G (middle)	Parametric two-way ANOVA for circular data with GtACR1 expression and leg identity (ipsi front/mid/hind, contra front/mid/hind) as factors	Unilateral vs. no-GtACR1: ±GtACR1: $p=7.87\times10^{-4}$; leg identity: p=0.808; interaction between leg identity and ±GtACR1: p=0.00831<br>Bilateral vs. no-GtACR1: ±GtACR1: p=0.522; leg identity: p=0.134; interaction between leg identity and ±GtACR1: p=0.904
Figure S6G (middle)	Post-hoc parametric Watson-Williams multisample tests for equal means, comparing ±GtACR1 for each leg, with Holm-Bonferroni correction for multiple comparisons	Unilateral vs. no-GtACR1: c-front: p = 0.0433; c-mid: p = 0.0702; c-hind: p = 0.233; i-front: p = 0.0258; i-mid: p = 0.116; i-hind: p = 0.449 (where * denotes significance after multiple comparisons correction, α = 0.05)
Figure S6G (right)	Parametric two-way ANOVA for circular data with depol/hyperpol and leg identity (ipsi front/mid/hind, contra front/mid/hind) as factors	depol/hyperpol: $p = 9.82\times10^{-10}$; leg identity: p = 0.412; interaction: $p = 8.14\times10^{-6}$
Figure S6G (right)	Post-hoc parametric Watson-Williams multisample tests for equal means, comparing depol/hyperpol for each leg, with Holm-Bonferroni correction for multiple comparisons	c-front: $p = 2.85\times10^{-4}$ *; c-mid: p = 0.0123 *; c-hind: p = 0.552; i-front: p = 0.00189 *; i-mid: p = 0.0132 *; i-hind: p = 0.798 (where * denotes significance after multiple comparisons correction, α = 0.05)
Figure S7A	Paired t-test comparing walking and not walking	DNa02: p = 0.00229; DNg13: 4.59×10^{-5}
Figure S7C (right)	paired t-test on difference in correlation coefficients, turning versus not-turning	p = 0.0109
Figure S7D (right)	paired t-test on difference in correlation coefficients, turning versus not-turning	p = 0.00767
